# A sociotechnical framework to assess patient-facing eHealth tools: results of a modified Delphi process

**DOI:** 10.1038/s41746-023-00982-w

**Published:** 2023-12-15

**Authors:** Christine Jacob, Johan Lindeque, Roman Müller, Alexander Klein, Thomas Metcalfe, Samantha L. Connolly, Florian Koerber, Roma Maguire, Fabrice Denis, Sabina C. Heuss, Marc K. Peter

**Affiliations:** 1 University of Applied Sciences Northwestern Switzerland (FHNW), Olten, Switzerland; 2 Personalized Healthcare, Pharma Product Development, F Hoffmann-La Roche Ltd, Basel, Switzerland; 3 Center for Healthcare Organization and Implementation Research, VA Boston Healthcare System, Boston, MA USA; 4 Department of Psychiatry, Harvard Medical School, Boston, MA USA; 5 IU Internationale Hochschule, Erfurt, Germany; 6 Flying Health GmbH, Berlin, Germany; 7 Department of Computer and Information Sciences, University of Strathclyde, Glasgow, United Kingdom; 8 Institut Inter-régional de Cancérologie Jean Bernard, ELSAN, Le Mans, France; 9 Institute for Smarthealth, Le Mans, France

**Keywords:** Health care economics, Health care

## Abstract

Among the thousands of eHealth tools available, the vast majority do not get past pilot phases because they cannot prove value, and only a few have been systematically assessed. Although multiple eHealth assessment frameworks have been developed, these efforts face multiple challenges. This study aimed to address some of these challenges by validating and refining an initial list of 55 assessment criteria based on previous frameworks through a two-round modified Delphi process with in-between rounds of interviews. The expert panel (*n* = 57) included participants from 18 countries and 9 concerned parties. A consensus was reached on 46 criteria that were classified into foundational and contextual criteria. The 36 foundational criteria focus on evaluating the eHealth tool itself and were grouped into nine clusters: technical aspects, clinical utility and safety, usability and human centricity, functionality, content, data management, endorsement, maintenance, and developer. The 10 contextual criteria focus on evaluating the factors that vary depending on the context the tool is being evaluated for and were grouped into seven clusters: data-protection compliance, safety regulatory compliance, interoperability and data integration, cultural requirements, affordability, cost-benefit, and implementability. The classification of criteria into foundational and contextual helps us assess not only the quality of an isolated tool, but also its potential fit in a specific setting. Criteria subscales may be particularly relevant when determining the strengths and weaknesses of the tool being evaluated. This granularity enables different concerned parties to make informed decisions about which tools to consider according to their specific needs and priorities.

## Introduction

eHealth tools, which are defined as the cost-effective and secure use of information and communications technologies in support of health and health-related fields^1^, have promising potential ranging from efficiency gains such as cutting costs and optimizing clinical workflows to achieving better health outcomes and quality of care services^2–6^. The surge in eHealth adoption during the COVID-19 pandemic showed how useful these tools could be; however, this increase in adoption and interest was not necessarily sustained after the pandemic slowed down^7^. Among the thousands of eHealth tools available, the vast majority do not get past pilot phases because they cannot prove value or because of implementation barriers^8,9^, and only a few have been systematically assessed or evaluated^10–12^. Furthermore, previous studies that assessed existing eHealth tools concluded that many overlooked features that were deemed important for the tool to successfully meet its intended objectives, had key technical deficiencies, or showed modest clinical utility^13–15^. This makes it particularly challenging for the concerned parties, including patients, healthcare providers, payers, and other industry players, such as pharma companies, to identify quality eHealth tools in this crowded space^13,16^, especially due to the lack of standardized assessment approaches^13,16,17^.

Although multiple eHealth assessment frameworks and initiatives have been developed over the last decade, showing a lack of standardization in this field, these efforts face multiple challenges^11,18,19^. An initial systematic review of this topic^18^ found that many of the existing assessment frameworks have not been validated with relevant concerned parties^13,20,21^, resulting in assessment processes that may not always reflect the real-world needs of diverse populations^13^. In many cases, these frameworks are conceptual, without granular guidance on how to practically use them in routine decision-making^19,21–23^. Furthermore, some of these frameworks overlook important assessment criteria, resulting in incomplete or issue-specific assessment frameworks^24–28^.

Even from a regulatory perspective, the lack of clarity and absence of institutionalized quality controls in many countries make a comprehensive definition of the assessment criteria more challenging^12,29–32^. Although certification and regulatory approvals are necessary, especially for tools with higher safety risks, they are not always sufficient. For instance, even though common labels may classify an eHealth tool as a medical device, it may still include the warning in fine print that it is intended for entertainment only, showing a lack of accountability and creating confusion^28^. In addition to gray areas in existing regulatory oversight initiatives, for example, the US Food and Drug Administration (FDA) applies regulatory oversight only to a small subset of eHealth tools that qualify as medical devices and potentially pose a risk to patient safety^17,33^. Similarly, the European regulatory system offers the Conformité Européenne (CE) mark; however, while the mark implies that these tools are compliant with European legislation, they only need to demonstrate safety and performance but not clinical efficacy^33^. A recent study that assessed user reviews of DiGA-certified apps in Germany (referred to as *Digitale Gesundheitsanwendungen* in German, meaning digital health applications, for prescription with costs covered by standard statutory health insurance) showed that many users criticized the limited value-add of these tools, implying that the certification process and emphasis on clinical evidence may not be effectively translated into perceived value among users^34^. These regulatory gaps suggest that safety, efficacy, and ethical compliance of certified eHealth tools cannot always be guaranteed^35^.

As the lack of validation and assessor diversity are two of the key challenges facing eHealth assessment efforts, one of the main aims of this study was to validate the findings of the systematic review that we conducted^18^ through a diverse expert panel using a modified Delphi process. The goal was to pressure test the initial list of criteria with experts from all relevant concerned parties to define which criteria are must-have criteria, which criteria are not as important and can be considered as nice-to-have, and whether there are any criteria that were missing and should be added to the validated framework. Furthermore, we wanted to address the challenge of contextuality by including contextual criteria in our initial list to validate their relevance to experts. We aimed to address the challenge of the lack of practicability of some of the previous initiatives by going beyond validating and complementing the list of criteria, expanding the dialogue with the experts, and discussing ways to make the proposed assessment instrument as usable and accessible as possible to support them in their day-to-day decision-making.

We adopted the World Health Organization (WHO) definition of eHealth as “the cost-effective and secure use of information and communications technologies in support of health and health-related fields, including health care services, health surveillance, health literature, and health education, knowledge, and research”^1^. Furthermore, we focused on patient-facing eHealth tools, including self-management tools and remote eHealth solutions, rather than tools used exclusively by care providers (e.g., video conferencing software used to meet with other providers or the use of electronic health record software) or health data analytics systems used at the population level.

While some existing assessment initiatives focus on curating, certifying, or accrediting eHealth tools with the goal of helping potential customers differentiate between low- and high-quality offerings^16^, the focus of this work is to equip decision makers with an assessment instrument to help support their decision-making according to their specific needs and priorities in the specific contexts in which they are considering a tool. The findings from this study will help inform multiple concerned parties, including clinicians, pharmaceutical executives, insurance professionals, investors, technology providers, and policymakers, by presenting them with a validated sociotechnical framework that encompasses the different criteria used to assess patient-facing eHealth tools not only from a technological perspective, but also from a contextual perspective. This can guide them in making informed decisions about which tools to use, endorse to patients, invest in, partner with, or reimburse based on their potential quality and their fit into the specific context for which they are being evaluated.

## Results

### Expert panel characteristics

A total of 120 experts who had been vetted for eHealth expertise were contacted, and 57 (47.5%) agreed to participate in the research. The international expert panel included experts from 9 concerned parties and 18 countries. Several participants had multiple roles and backgrounds or were active in more than one geography; hence, there was an overlap in some of these sample characteristics. Table 1 presents the characteristics of the sample.

**Table 1:** Sample characteristics.

Demographics and characteristics	Values, *n* (%)
Background (Some participants had multiple backgrounds)	Regulatory Expert = 1 (2%) Insurance Expert = 3 (5%) Investor = 4 (7%) Patient Advocate = 8 (14%) Pharma = 10 (18%) Researcher = 13 (23%) Clinician = 14 (25%) Tech Provider = 15 (26%) eHealth Expert = 21 (37%)
Gender	Female = 23 (40%) Male = 34 (60%)
Location (Some participants were active in multiple countries)	Australia = 1 (2%) Belgium = 1 (2%) Bulgaria = 1 (2%) Spain =1 (2%) Italy = 1 (2%) Singapore = 1 (2%) Tunisia = 1 (2%) Egypt = 2 (4%) Finland = 2 (4%) Ireland = 2 (4%) Netherlands = 2 (4%) United Arab Emirates = 3 (5%) France = 4 (7%) Germany = 5 (9%) Canada = 6 (11%) United Kingdom = 8 (14%) United States = 10 (18%) Switzerland = 21 (37%)

### Expert consensus on the assessment criteria in round 1 survey

The experts voted on the relevance of an initial list of 55 criteria, of which 38 (69%) met the predefined 75% expert consensus, whereas 17 (31%) criteria did not meet the consensus. Participants provided additional comments, observations, and clarifications of the criteria during the round 1 survey and suggested the addition of nine new criteria. The proposed criteria included: 1) whether the tool’s content has been reviewed by patients, 2) whether the tool explicitly and easily enables users to delete their data, 3) whether the tool is reliable and available at all times and can handle high levels of traffic and usage with backup and recovery measures in case of downtime or system failures, 4) whether the tool allows for data sharing and segregation for research use, 5) data findability and retrievability including metadata definition, 6) whether the tool is usable and accessible in the intended clinical setting, 7) affordability of the tool taking into account the local socioeconomic context, 8) whether the tool differentiates between clinical and technical feedback and clearly channels clinical feedback that may pose a health risk through the proper channels, and 9) availability of phase-out scenarios if the tool provider stops maintaining it.

**Fig. 1:**
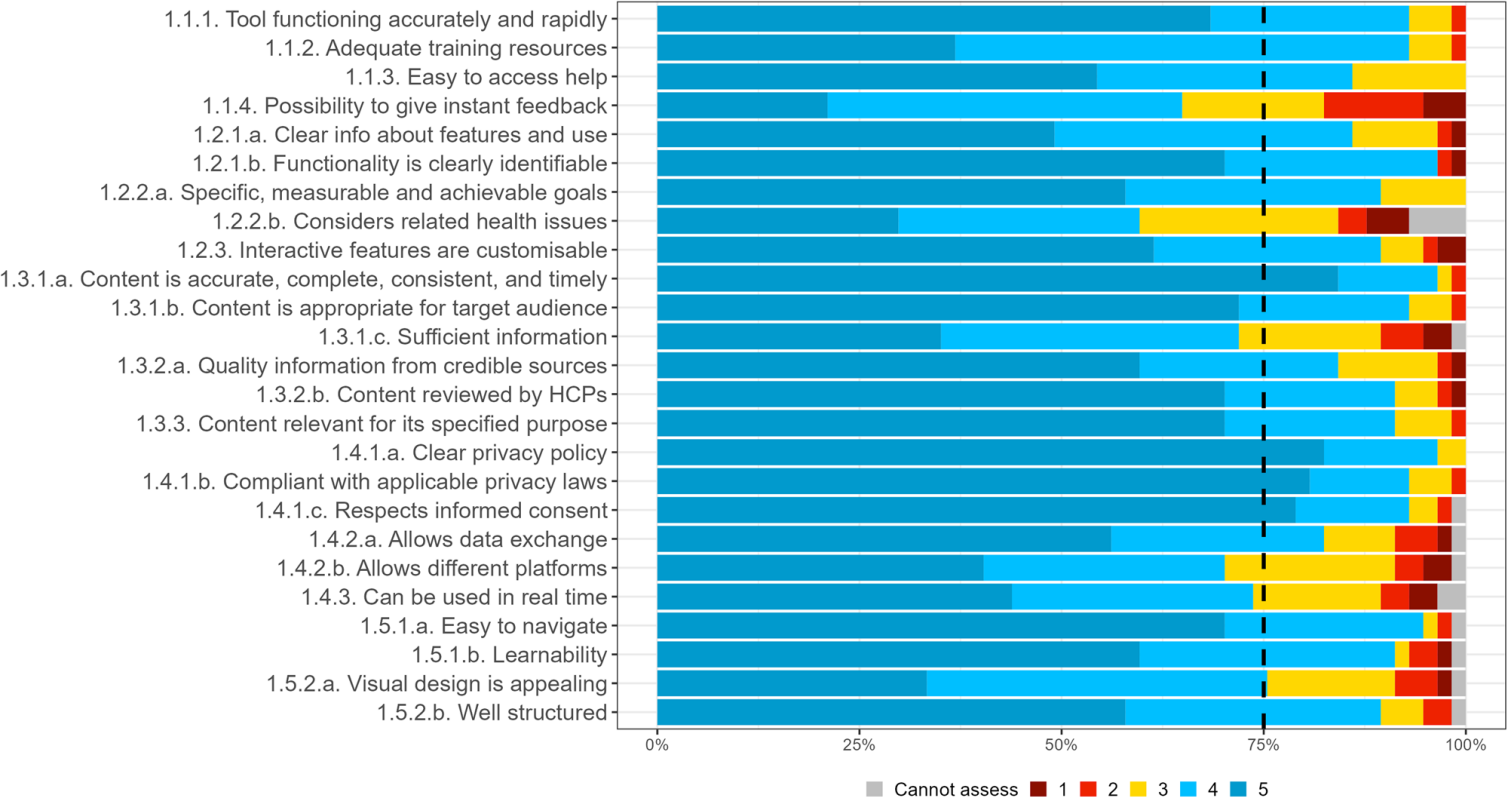
Expert consensus on technical assessment criteria in round 1 survey. Percentage of experts that voted cannot assess (grey), 1 (dark red), 2 (light red), 3 (yellow), 4 (light blue), 5 (dark blue). Where 1 signifies “I suggest this criterion is excluded”; and 5 signifies “This criterion is extremely relevant”.

**Fig. 2:**
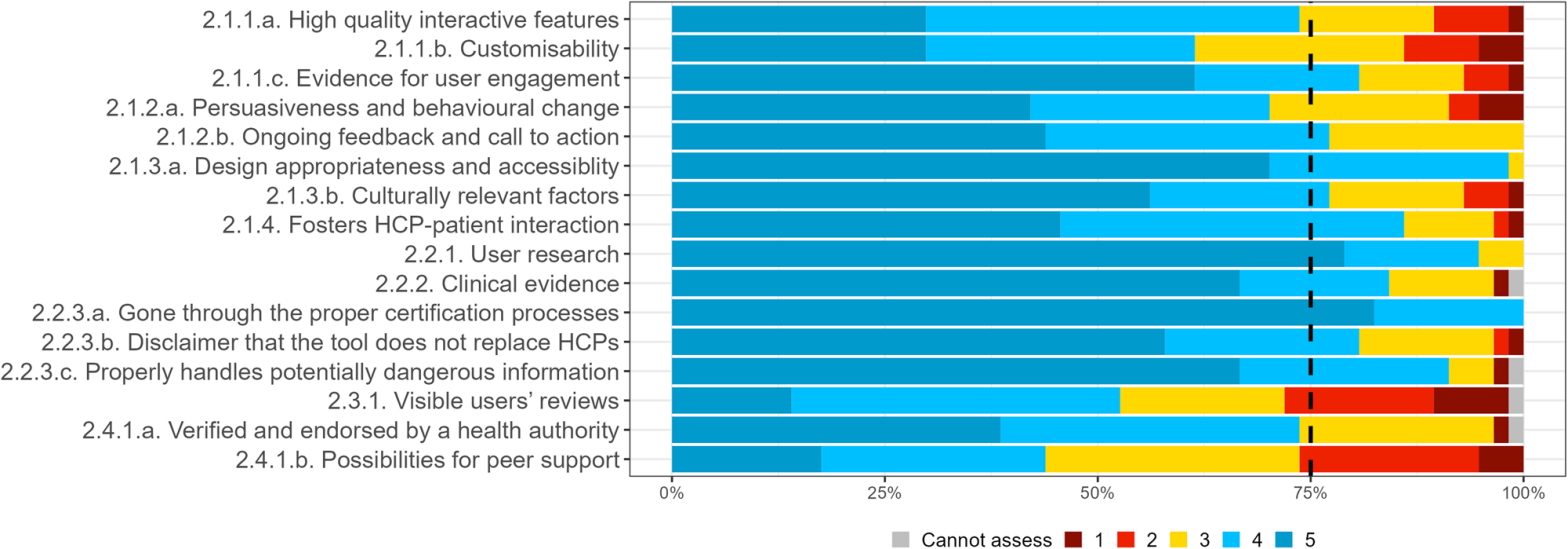
Expert consensus on social assessment criteria in round 1 survey. Percentage of experts that voted cannot assess (grey), 1 (dark red), 2 (light red), 3 (yellow), 4 (light blue), 5 (dark blue). Where 1 signifies “I suggest this criterion is excluded”; and 5 signifies “This criterion is extremely relevant”.

**Fig. 3:**
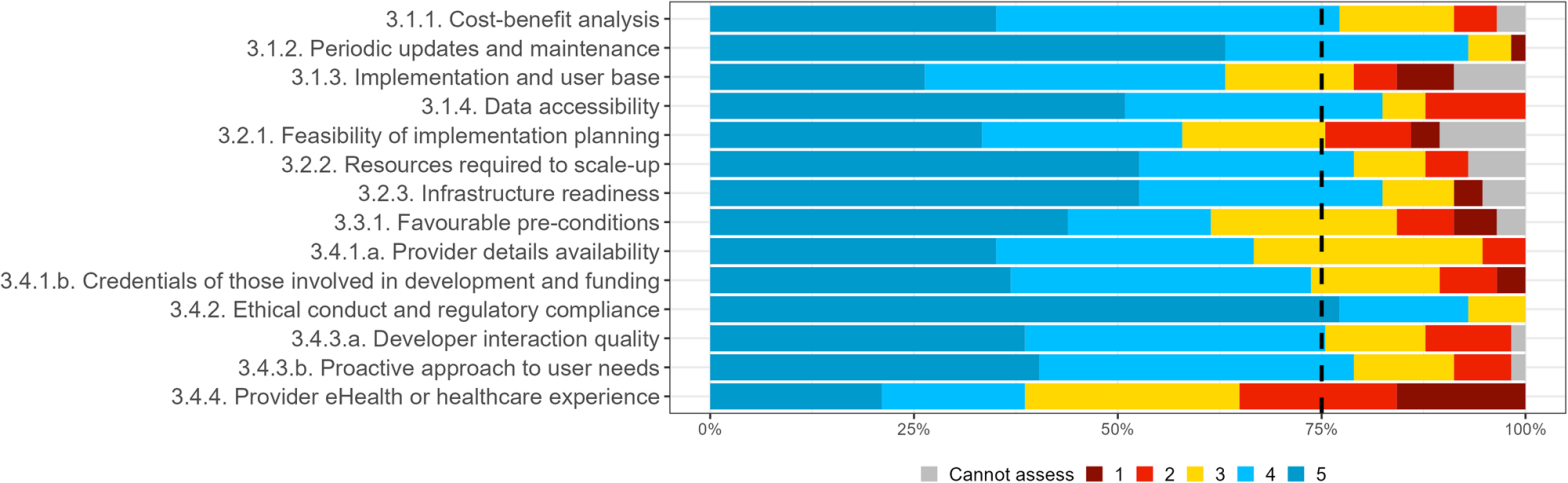
Expert consensus on organizational assessment criteria in round 1 survey. Percentage of experts that voted cannot assess (grey), 1 (dark red), 2 (light red), 3 (yellow), 4 (light blue), 5 (dark blue). Where 1 signifies “I suggest this criterion is excluded”; and 5 signifies “This criterion is extremely relevant”.

Supplementary Table 1 in the Supplementary Information file includes detailed statistics of the consensus and distribution of each criterion in the first survey, as well as expert agreement on the risk categories for which each criterion applies. Many of the criteria that did not meet the consensus were rather close to the 75% predefined consensus, and only two (4%) criteria received a consensus level of less than 50%. The IQR value for all criteria that met the consensus was 1 or less, indicating strong agreement among the experts. Figures 1–3 show expert consensus on technical, social, and organizational assessment criteria in the round 1 survey, respectively, where the dotted line shows the 75% consensus cut-off.

### Key themes discussed in the expert interviews

The focus of the interviews was to discuss the directional decisions and challenges that the experts expressed in the open-ended questions of Round 1 with the aim of making the proposed assessment instrument as usable and accessible as possible for all relevant concerned parties. A total of 55 experts participated in the interviews.

**Fig. 4:**
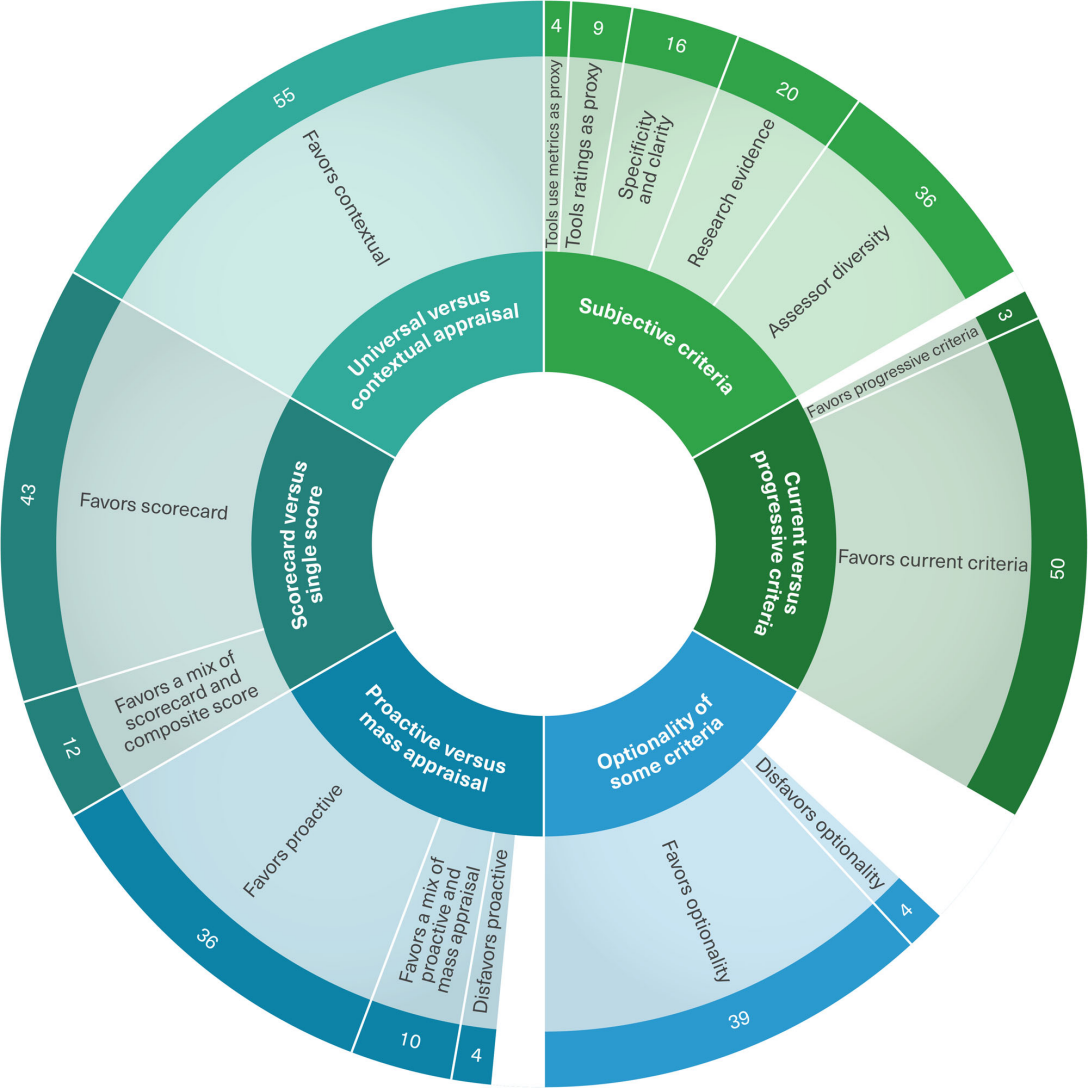
Key themes and their prevalence considering the key directional decisions and challenges.

There were six key themes that were discussed in the interviews: 1) universal criteria that do not change with context, versus the inclusion of contextual criteria, bearing in mind that this part of the assessment will be different for each context being considered; 2) the relevance of using a single score versus a scorecard to present the assessment results; 3) the relevance of a proactive appraisal that requires hands-on trial of the tool being assessed and getting in touch with the tool developers if needed, versus mass appraisal that mostly focuses on publicly available information; 4) the use of subjective criteria and how to minimize the subjectivity in the assessment without sacrificing important criteria that met the experts’ consensus; 5) the optionality of some criteria that met the expert consensus; 6) current versus progressive criteria, especially relating to criteria that met the consensus but may be viewed by some as shortsighted or a potential barrier to innovation. Figure 4 shows these key themes and their subsequent subthemes, reflecting the frequency of each theme (i.e., the number of participants who favored a specific direction). The white spaces in the visual reflect the experts that could not make up their mind about a specific key theme.

All experts (55/55, 100%) supported the inclusion of contextual criteria in the assessment instrument. Most experts favored a scorecard approach to present the results of the assessment (43/55, 78%), whereas some experts suggested a combination of a scorecard and a composite score to make comparability easier if the assessor was comparing several tools at the same time (12/55, 22%). Most experts favored a proactive appraisal that requires hands-on trial of the tool being assessed and getting in touch with the tool developers if needed (36/55, 65%), while some experts suggested a mix of mass and proactive appraisal (10/55, 18%), and only (4/55, 7%) disfavored the proactive approach, arguing that it requires too much effort and time.

When asked about how to minimize the subjectivity of some of the criteria that met the consensus, most experts recommended assessors’ diversity as a way to reflect the different views of diverse users when assessing a subjective criterion (34/55, 62%), as well as evidence and validation through published research about the tool being assessed (20/55, 36%), and clarity and specificity of assessor guidance to reach a common understanding of how to assess these criteria (16/55, 29%). Experts also suggested the use of some proxy criteria, such as a tool’s public user rating if a critical mass is achieved (9/55, 16%), and a tool’s usage metrics as an indication of its usability (4/55, 7%).

The challenge of whether to allow the optionality of some of the criteria that met the consensus was not easy to resolve, with several experts being undecided on whether to favor or disfavor a (not applicable) option for some criteria (12/55, 22%). However, the majority of experts acknowledged the vast variability of eHealth tools, which compels the optionality of some criteria even though they met the expert consensus (39/55, 71%), while (4/55, 7%) experts disfavored optionality, suggesting that all criteria that met the consensus should be mandatory in the assessment instrument. When asked whether they favor current versus progressive criteria, especially relating to criteria that met the consensus but may be viewed by some as shortsighted or a potential barrier to innovation, the vast majority of the experts favored current criteria to safeguard patient safety (50/55, 91%), while (3/55, 5%) experts favored a more progressive approach to enable and accelerate innovation. Supplementary Table 2 in the Supplementary Information file summarizes these key themes and subthemes, their frequencies, and sample expert quotes for each.

### Expert consensus on the assessment criteria in round 2 survey

**Fig. 5:**
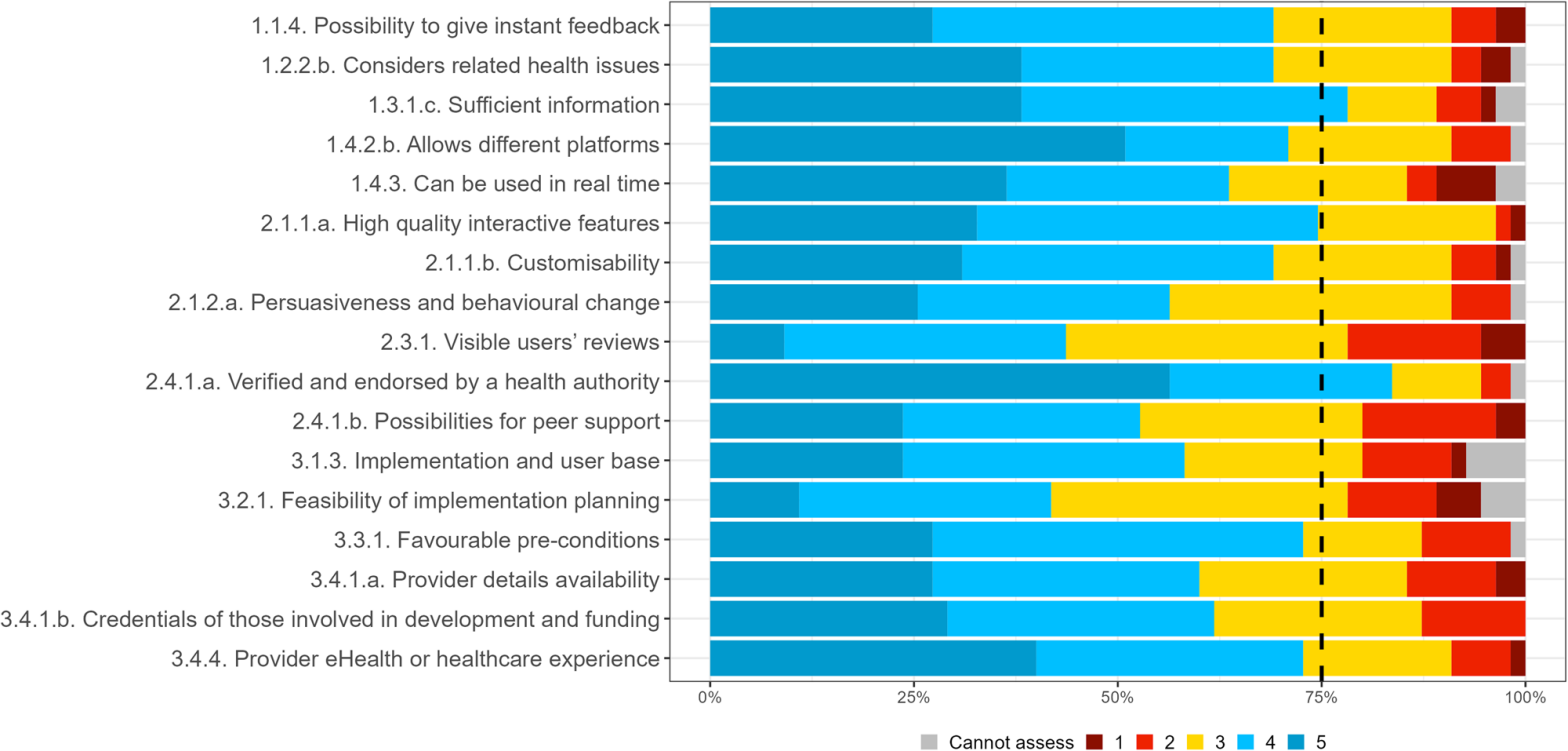
Expert consensus on assessment criteria that did not meet the consensus in the first round. Percentage of experts that voted cannot assess (grey), 1 (dark red), 2 (light red), 3 (yellow), 4 (light blue), 5 (dark blue). Where 1 signifies “I suggest this criterion is excluded”; and 5 signifies “This criterion is extremely relevant”.

**Fig. 6:**
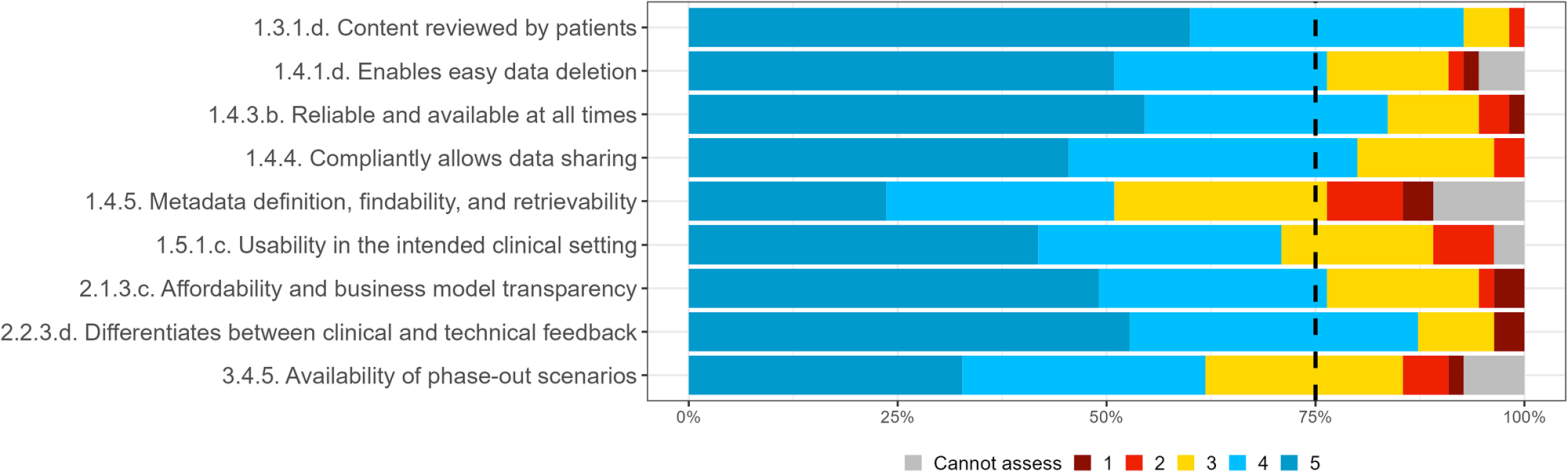
Expert consensus on new assessment criteria suggested by the experts in the first round. Percentage of experts that voted cannot assess (grey), 1 (dark red), 2 (light red), 3 (yellow), 4 (light blue), 5 (dark blue). Where 1 signifies “I suggest this criterion is excluded”; and 5 signifies “This criterion is extremely relevant”.

The round 2 survey was closed with 55 responses. Experts were asked to re-rate the 17 criteria whose initial ratings did not reach the inclusion thresholds in round 1, of which two met the 75% consensus as a result of the round 2 survey, and of the nine newly suggested criteria, six met the 75% consensus. The IQR value for all criteria that met the consensus was 1 or less, indicating strong agreement among the experts. Supplementary Table 3 in the Supplementary Information file includes detailed statistics of the distribution and consensus of each criterion included in round 2. Figure 5 shows expert consensus when re-rating the assessment criteria that did not meet the consensus in the first round, and Fig. 6 shows expert consensus on the new assessment criteria suggested by the experts in the first round, where the dotted line shows the 75% consensus cut-off.

## Discussion

The classification of criteria clusters into foundational and contextual criteria helps us answer two key questions: “what is the assessment of the quality of the tool itself regardless of its context” and “what is the assessment of the potential impact of the tool in a specific setting given its contextual fit?”. This sociotechnical approach, which has at its core the idea that the design and performance of any innovation can only be understood and improved if both ‘social’ and ‘technical’ aspects are brought together and treated as interdependent parts of a complex system^36^, goes beyond assessing isolated technologies and takes their intended context into account. At the same time, classifying the criteria into foundational and contextual makes the evaluation process more efficient for assessors appraising a tool for more than one potential context, as they only have to repeat the contextual assessment for each new context, whereas the assessment for the foundational criteria remains the same. It is worth noting, however, that the speed at which technologies are developing requires periodic revisions of the assessment of the foundational criteria to reflect how the tool being evaluated evolves as technology progresses.

**Fig. 7:**
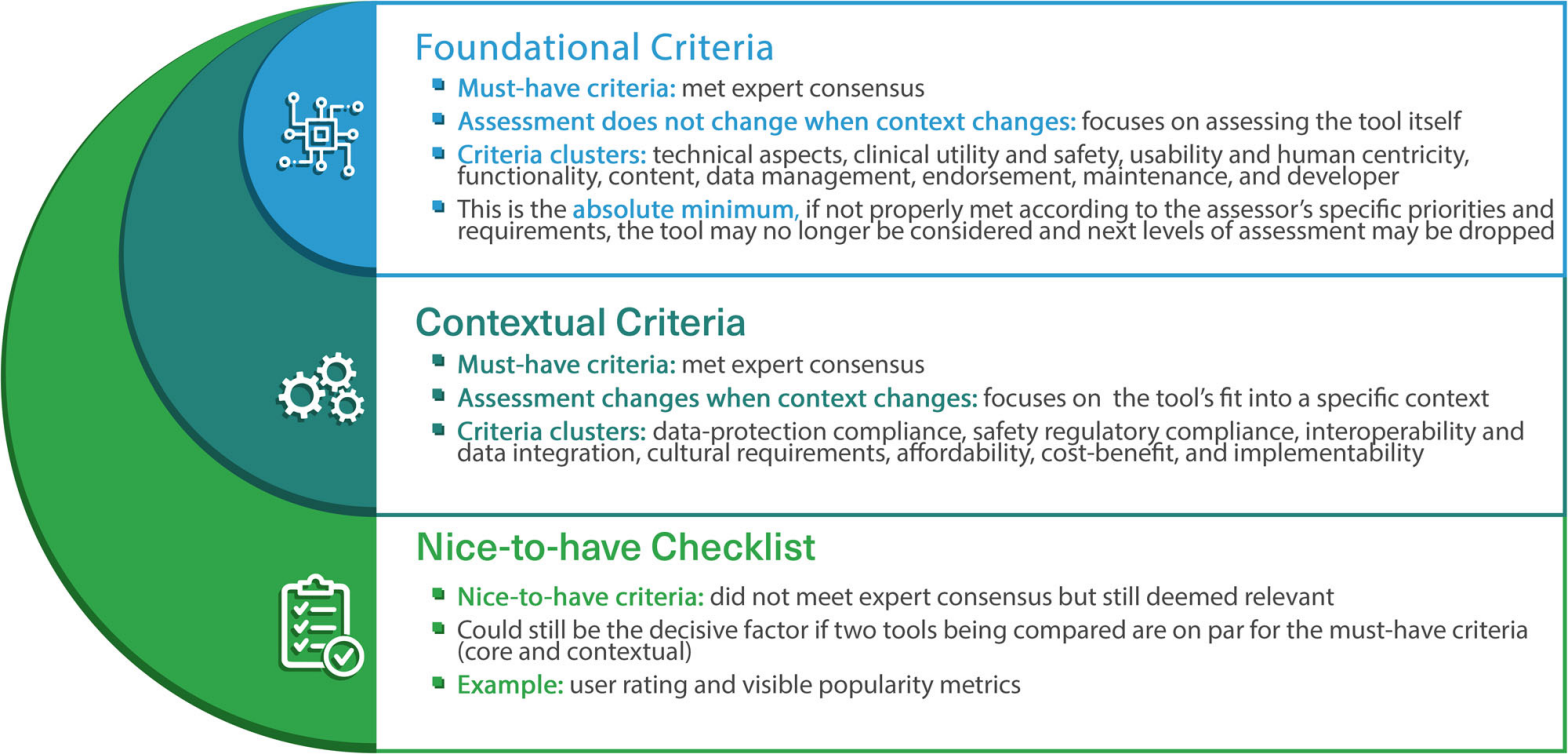
Criteria classification to core, contextual, and nice-to-have.

Figure 7 demonstrates this classification logic and differentiates between assessment criteria that are must-have because they met the expert consensus and the nice-to-have additional checklist that encompasses criteria that did not meet the consensus. The inclusion of the additional nice-to-have checklist was due to the fact that all criteria that did not meet the consensus (except for two criteria) had a consensus level of more than 50%, which means that at least every other expert rated it as very relevant or extremely relevant (4 or 5 on the Likert scale). This signifies that considering these additional criteria may still make sense in some cases, even if they are not considered as a must-have requirement.

The foundational criteria encompass nine clusters: technical aspects, clinical utility and safety, usability and human centricity, functionality, content, data management, endorsement, maintenance, and the developer. The contextual criteria encompass seven clusters: data-protection compliance, safety regulatory compliance, interoperability and data integration, cultural requirements, affordability, cost-benefit, and implementability. Some of these clusters may include more than one subcriterion.

**Fig. 8:**
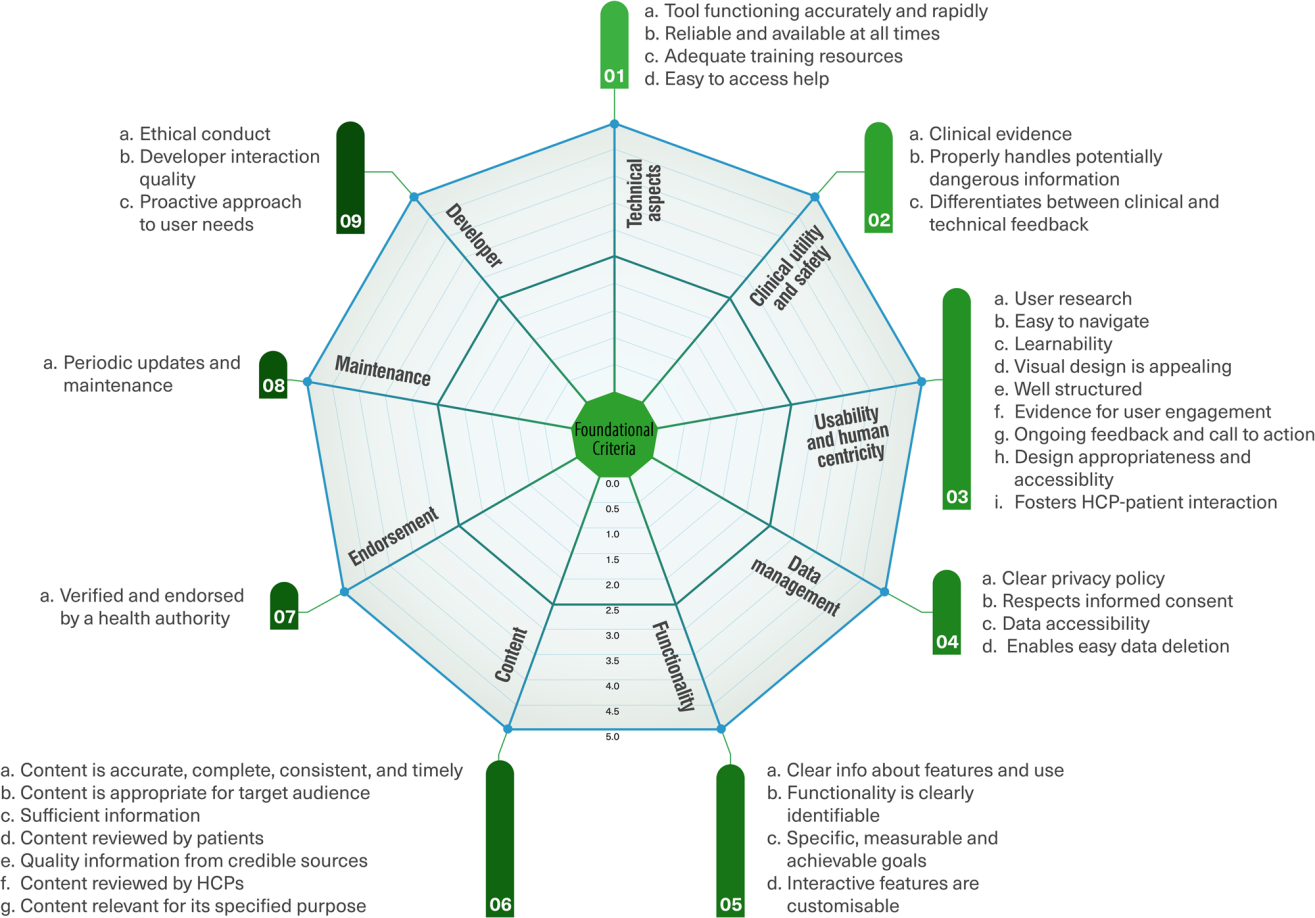
Foundational criteria clusters of the sociotechnical framework to assess patient-facing eHealth tools.

**Fig. 9:**
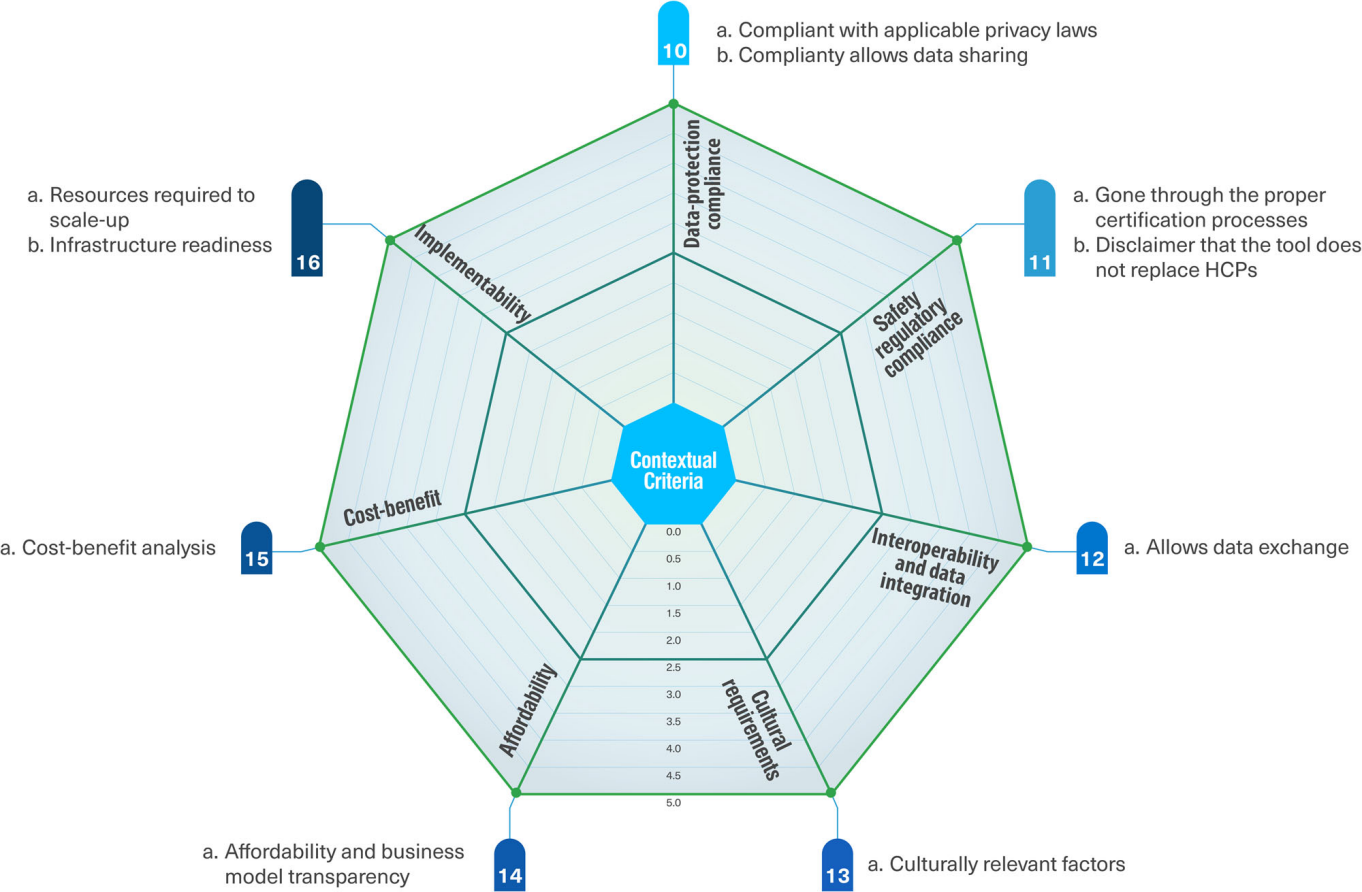
Contextual criteria clusters of the sociotechnical framework to assess patient-facing eHealth tools.

Considering the substantial diversity of eHealth tools, their use cases, integration level, and safety risk level, it is important to note that some assessment criteria may not apply to all tools. However, studies have shown that it can prove quite challenging to classify according to existing categorization due to the lack of standardization in this area^37^. We focused on safety risk categorization according to the NICE evidence standards framework^21^, given that safety is one of the key priorities when assessing these tools. Figure 8 shows the foundational criteria and Fig. 9 shows the contextual criteria of our proposed sociotechnical framework for assessing patient-facing eHealth tools according to our expert panel’s consensus.

The proposed assessment instrument with a full description of each assessment criterion, tangible examples of how to assess it, the risk tier it applies to, and additional guidance for assessors are available in Supplementary Reference 1 in the Supplementary Information file. Tables 2 and 3 summarize the definitions of the foundational and contextual criteria clusters respectively. The interactive assessment instrument will be available for download on the project website^38^. For each criterion, the assessor could follow a detailed description and examples to assess whether the criterion was met (5/5), partially met (2.5/5), or not met (0/5). For some criteria, the assessment options were binary, either met or not met. For instance, when assessing whether a tool has undergone proper certification processes, this criterion cannot be partially met. An option of (not applicable) was added for criteria that may be optional for some tools. For example, the criterion that assesses whether the tool has the ability to foster interaction between healthcare professionals and their patients may not apply for autonomous tools that were designed to be used independently. For criterion clusters that include more than one sub-criterion, the mean score is calculated to reflect the average assessment of this cluster. Mean scores are used in alignment with the familiar format of star ratings and similar assessment scales^39^; they are also more fit for purpose compared to total scores, because some criteria may be assessed as not applicable in specific cases.

**Table 2:** Definitions of the foundational criteria clusters.

Criteria Cluster	Definition
1- Technical aspects	Assesses whether the tool is functioning accurately and rapidly, is reliable and available at all times and can handle high levels of traffic and usage, provides adequate and user-friendly training resources for end users, and it is easy and obvious to access technical help when needed.
2- Clinical utility and safety	Assesses whether the tool’s clinical effectiveness is supported by strong research with adequate statistical power conducted by credible sources, warns about potential risks when necessary and properly handles potentially “dangerous” information entered by a patient, and differentiates between clinical and technical feedback, and clearly channels clinical feedback that may pose a health risk through the proper channels.
3- Usability and human centricity	Assesses whether the tool’s usability and acceptability has been rigorously trialed and tested in a real world setting, learning to use the tool is easy and does not require a lot of time, the visual design is appealing and has a harmonious look and feel, is well structured, and important information is clear and stands out, there’s evidence for co-creation and collaboration with users in the tool’s development, provides appropriate ongoing feedback and appropriate call to action based on the user’s state and activities (when applicable), its content and design are appropriate for the target audience and accessible to vulnerable populations, and has the ability to foster the interaction between the health care professionals and their patients (when applicable).
4- Data management	Assesses whether the tool has a clear privacy policy and informs the users on how their data will be kept confidential and secured and how the data may be used, respects informed consent and allows the user to opt out of data collection, its data can be accessed at any time and on different platforms and operating systems, and it explicitly and easily enables users to delete their data.
5- Functionality	Assesses whether there is clear information about the tool’s features and appropriate ways to utilize it, the functionality of each element is clearly identifiable, the tool has specific, measurable and achievable goals (desired outcomes) that are specified/obvious within the tool itself, and interactive features such as reminders, push notifications, and prompts are customizable and not overwhelming.
6- Content	Assesses whether health-related content is accurate, complete, consistent, and timely; is provided in a clear and appropriate way for the target audience; there is sufficient information throughout the tool without any omissions, over-explanations, or irrelevant data; the content has been reviewed by patients to ensure readability and acceptability; the tool contains high quality information from credible and legitimate sources; has been reviewed by (or originated from) healthcare professionals with the most updated evidence-based practice of medicine, and contents are relevant to the underlying objective and likely to be effective in achieving the specified purpose in the specific intended population.
7- Endorsement	Assesses whether the tool has been verified, given a good review, or endorsed by a legitimate/reliable source such as a health organization, health authority, scientific/medical society (e.g., APA; FDA in the US; NIH; NHS in the UK; NICE in the UK) or recommended by trusted Healthcare Professionals.
8- Maintenance	Assesses whether the tool gets periodic updates and maintenance both from technical and content perspectives (e.g., last update not older than xx months depending on the use case, the content is periodically updated with the new findings in the medical field).
9- Developer	Assesses whether the tool’s provider respects ethical conduct, clinical responsibility, and the rules and regulations protecting patient’s rights and societal interests; interaction quality between the tool’s provider and the users, including responsiveness, after sales services, and customer orientation is high; and the tool’s provider demonstrates a proactive approach to the assessment of user needs, and continuous learning.

**Table 3:** Definitions of the contextual criteria clusters.

Criteria Cluster	Definition
10- Data-protection compliance	Assesses whether the tool explicitly reports being compliant with the relevant data privacy and protection laws (e.g., GDPR, HIPAA…), and the treatment of any personal data is compatible with the Patient Data Act, Personal Data Act, and other applicable privacy laws, and compliantly allows for data sharing and segregation for research use.
11- Safety regulatory compliance	Assesses whether the tool’s provider clearly identifies the risks that its management may pose for user safety and has gone through the proper certification processes to ensure its safety; and the tool contains a disclaimer that the information provided/content does not replace a health care professional’s judgment (when applicable).
12- Interoperability and data integration	Assesses whether the tool allows for interoperability, data integration and exchange of data with other apps, e-tools, wearable devices, electronic health records (ability to exchange data with other systems on a technical and policy level, and with other users such as clinicians or care givers).
13- Cultural requirements	Assesses whether the tool takes into account culturally relevant factors (e.g., different languages and alphabets, specific religious or cultural requirements or restrictions, gender considerations).
14- Affordability	Assesses whether the tool is affordable taking into account the local socioeconomic context, and whether it is clear who pays for it and how they pay.
15- Cost-benefit	Assesses whether a cost-benefit analysis was performed and led to positive results. I.e., the balance between the costs and benefits arising from the tool’s utilization. This refers to the tool’s direct costs (purchase price, subscription, licensing…), but may also include costs associated with the tool’s selection, staff training, setting up support mechanisms, and appropriate governance.
16- Implementatability	Assesses whether the tool fits well into existing workflows and does not require additional resources (workforce, hardware, software) to scale-up and to enable it to function properly; and whether it fits well into the existing infrastructure and does not require investment in additional infrastructure to enable it to function properly (This refers to physical infrastructure such as electricity, access to power, connectivity etc. in the local context).

When relevant, assessors are given additional resources to support their evaluation by providing more details about quality standards for this specific criterion. For instance, clinical evidence is one of the criteria in the foundational cluster (clinical utility and safety), which may require deeper examination by employing additional standards tailored for that specific area. In this example, the guidance includes more details on the checklist of the evidence quality criteria of the evidence DEFINED (Digital Health for EFfectiveness of INterventions with Evaluative Depth) framework^40^. Such additional resources aim to help the assessor gain more insights into how to assess the quality of the criterion, particularly in cases where they may not have enough experience in this specific domain.

We methodically reflected on the considerable challenges facing eHealth assessment efforts that we identified in our foundational work^18^. While some of them were beyond our control (e.g., information availability and regulatory complexity), we focused our efforts on attempting to address some of these key challenges, namely assessment criteria validation with intended users and concerned parties to reflect their real-life needs, ensuring assessor diversity in our expert panel to reflect a wide breadth of perspectives, considering healthcare contextuality, discussing ways to address subjective measures, and practicability of the proposed assessment instrument to make it as accessible and usable as possible.

The expert panel helped us validate the assessment criteria, and participants’ diversity was instrumental in reflecting the different priorities and perspectives of all relevant concerned parties. The initial list of criteria included contextual criteria that the experts validated and confirmed as must-have in the final framework. Experts were also asked to suggest additional assessment criteria that they deemed important. These new criteria were validated in the second-round survey to ensure the completeness of the final list of criteria. Our discussions with the experts went beyond finalizing the list of assessment criteria to encompass the practicability and relevance of the proposed assessment instrument to make it as accessible and usable as possible to relevant decision makers.

The first challenge we discussed with the experts was the contextuality of some of the assessment criteria. Research has shown that contextual factors that go beyond the tool being used itself, such as implementation costs, clinical workflows, required resources and infrastructure, and a patient’s characteristics and socioeconomic status, typically play a major role in eHealth acceptance and adoption^41–46^. This contextual nature of healthcare makes engagement with eHealth tools significantly challenging when contextual awareness is not considered^47^. However, although there are some standards for assessing a tool’s usability or scientific evidence, there seems to be a gap in guidelines supporting the evaluation of their implementation and processes^8^, resulting in a gap in contextual assessment criteria.

While numerous assessment initiatives and frameworks solely focus on assessing the tool itself and do not take the healthcare context into account, we advocate for the inclusion of contextual criteria (e.g., readiness of the local infrastructure, required resources for scale-up, cost-benefit analysis, reimbursement standards, and cultural aspects like local language), as these have proven to greatly impact the adoption and scale-up of such tools^3,41,43^. The overwhelming expert consensus on the inclusion of contextual criteria (55/55, 100%) confirmed their importance and relevance for a comprehensive assessment.

The second challenge was the use of a single score versus a scorecard for the assessment results. Some assessment initiatives that focus on curating, certifying, or accrediting eHealth tools attempt to use a single-score approach to signify quality, with the goal of making it easy for potential customers to compare tools and differentiate between low- and high-quality offerings. While these initiatives certainly have merits in advancing assessment efforts, scholars argue that they may not provide an adequately clear direction on the most effective tools that meet certain requirements to best integrate into a specific healthcare context^13,16^. Hence, a scorecard approach may be more suitable for context-specific evaluations that involve multiple concerned parties^13^.

Expert consensus confirmed that the scorecard was a more balanced way of presenting the assessment results, with (43/55, 78%) of the experts favoring this approach. Experts have argued that a single score may obscure important details, and that the real value of the assessment instrument is about understanding the breakdown. This breakdown can be useful in understanding the specific strengths and weaknesses of the tool being assessed. Some experts (12/55, 22%) suggested that, ideally, the assessment results would be presented as a combination of a scorecard and a composite score that gives more weight to the assessment criteria that the assessor defines as a key priority for their specific context to make comparability easier if the assessor is comparing several tools at the same time.

The third challenge we discussed was about balancing the assessment automation with AI versus a proactive appraisal approach that requires testing the tool. The latest advancements in Artificial Intelligence (AI) have enabled several appraisal initiatives to use sophisticated AI models to evaluate eHealth tools by scanning publicly available information to form a basic quality assessment. Even though this approach may enable the assessment of large numbers of tools in a relatively short time, one of the risks of this mass appraisal approach is that it may favor tools with sophisticated marketing efforts and a polished public image, which may not necessarily offer superior clinical utility. Another challenge is lack of information^18,48^. A previous study showed that about two-thirds of eHealth providers provided no information about the tool itself, nor about the credentials of developers or consultants, and only 4% provided information supporting its efficacy^49^.

Therefore, we propose a proactive approach to appraisal that requires hands-on trial of the tool and getting in touch with the developer, if necessary, to have a more complete and in-depth assessment of the quality of the tool. This approach entails more engagement and effort from the assessors’ side but would result in more in-depth insights into the specific strengths and weaknesses of the tool being evaluated, leading to better and more informed decisions. The need for testing and hands-on trials to properly assess an eHealth tool has been recommended by other researchers^16,48^; some have even recommended using a tool for a period of at least two hours across more than one day before reaching a final evaluation^11^. Previous studies have also suggested criteria and questions that physicians may directly ask eHealth providers to gain a complete understanding of the tool before adopting or endorsing it to their patients^5^.

A small number of experts (4/55, 7%) disfavored the proactive approach, arguing that it required too much effort and time, which may discourage assessors from using the proposed assessment instrument. However, most experts (36/55, 65%) favored it, foreseeing that a proper and in-depth assessment certainly requires hands-on trial of the tool being assessed and getting in touch with the tool developers if needed. Some experts (10/55, 18%) confirmed the necessity of a proactive approach and suggested a mixed method in which an AI model collects the available appraisal data that does not require trying the tool, complemented by the assessor’s hands-on assessment of the criteria that necessitate it.

The fourth challenge was about the subjectivity of some of the assessment criteria. The inclusion of subjective measures has been debated in the literature as it may cause variability in the assessment outcome depending on the assessors’ subjective views. This was confirmed by previous research, which showed that some characteristics of eHealth tools are indeed more difficult to rate consistently^11^. Low rating agreements by some of the widely known assessment initiatives, such as ORCHA, MindTools, and One Mind Psyber Guide, are not uncommon^50^. Several scholars still strongly recommend the inclusion of subjective criteria, such as ease of use and visual appeal, despite this challenge given their importance as fundamental adoption drivers^41–43,51,52^. Hence, integrating subjective criteria, such as user experience evaluation, into the review process could improve tool adherence and health outcomes^34^. Expert consensus clearly confirmed this view, with multiple subjective criteria meeting the predefined consensus level.

The experts recommended a few approaches that may help minimize the assessment variability of the subjective criteria. The most prominent recommendation was to ensure assessor diversity (34/55, 62%), thereby contributing to a balanced assessment that reflected their different views and priorities. This is an important factor, as it has been previously reported in the literature that some assessment initiatives do not necessarily involve all relevant concerned parties in the evaluation^11,28^. Assessor diversity is also crucial to ensure balancing of conflicting needs of the different concerned parties, which has been highlighted in previous research that showed that sometimes patient needs may not always be aligned with clinicians’ workflow preferences^53^. Evidence of user engagement and co-creation with patients and clinicians is another way of balancing the perspectives of the different concerned parties in the design^54^, a criterion that clearly met the expert consensus (81%).

Research evidence, such as usability studies, was also recommended (20/55, 36%) to judge usability and acceptability. It is important to note that not all usability studies have equal rigor and quality. Assessors are advised to take a closer look at such studies to inspect factors such as sample size, diversity, and rigor of the study methodology. The clarity and specificity of assessor guidance to reach a common understanding of how to assess these criteria (16/55, 29%) was also recommended to minimize the subjectivity of the assessment.

Using proxy criteria such as customer ratings was suggested as a workaround to get an idea of a tool’s acceptance when rigorous user research is not available (9/55, 16%), with the caveat that a critical mass must be achieved for the user rating to be considered. Even though user rating may be considered one of the decisive factors for prospective users when deciding whether to use a tool, this criterion has been disputed not only in the expert panel but also in the literature^34^. While there are studies that show a moderate correlation between user ratings and expert appraisal^39^, others have reported a weak correlation between the two^11^. This lack of agreement was also reflected in the expert consensus on the criterion “visible users’ reviews” that did not meet expert consensus (44%) and is considered a nice-to-have criterion in the proposed assessment framework. Another proxy criterion suggested by experts was the use of a tool’s usage metrics as an objective indication of its usability (4/55, 7%).

The fifth challenge was whether to enable the optionality of some of the criteria. Some criteria clearly met expert consensus but were still debated in the survey comments as to whether they were applicable to all types of tools. For example, the criterion that assesses whether the tool has the ability to foster interaction between healthcare professionals and their patients met an 86% consensus. However, some experts argued that it may not apply to autonomous tools that were designed to be used independently (e.g., for some mental health tools, the interaction with the traditional healthcare system is sometimes not even desired for anonymity and privacy reasons).

The majority of experts (39/55, 71%) acknowledged the vast variability of eHealth tools, which compels the optionality of some criteria, even though they met the expert consensus. This is aligned with other assessment initiatives and rating systems that also include an option of “not applicable” for criteria that may be optional for some tools^48^.

The sixth challenge was about the use of current versus progressive criteria. Some criteria were disputed for being perceived as shortsighted or for hindering innovation. For instance, the safety criterion that assesses whether the tool contains a disclaimer that the information provided does not replace a health care professional’s judgement met 81% consensus for risk tiers B and C. However, three experts favored a more progressive assessment framework that dismissed such a criterion, given all the AI-driven medicines that we are witnessing today. This progressive stance may have emerged from the ‘fail fast, fail often’ culture of technology startups that often clashes with the complex healthcare regulations, that typically result in a more cautious and slow process, characterized by more risk aversion guided by the ‘first, do no harm’ principle^16,55^.

This tension in balancing safety and innovation was acknowledged by the Food and Drug Administration Commissioner when he admitted that eHealth tools are developing faster than the FDA is able to regulate them^56^. The vast majority of experts favored the current criteria (50/55, 91%), mostly to safeguard patient safety. Most experts acknowledged that assessment criteria are tied to the current healthcare context, especially to how progressive the regulations and legislation are. This means that it is not the criteria itself that would be prohibitive, but it is more the regulatory landscape and the legislation that would be prohibitive in this case, so the criteria are sorted according to the maturity of the overall landscape. Accordingly, the assessment framework and the respective list of criteria are likely to progress over time to reflect the changing technologies and regulations.

This study has some limitations. The Delphi process has some inherent limitations, such as the relatively small number of experts and the potential impact of panel configuration on the findings^57,58^. Furthermore, the proposed framework and assessment instrument require time and expertise, which may be barriers to the engagement of some of the concerned parties in the evaluation process. Detailed training materials and assessment guidance may help to mitigate this barrier. Future research is required to pilot test the proposed assessment instrument and determine its accessibility and usability across different concerned parties, as well as its adaptability for the tools under development (e.g., as a requirements checklist for developers). Future refinements of the assessment criteria, their definitions, and additional criteria will likely be required as new technologies and eHealth regulations evolve.

This sociotechnical assessment framework goes beyond the assessment of isolated technologies and considers their intended context. It helps us answer two key questions: “what is the assessment of the quality of the tool itself” and “what is the assessment of its potential impact in a specific healthcare setting.” The international expert panel helped us validate the assessment criteria, and participants’ diversity was instrumental in reflecting the different priorities and perspectives of all relevant concerned parties. The proposed assessment instrument will inform the development of a specific assessment process for a single tool. It will also enable side-by-side comparisons of two or more tools which are intended for the same purpose. However, it does not determine which criteria at which score level are essentially go or no-go criteria, as this will have to be determined by those applying the instrument based on the context of use and available alternatives. Broadly, it will help inform a wide range of concerned parties, including clinicians, pharmaceutical executives, insurance professionals, investors, technology providers, and policymakers about which criteria to assess when considering an eHealth tool. This can guide them in making informed decisions about which tools to use, endorse to patients, invest in, partner with, or reimburse based on their potential quality and their fit into the specific context for which they are being evaluated.

## Methods

### Design of the modified Delphi process

**Fig. 10:**
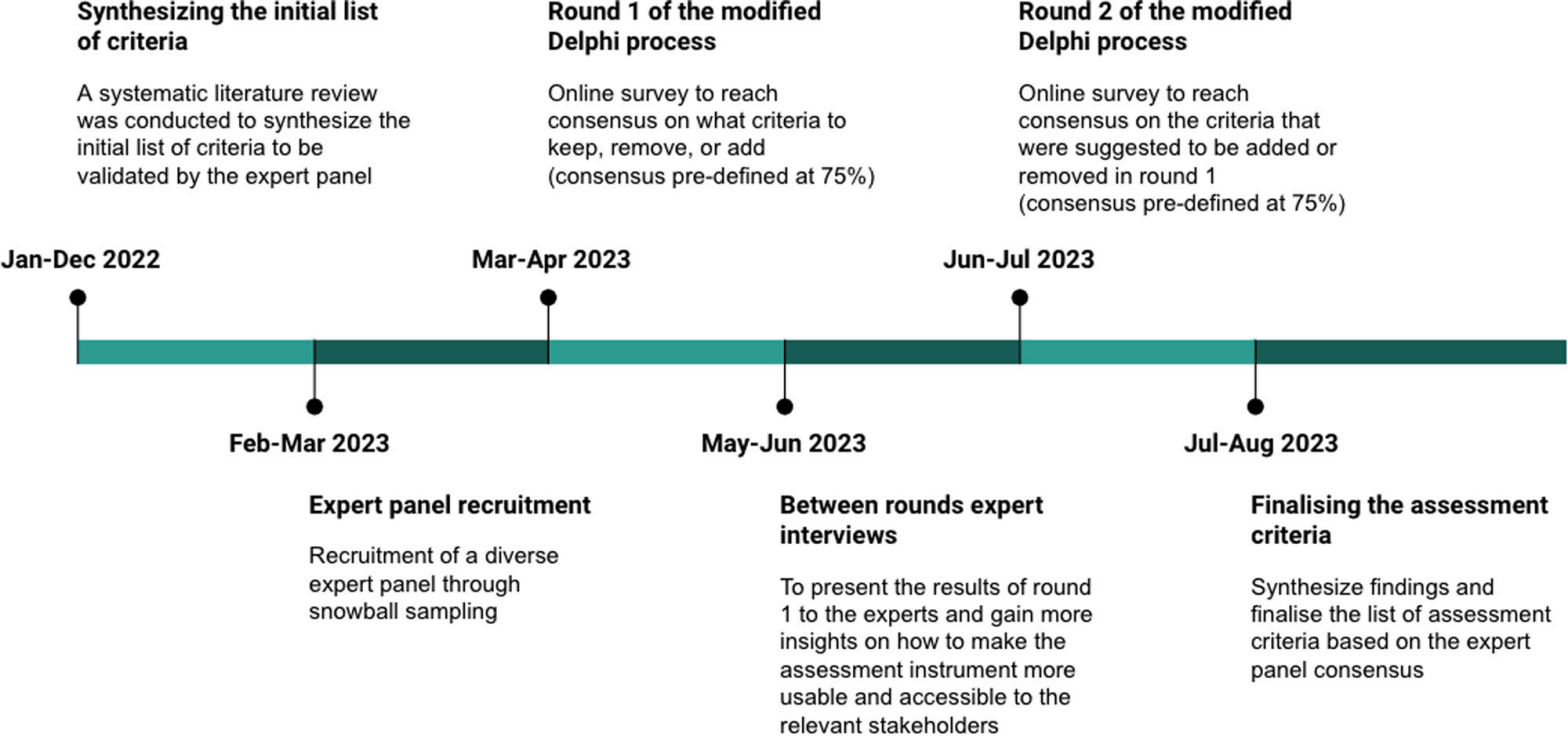
Design of the modified Delphi process.

The Delphi method was established in the 1950s by Norman Dalkey and Olaf Helmer in an effort to gain reliable expert consensus^59^. Its main purpose is to facilitate and gather a consensus of expert opinions in the face of complex problems^60^. This technique has gained acceptance in diverse fields of medicine^61^, especially as a method for the development of best practice guidance and clinical guidelines^62^. However, the Delphi processes used in previous healthcare-related studies have not always been homogenous, with clear variations in study design, conduct, and reporting^62^. Therefore, we have been guided in our study design by the quality recommendations from the Guidance on Conducting and REporting DElphi Studies (CREDES)^62^ and the methodological quality map suggested by Niederberger et al.^63^. These guidelines helped us to thoroughly reflect on every step of our modified Delphi process and to base our methodological decisions on best practices. Figure 10 shows the steps followed in our modified Delphi process, and the following sections describe each step in detail.

### Synthesizing the initial set of assessment criteria

Previous methodological guidance and best practice frameworks have suggested that the quality of information provided to the panelists, such as synthesis of the available evidence, may influence and possibly bias their judgement if not done objectively^60,62^. Therefore, to minimize bias and avoid an initial set of criteria that was impacted by the subjective opinions of the research team, the authors conducted a systematic literature review to identify the relevant criteria used to assess patient-facing eHealth tools in a rigorous manner that builds on previous efforts^18^. The systematic literature review searched the PubMed, Cochrane, Web of Science, Scopus, and ProQuest databases for studies published between January 2012 and January 2022 in English, yielding 675 results, of which 40 studies met the inclusion criteria. The Preferred Reporting Items for Systematic Reviews and Meta-Analyses (PRISMA) guidelines^64^ and Cochrane Handbook^65^ were followed to ensure a systematic process. Extracted data were analyzed using NVivo (QSR International), with thematic analysis and narrative synthesis of emergent themes^66–68^.

Similar assessment criteria from the different papers, frameworks, and initiatives were aggregated into 36 unique criteria grouped in 13 clusters, guided by the sociotechnical theory, which has at its core the idea that the design and performance of any innovation can only be understood and improved if both ‘social’ and ‘technical’ aspects are brought together and treated as interdependent parts of a complex system^36^. Consequently, the resulting criteria were classified into technical, social, and organizational criteria. Technical assessment criteria were grouped into five clusters: technical aspects, functionality, content, data management, and design. Social assessment criteria were grouped into four clusters: human centricity, health outcomes, visible popularity metrics, and social aspects. The organizational assessment criteria were grouped into four clusters: sustainability and scalability, healthcare organization, healthcare context, and developer.

### Participant recruitment

According to CREDES^62^, the most prominent criteria for the identification and selection of experts in Delphi studies are the representation of a particular profession or concerned party, affiliation to a particular setting or work field, relevant clinical or academic expertise, membership in an organization or professional board, or being a recognized authority in the field. Hence, the concerned parties deemed relevant for this research were defined as individuals from the following groups, all of whom were required to have in-depth eHealth expertise: clinicians, patients and patient advocates, researchers, pharmaceutical executives, insurance and reimbursement experts, compliance experts, investors and funding experts, and medical technology providers. We define an eHealth expert as someone who has practical hands-on expertise in co-creating, implementing, assessing, and/or commercializing eHealth tools. The choice of concerned parties reflects the composition of the relevant players in the healthcare ecosystem and is aligned with previous studies that had similar objectives and used similar sampling methods^69,70^.

Considering the specific expertise required for this research, purposive sampling was used, in which potential participants were selected based on their ability to provide rich and in-depth information about the topic^66,71^. The Internet and social media sites offer many opportunities for participant recruitment^66^, especially for researchers with long experience in the field, allowing them to build a wide and strong network of relevant experts. The first author, C.J., used her well established eHealth network to shortlist the participants of interest as per the criteria explained earlier in this section (practical hands-on expertise in co-creating, implementing, assessing, and/or commercializing eHealth tools and belongs to one of the key concerned parties), and started to contact them via the professional social media site LinkedIn. She chose this platform because it transparently informs the potential participant about her professional experience, background and it also allows them to check others’ endorsement of the researcher. She then used snowball sampling to minimize potential selection bias and expand the sample through the network of other participants^66^, in alignment with previous studies that had similar objectives and used similar sampling methods^69,70^.

In this expert-driven sampling process, we tried to the best of our ability, according to best practice recommendations^61–63^, to ensure diversity among the experts in terms of gender, location, professional background, and the concerned party to which they belong. Even though there is no standard size for panel members, previous research has shown that a double-digit number of around 30–50 is considered optimum in concluding rounds for a homogenous Delphi process^61,63^. Accordingly, the research team selected a target sample size of approximately 40 experts. A total of 120 experts were contacted and 57 (47.5%) agreed to participate in the study. Participation was voluntary and the experts were not compensated for their contribution.

### Round 1 of the modified Delphi process

Experts who agreed to participate and signed a consent form received an email inviting them to participate in the first round of the modified Delphi process via an online survey. The survey was created using the online survey tool Survey Monkey, and it lasted from March 1st to April 15th 2023.

Each criterion was surveyed according to its relevance using a 5-point Likert scale with extremes labeled (1, “I suggest this criterion is excluded”; 5, “This criterion is extremely relevant”). While consensus levels in previous Delphi studies vary widely from 50% to 100%^59,61^, a threshold of 60% or higher seems to be adopted in most cases^63^, with a cut-off of 75% or 80% in the majority of studies^62^. Therefore, following the best practice recommendations, we set the consensus level a priori to 75%^61,62^. A criterion was accepted if at least 75% of the experts rated it as 4 or 5. For each criterion, the mean, standard deviation, median, and interquartile range of the ratings were calculated to show data variability and distribution.

The survey also included an open-ended blank space at the end of each dimension, in which experts could post comments, provide additional information (e.g., propose new criteria), and make clarifications. Participants were also asked about criteria classification according to a tool’s risk category, as per the NICE evidence standards framework^21^. Tier A tools are those which have no direct outcome on the patient, but which are intended to save costs or staff time (e.g., electronic prescribing systems that do not provide advice to patients, complex scheduling software); tier B tools are those that assist the public to manage their own health (e.g., instant messaging apps for healthcare, symptom or mood diaries and programs to aid weight-loss or better sleep); and tier C tools are those used for treating and diagnosing medical conditions, with direct health outcomes, and which are likely to be regulated medical devices (e.g., symptom monitors which share data with care teams, triaging systems that use patient health data to assist with care decisions and devices that perform diagnostic image analysis for making treatment decisions)^21^. They had to define whether a criterion was valid for: (1) all risk categories (A, B, and C), (2) only medium to high risk—tiers B (understanding and communicating) and C (interventions); and (3) only high risk—tier C (interventions). Supplementary Reference 2 in the Supplementary Information file provides a copy of the full survey.

While gender, geographic, and expert profile diversity were considered in the sample recruitment, we did not ask questions about these characteristics as part of the survey questions to reduce the participants’ burden and preserve the anonymity of responses, an important feature of the Delphi process^63,69^. Statistical analysis was performed by R.M. using the R project for statistical computing^72,73^, and C.J. analyzed the qualitative input received from open-ended questions using NVivo (QSR International), a computer-assisted qualitative data analysis software. C.J. performed the initial analysis and coding, the second author J.L. reviewed the coding, and any cases of disagreement were discussed and mutually agreed upon in conjunction with one of the last two authors (S.H. and M.P.).

### Between rounds expert interviews

After analyzing the results of the first round, each expert was invited to a one-to-one semi-structured interview with C.J., with the goal of presenting the results of the first round and gaining more insights on how to make the proposed assessment instrument more usable and accessible to the relevant concerned parties. Individual interviews were chosen as opposed to focus groups or workshops to avoid any bias that might result from experts being worried about their own views being viewed negatively by other members of the panel^59^, to avoid common socially induced bias^60^, and to conform to a dominant view^61,62^.

Narrative synthesis was used to analyze the interview transcripts, using NVivo (QSR International), a computer-assisted qualitative data analysis software. Data coding began with a preliminary data extraction grid that included themes based on the expert comments received in the Round 1 survey and informed by our previous work that aggregated the factors impacting adoption from patients’ and clinicians’ perspectives, as well as the initial list of criteria for eHealth assessment^18,41–43^. More codes were added as they emerged during the analytical process. Braun and Clarke’s thematic analysis^66–68^ was used to identify and extract the themes addressed in the research question. The first author C.J. conducted the interviews and performed initial analysis and coding. The second author J.L. reviewed the coding, and any cases of disagreement were discussed and mutually agreed upon in conjunction with one of the last two authors (S.H. and M.P.). This process lasted from May 15th to July 15th 2023.

### Round 2 of the modified Delphi process

The criteria that did not reach the set agreement level in round 1, as well as the new criteria suggested by the participating experts, were included in the second round of the modified Delphi process. A new survey was designed to determine the validity of the criteria. The second survey was also created using the online survey tool Survey Monkey and lasted from May 15th to July 15th 2023. Supplementary Reference 3 in the Supplementary Information file provides a copy of the full survey.

The criteria that did not meet the consensus in round 1 were presented, and experts were requested to provide a value for the criterion (using the same 5-point Likert scale as in the first round: 1, “I suggest this criterion is excluded”; 5, “This criterion is extremely relevant”). A criterion was accepted if at least 75% rated it as either 4 or 5. For each criterion, the mean, standard deviation, median, and interquartile range of the ratings were calculated to show data variability and distribution. Participants were not asked to reassess the criteria classification according to the tool’s risk category, as this information was already collected in the first round.

For the additional criteria that were suggested to be added by the experts in round 1, participants were also requested to provide a value for the criterion (using the same 5-point Likert scale as in the first round: 1, “I suggest this criterion is excluded”; 5, “This criterion is extremely relevant”). A criterion was accepted if at least 75% rated it as either 4 or 5. For each criterion, the mean, standard deviation, median, and interquartile range of the ratings were calculated to show data variability and distribution. Participants were also asked about criteria classification according to a tool’s risk category, as per the NICE evidence standards framework^21^. They had to define whether a criterion was valid for: (1) all risk categories (A, B, and C), (2) only medium to high risk—tiers B (understanding and communicating) and C (interventions); and (3) only high risk—tier C (interventions).

The survey also included an open textual response option at the end of each dimension, in which experts could post comments, provide additional information, and make clarifications. Statistical analysis was performed by (R.M.) using the R project for statistical computing^72,73^. And (C.J.) analyzed the qualitative input received from open-ended questions using NVivo (QSR International), a computer-assisted qualitative data analysis software. (C.J.) performed the initial analysis and coding, the second author (J.L.) reviewed the coding, and any cases of disagreement were discussed and mutually agreed upon in conjunction with one of the last two authors (S.H.) and (M.P.).

### Finalizing the assessment criteria

After the second-round survey was completed by the expert panel, the final list of criteria was compiled to reflect:1) criteria that met consensus; 2) criteria that did not meet the consensus; and 3) criteria that were suggested to be added by the experts in the first-round survey and met the consensus level in the second-round survey (rated 4 or 5 by at least 75% of the experts).

### Ethical considerations

The Ethics Committee of Northwest and Central Switzerland (EKNZ) determined that ethical approval was not needed for this study, according to the Federal Act on Research involving Human Beings, Article 2, paragraph 1 (reference number Req-2022-01499). Participants signed an informed consent form and returned it to the lead author electronically via e-mail. Information collected from participating experts was anonymized and securely held. Personal identifiable information (e.g., consent forms) was kept separately, and participants were assigned a study code number and identifying information was stored separately from the data.

### Supplementary information

Supplementary File

## Data Availability

C.J. has full access to all of the data in the study and takes responsibility for the integrity of the data and the accuracy of the data analysis. All study materials are available from the corresponding author upon reasonable request.
