# Supplementary material for: A sociotechnical framework to assess patient-facing eHealth tools: results of a modified Delphi process
Source: NPJ Digit Med. 2023 Dec 15;6:232. doi: 10.1038/s41746-023-00982-w (PMC10724255; doi:10.1038/s41746-023-00982-w)
Supplement: Supplementary file 1 — Supplementary File [file 41746_2023_982_MOESM1_ESM.pdf]

Supplementary Table 1: Round 1 survey results (2 pages)

Assessment Criteria - short version	Assessment Criteria - complete version	1 I suggest this criterion is excluded		2		3		4		5 This criterion is extremely relevant		I can't assess		CONSENSUS	MEAN	STDEV	MEDIAN	IQR	Q1	Q3	all risk tiers (A, B, and C)		only medium to high risk – tiers B (understanding/		only high risk – tier C (interventions)		I am not sure	
1.1.1. Tool functioning accurately and rapidly	1.1.1. The tool is functioning accurately and rapidly, without any error messages, glitches, or crashes (e.g., unexpected stops of running, response time)	0,00%	0	1,75%	1	5,26%	3	24,56%	14	68,42%	39	0,00%	0	92,98%	4,6	0,7	5	1	4	5	75,44%	43	15,79%	9	7,02%	4	1,75%	1
1.1.2. Adequate training resources	1.1.2. The tool provides adequate training resources for end users to ensure their comfort with basic competencies and skills needed to use the tool effectively (e.g., in the form of training material, tutorials, videos, user guides or documentation)	0,00%	0	1,75%	1	5,26%	3	56,14%	32	36,84%	21	0,00%	0	92,98%	4,3	0,6	4	1	4	5	59,65%	34	28,07%	16	12,28%	7	0,00%	0
1.1.3. Easy to access help	1.1.3. It's easy and obvious to access help when needed (e.g. hotline, support email, contact form, help section)	0,00%	0	0,00%	0	14,04%	8	31,58%	18	54,39%	31	0,00%	0	85,97%	4,4	0,7	5	1	4	5	54,39%	31	29,82%	17	10,53%	6	5,26%	3
1.1.4. Possibility to give instant feedback	1.1.4. The tool allows the possibility to give instant feedback to the developers (e.g., provider messaging to report technical issues or errors)	5,26%	3	12,28%	7	17,54%	10	43,86%	25	21,05%	12	0,00%	0	64,91%	3,6	1,1	4	1	3	4	54,39%	31	22,81%	13	14,04%	8	8,77%	5
1.2.1.a. Clear info about features and use	1.2.1.a. There is clear information about the tool's features and appropriate ways to utilize it (e.g., adjunct, standalone)	1,75%	1	1,75%	1	10,53%	6	36,84%	21	49,12%	28	0,00%	0	85,96%	4,3	0,9	4	1	4	5	66,67%	38	28,07%	16	3,51%	2	1,75%	1
1.2.1.b. Functionality is clearly identifiable	1.2.1.b. The functionality of each element is clearly identifiable (e.g. if the user must take a specific action, the tool clearly and visually indicates the action to be taken)	1,75%	1	1,75%	1	0,00%	0	26,32%	15	70,18%	40	0,00%	0	96,50%	4,6	0,8	5	1	4	5	66,67%	38	22,81%	13	8,77%	5	1,75%	1
1.2.2.a. Specific, measurable and achievable goals	1.2.2.a. The tool has specific, measurable and achievable goals (desired outcomes) that are specified/obvious within the tool itself	0,00%	0	0,00%	0	10,53%	6	31,58%	18	57,89%	33	0,00%	0	89,47%	4,5	0,7	5	1	4	5	50,88%	29	33,33%	19	10,53%	6	5,26%	3
1.2.2.b. Considers related health issues	1.2.2.b. The tool considers multiple health issues and related ones, and sufficiently addresses them to help meet the intended purpose	5,26%	3	3,51%	2	24,56%	14	29,82%	17	29,82%	17	7,02%	4	59,64%	3,8	1,1	4	2	3	5	26,32%	15	33,33%	19	21,05%	12	19,30%	11
1.2.3. Interactive features are customisable	1.2.3. Interactive features such as reminders, push notifications, and prompts are customisable and not overwhelming (e.g. users can customise the frequency and timing of reminders to suit their daily routines)	3,51%	2	1,75%	1	5,26%	3	28,07%	16	61,40%	35	0,00%	0	89,47%	4,4	0,9	5	1	4	5	59,65%	34	33,33%	19	5,26%	3	1,75%	1
1.3.1.a. Content is accurate, complete, consistent, a	1.3.1.a. Health-related content is accurate, complete, consistent, and timely (e.g. according to state of the art scientific evidence)	0,00%	0	1,75%	1	1,75%	1	12,28%	7	84,21%	48	0,00%	0	96,49%	4,8	0,6	5	0	5	5	64,91%	37	28,07%	16	7,02%	4	0,00%	0
1.3.1.b. Content is appropriate for target audience	1.3.1.b. The tool's content is provided in a clear and appropriate way for the target audience (using an understandable, plain and simple language, with messages adapted to the user profile in terms of linguistic style and level, facilitating user understanding and avoiding using technicalities)	0,00%	0	1,75%	1	5,26%	3	21,05%	12	71,93%	41	0,00%	0	92,98%	4,6	0,7	5	1	4	5	61,40%	35	33,33%	19	5,26%	3	0,00%	0
1.3.1.c. Sufficient information	1.3.1.c. There is sufficient information throughout the tool without any omissions, over-explanations, or irrelevant data	3,51%	2	5,26%	3	17,54%	10	36,84%	21	35,09%	20	1,75%	1	71,93%	4,0	1,0	4	2	3	5	50,88%	29	33,33%	19	7,02%	4	8,77%	5
1.3.2.a. Quality information from credible sources	1.3.2.a. The tool contains high quality information (e.g. text, feedback, charts, measures, references) from credible and legitimate sources (e.g., WHO)	1,75%	1	1,75%	1	12,28%	7	24,56%	14	59,65%	34	0,00%	0	84,21%	4,4	0,9	5	1	4	5	38,60%	22	45,61%	26	8,77%	5	7,02%	4
1.3.2.b. Content reviewed by HCPs	1.3.2.b. The content has been reviewed by (or originated from) healthcare professionals	1,75%	1	1,75%	1	5,26%	3	21,05%	12	70,18%	40	0,00%	0	91,23%	4,6	0,8	5	1	4	5	49,12%	28	36,84%	21	10,53%	6	3,51%	2
1.3.3. Content relevant for its specified purpose	1.3.3. The tool's contents are relevant to the underlying objective and likely to be effective in achieving the specified purpose in the specific intended population	0,00%	0	1,75%	1	7,02%	4	21,05%	12	70,18%	40	0,00%	0	91,23%	4,6	0,7	5	1	4	5	45,61%	26	36,84%	21	12,28%	7	5,26%	3
1.4.1.a. Clear privacy policy	1.4.1.a. The tool has a clear privacy policy and informs the users on how their data will be kept confidential and secured and how the data may be used (e.g., for commercial or research purposes)	0,00%	0	0,00%	0	3,51%	2	14,04%	8	82,46%	47	0,00%	0	96,50%	4,8	0,5	5	0	5	5	87,72%	50	8,77%	5	1,75%	1	1,75%	1
1.4.1.b. Compliant with applicable privacy laws	1.4.1.b. The tool explicitly reports being compliant with the relevant data privacy and protection laws (e.g. GDPR, HIPAA...), and the treatment of any personal data is compatible with the Patient Data Act, Personal Data Act, and other applicable privacy laws	0,00%	0	1,75%	1	5,26%	3	12,28%	7	80,70%	46	0,00%	0	92,98%	4,7	0,6	5	0	5	5	87,72%	50	10,53%	6	1,75%	1	0,00%	0
1.4.1.c.Respects informed consent	1.4.1.c. The tool respects informed consent and allows the user to opt out of data collection (e.g. ability to configure the settings of their data storage, access, and management)	0,00%	0	1,75%	1	3,51%	2	14,04%	8	78,95%	45	1,75%	1	92,99%	4,7	0,6	5	0	5	5	73,68%	42	17,54%	10	5,26%	3	3,51%	2
1.4.2.a. Allows data exchange	1.4.2.a. The tool allows exchange of data with other apps, e-tools, wearable devices, electronic health records (ability to exchange data with other systems on a technical and policy level)	1,75%	1	5,26%	3	8,77%	5	26,32%	15	56,14%	32	1,75%	1	82,46%	4,3	1,0	5	1	4	5	64,91%	37	19,30%	11	5,26%	3	10,53%	6
1.4.2.b. Allows different platforms	1.4.2.b. The tool allows the user to move across different platforms (e.g. mobile app vs web app, iOS vs Android)	3,51%	2	3,51%	2	21,05%	12	29,82%	17	40,35%	23	1,75%	1	70,17%	4,0	1,1	4	2	3	5	56,14%	32	21,05%	12	5,26%	3	17,54%	10
1.4.3. Can be used in real time	1.4.3. The tool can be used in real time (i.e. real-time data tracking), e.g. if the user is experiencing a health issue, reducing waits and sometimes harmful delays for both those who receive and those who give care	3,51%	2	3,51%	2	15,79%	9	29,82%	17	43,86%	25	3,51%	2	73,68%	4,1	1,0	4	1	4	5	33,33%	19	31,58%	18	24,56%	14	10,53%	6
1.5.1.a. Easy to navigate	1.5.1.a. It is easy to navigate through the tool (e.g. to move from one location to another and to move backwards, and the design is responsive to the screen size used)	0,00%	0	1,75%	1	1,75%	1	24,56%	14	70,18%	40	1,75%	1	94,74%	4,7	0,6	5	1	4	5	77,19%	44	17,54%	10	1,75%	1	3,51%	2
1.5.1.b. Learnability	1.5.1.b. Learning to use the tool is easy and does not require a lot of time, appropriate explanations appear if needed	1,75%	1	3,51%	2	1,75%	1	31,58%	18	59,65%	34	1,75%	1	91,23%	4,5	0,9	5	1	4	5	73,68%	42	21,05%	12	1,75%	1	3,51%	2
1.5.2.a. Visual design is appealing	1.5.2.a. The visual design is appealing and has a harmonious look and feel (including colours, and fonts are appropriately sized for the target audience)	1,75%	1	5,26%	3	15,79%	9	42,11%	24	33,33%	19	1,75%	1	75,44%	4,0	0,9	4	1	4	5	70,18%	40	24,56%	14	1,75%	1	3,51%	2
1.5.2.b. Well structured	1.5.2.b. To tool's appearance is well structured, and important information is clear and stands out	0,00%	0	3,51%	2	5,26%	3	31,58%	18	57,89%	33	1,75%	1	89,47%	4,5	0,8	5	1	4	5	71,93%	41	19,30%	11	1,75%	1	7,02%	4
2.1.1.a. High quality interactive features	2.1.1.a. The tool includes high quality interactive features (enables user input and reaction) and is presented in an engaging way (e.g., contains the right mix of video/audio/text/graphics)	1,75%	1	8,77%	5	15,79%	9	43,86%	25	29,82%	17	0,00%	0	73,68%	3,9	1,0	4	2	3	5	54,39%	31	40,35%	23	3,51%	2	1,75%	1
2.1.1.b. Customisability	2.1.1.b. The tool is customisable and allows the user to control all the necessary settings and features (e.g., notifications, alerts, sounds, colours, and fonts)	5,26%	3	8,77%	5	24,56%	14	31,58%	18	29,82%	17	0,00%	0	61,40%	3,7	1,1	4	2	3	5	63,16%	36	22,81%	13	3,51%	2	10,53%	6
2.1.1.c. Evidence for user engagement	2.1.1.c. There's evidence for collaboration with users in the tool's development (e.g. a strong and balanced advisory board with clinical/patients/technical team members able to lead the product design and development)	1,75%	1	5,26%	3	12,28%	7	19,30%	11	61,40%	35	0,00%	0	80,70%	4,3	1,0	5	1	4	5	64,91%	37	22,81%	13	8,77%	5	3,51%	2
2.1.2.a. Persuasiveness and behavioural change	2.1.2.a. The tool is persuasive and aims at understanding what influences people's behaviour and decision making, and then uses this information to design compelling user interactions by offering relevant and customisable therapeutic activities and encouraging users to complete them (e.g. through incentivization, gamification...)	5,26%	3	3,51%	2	21,05%	12	28,07%	16	42,11%	24	0,00%	0	70,18%	4,0	1,1	4	2	3	5	31,58%	18	43,86%	25	15,79%	9	8,77%	5
2.1.2.b. Ongoing feedback and call to action	2.1.2.b. The tool provides appropriate ongoing feedback and appropriate call to action based on the user's state and activities (e.g. provides guidance based on user-entered information)	0,00%	0	0,00%	0	22,81%	13	33,33%	19	43,86%	25	0,00%	0	77,19%	4,2	0,8	4	1	4	5	26,32%	15	52,63%	30	14,04%	8	7,02%	4
2.1.3.a. Design appropriateness and accessiblity	2.1.3.a. The tool's content and design are appropriate for the target audience and accessible to vulnerable populations (e.g. adjust text size, text to voice, colourblind colour scheme adjuster, any specificity for use by minors, offline features). i.e. It takes the user context into account and does not vary in quality because of personal characteristics such as disabilities or vision problems etc.	0,00%	0	0,00%	0	1,75%	1	28,07%	16	70,18%	40	0,00%	0	98,25%	4,7	0,5	5	1	4	5	64,91%	37	24,56%	14	7,02%	4	3,51%	2
2.1.3.b. Culturally relevant factors	2.1.3.b. The tool takes into account culturally relevant factors (e.g. different languages and alphabets, specific religious or cultural requirements or restrictions)	1,75%	1	5,26%	3	15,79%	9	21,05%	12	56,14%	32	0,00%	0	77,19%	4,2	1,0	5	1	4	5	57,89%	33	33,33%	19	0,00%	0	8,77%	5
2.1.4. Fosters HCP-patient interaction	2.1.4. The tool has the ability to foster the interaction between the health care professionals and their patients (e.g. communication features, feedback options)	1,75%	1	1,75%	1	10,53%	6	40,35%	23	45,61%	26	0,00%	0	85,96%	4,3	0,9	4	1	4	5	28,07%	16	43,86%	25	26,32%	15	1,75%	1
2.2.1. User research	2.2.1. The tool has been trialled and tested in a real world setting, and its effectiveness was verified by evidence in published scientific literature (e.g. usability studies and user research)	0,00%	0	0,00%	0	5,26%	3	15,79%	9	78,95%	45	0,00%	0	94,74%	4,7	0,6	5	0	5	5	42,11%	24	31,58%	18	24,56%	14	1,75%	1
2.2.2. Clinical evidence	2.2.2. The tool's clinical effectiveness is supported by strong research (e.g. pre-registered RCTs - Randomised Controlled Trials) with adequate statistical power conducted by credible sources, in which the tool was found to be superior to an appropriate placebo or equivalent to acceptable evidence-based treatment groups	1,75%	1	0,00%	0	12,28%	7	17,54%	10	66,67%	38	1,75%	1	84,21%	4,5	0,9	5	1	4	5	29,82%	17	29,82%	17	38,60%	22	1,75%	1
2.2.3.a. Gone through the proper certification proces	2.2.3.a. The tool clearly identifies the risks that its management may pose for user safety and has gone through the proper certification processes to ensure its safety (e.g. software as a medical device, third-party certification by a medical or governmental organisation)	0,00%	0	0,00%	0	0,00%	0	17,54%	10	82,46%	47	0,00%	0	100,00%	4,8	0,4	5	0	5	5	31,58%	18	33,33%	19	33,33%	19	1,75%	1

2.2.3.b. Disclaimer that the tool does not replace HC	2.2.3.b. The tool contains a disclaimer (or a statement of similar implication) that the information provided/content does not replace a health care professional's judgment	1,75%	1	1,75%	1	15,79%	9	22,81%	13	57,89%	33	0,00%	0	80,70%	4,3	0,9	5	1	4	5	42,11%	24	40,35%	23	17,54%	10	0,00%	0
2.2.3.c. Properly handles potentially dangerous info	2.2.3.c. The tool warns about potential risks when necessary and properly handles potentially "dangerous" information entered by a patient (e.g. when it is necessary to consult a professional), i.e. avoiding injuries to patients from the care that is intended to help them	1,75%	1	0,00%	0	5,26%	3	24,56%	14	66,67%	38	1,75%	1	91,23%	4,6	0,8	5	1	4	5	26,32%	15	43,86%	25	28,07%	16	1,75%	1
2.3.1. Visible users' reviews	2.3.1. The tool's visible users' reviews and ratings are favourable (e.g. a star rating above 4/5 stars). Using users' perceived value through users' reviews and ratings as a proxy for quality, usefulness, or acceptability and popularity.	8,77%	5	17,54%	10	19,30%	11	38,60%	22	14,04%	8	1,75%	1	52,64%	3,3	1,2	4	2	2	4	50,88%	29	21,05%	12	7,02%	4	21,05%	12
2.4.1.a. Verified and endorsed by a health authority	2.4.1.a. The tool has been verified, given a good review, or endorsed by a legitimate/reliable source such as a health organisation or health authority (e.g., APA; FDA in the US; NIH; NHS in the UK; NICE in the UK).	1,75%	1	0,00%	0	22,81%	13	35,09%	20	38,60%	22	1,75%	1	73,69%	4,1	0,9	4	1,25	3,75	5	31,58%	18	29,82%	17	31,58%	18	7,02%	4
2.4.1.b. Possibilities for peer support	2.4.1.b. The tool provides possibilities for peer support and/or social networking	5,26%	3	21,05%	12	29,82%	17	26,32%	15	17,54%	10	0,00%	0	43,86%	3,3	1,1	3	2	2	4	33,33%	19	43,86%	25	8,77%	5	14,04%	8
3.1.1. Cost-benefit analysis	3.1.1. A cost-benefit analysis was performed and led to positive results. I.e. the balance between the costs and benefits arising from the tool's utilisation. This refers to the tool's direct costs (purchase price, subscription, licensing…), but may also include costs associated with the tool's selection, staff training, setting up support mechanisms, and appropriate governance	0,00%	0	5,26%	3	14,04%	8	42,11%	24	35,09%	20	3,51%	2	77,20%	4,1	0,9	4	1	4	5	59,65%	34	12,28%	7	12,28%	7	15,79%	9
3.1.2. Periodic updates and maintenance	3.1.2. The tool gets periodic updates and maintenance both from technical and content perspectives (e.g. last update not older than 12 months)	1,75%	1	0,00%	0	5,26%	3	29,82%	17	63,16%	36	0,00%	0	92,98%	4,5	0,8	5	1	4	5	64,91%	37	21,05%	12	12,28%	7	1,75%	1
3.1.3. Implementation and user base	3.1.3. The tool is implemented and utilised within the target health system under usual care OR a large group of clinicians officially refers patients to utilise it (this can be checked by looking at the unique monthly users and their percentage in relation to the target population in the target health system/market).	7,02%	4	5,26%	3	15,79%	9	36,84%	21	26,32%	15	8,77%	5	63,16%	3,8	1,2	4	2	3	5	38,60%	22	31,58%	18	15,79%	9	14,04%	8
3.1.4. Data accessibility	3.1.4. The tool and its data can be accessed at any time and on different platforms and operating systems (e.g., Android, iOS…)	0,00%	0	12,28%	7	5,26%	3	31,58%	18	50,88%	29	0,00%	0	82,46%	4,2	1,0	5	1	4	5	63,16%	36	22,81%	13	7,02%	4	7,02%	4
3.2.1. Feasibility of implementation planning	3.2.1. Assesses the extent to which the tool can be implemented as intended (i.e., feasibility of implementing the tool at a pre-determined date and time). This can be checked by looking at how long it takes, on average, from the contractual agreement until the tool is fully up and running in a healthcare organisation	3,51%	2	10,53%	6	17,54%	10	24,56%	14	33,33%	19	10,53%	6	57,89%	3,8	1,2	4	2	3	5	57,89%	33	8,77%	5	15,79%	9	17,54%	10
3.2.2. Resources required to scale-up	3.2.2. Assesses the workforce and resources required to scale-up the tool, and the implications for care process and care management (e.g. does the tool fit well into existing workflows, roles and responsibilities, or requires new workflows, new roles etc.)	0,00%	0	5,26%	3	8,77%	5	26,32%	15	52,63%	30	7,02%	4	78,95%	4,4	0,9	5	1	4	5	56,14%	32	10,53%	6	19,30%	11	14,04%	8
3.2.3. Infrastructure readiness	3.2.3. Assesses the readiness of the necessary infrastructure for the tool's implementation. i.e. Whether the tool can fit into existing infrastructure or would require investment in additional infrastructure (if yes, is it low, medium, or high investment)	3,51%	2	0,00%	0	8,77%	5	29,82%	17	52,63%	30	5,26%	3	82,45%	4,4	0,9	5	1	4	5	68,42%	39	10,53%	6	14,04%	8	7,02%	4
3.3.1. Favourable pre-conditions	3.3.1. How favourable are the pre-conditions (strategic, political, and environmental contexts) that influence the scaling up of the eHealth tool. For example, the tool's suitability to the socioeconomic context in question, considerations of foreign languages that the tool needs to support, literacy level, and the local regulatory environment	5,26%	3	7,02%	4	22,81%	13	17,54%	10	43,86%	25	3,51%	2	61,40%	3,9	1,2	4	2	3	5	61,40%	35	12,28%	7	8,77%	5	17,54%	10
3.4.1.a. Provider details availability	3.4.1.a. Contact details of the tool's provider are available, easy to find, and include office address, email, and team members details	0,00%	0	5,26%	3	28,07%	16	31,58%	18	35,09%	20	0,00%	0	66,67%	4,0	0,9	4	2	3	5	70,18%	40	14,04%	8	7,02%	4	8,77%	5
3.4.1.b. Credentials of those involved in developmen	3.4.1.b. Availability of information and credentials of the individuals and organisations involved in the development and funding of the tool (transparency on the involvement of any parties that may lead to conflict of interest, e.g. commercial sponsors and partners, financial disclosure)	3,51%	2	7,02%	4	15,79%	9	36,84%	21	36,84%	21	0,00%	0	73,68%	4,0	1,1	4	2	3	5	66,67%	38	14,04%	8	12,28%	7	7,02%	4
3.4.2. Ethical conduct and regulatory compliance	3.4.2. The provider respects ethical conduct, clinical responsibility, and the rules and regulations protecting patient's rights and societal interests (e.g., the tool was approved or certified by a regulatory body in the case of software as a medical device, GDPR, HIPAA…)	0,00%	0	0,00%	0	7,02%	4	15,79%	9	77,19%	44	0,00%	0	92,98%	4,7	0,6	5	0	5	5	71,93%	41	19,30%	11	8,77%	5	0,00%	0
3.4.3.a. Developer interaction quality	3.4.3.a. Interaction quality between the provider and the users, including responsiveness, after sales services, and customer orientation (e.g. provider responds to direct requests/messages swiftly and professionally)	0,00%	0	10,53%	6	12,28%	7	36,84%	21	38,60%	22	1,75%	1	75,44%	4,1	1,0	4	1	4	5	68,42%	39	22,81%	13	5,26%	3	3,51%	2
3.4.3.b. Proactive approach to user needs	3.4.3.b. Demonstration of excellence in a proactive approach to the assessment of user needs, and continuous learning (e.g. provider continuously takes user feedback into account in the periodic updates and iterations of the tool and communicates this to the users)	0,00%	0	7,02%	4	12,28%	7	38,60%	22	40,35%	23	1,75%	1	78,95%	4,1	0,9	4	1	4	5	71,93%	41	19,30%	11	3,51%	2	5,26%	3
3.4.4. Provider eHealth or healthcare experience	3.4.4. The tool's provider has specific experience in the eHealth field OR academic institution (e.g., university) OR health care system (or large health providers' organisation).	15,79%	9	19,30%	11	26,32%	15	17,54%	10	21,05%	12	0,00%	0	38,59%	3,1	1,4	3	2	2	4	61,40%	35	21,05%	12	8,77%	5	8,77%	5

Supplementary Table 2: Key themes and quotes considering the key directional decisions and challenges as expressed by the expert panel participants in the one-to-one interviews (6 pages)

Frequencies reflect the number of participants who favored a specific direction.

EE = eHealth Expert
HCP = Healthcare professional
IE = Insurance Expert
In = Investor
PA = Patient Advocate
Ph = Pharma
RE = Regulatory Expert
Rs = Researcher
TP = Tech Provider

**Universal versus contextual criteria**		
	Favors contextual (n=55, 100%)	"I think it's very fair and it has to be very clear if this is going to be useful, that there is a specific distinction between assessing the inherent functionality of the specific digital health tool and solution, which I would assume mostly captured through the core criteria versus assessing how the solution engage with its context with the ecosystem that's going to adopt it" P9-EE-Rs<br><br>"So, it makes sense because healthcare is local. I mean, there is a high variability of the practice of healthcare from one side to the other, even in the same city" P11-HCP<br><br>"You always kind of-- you have to be going back and forth between these criteria. But at the same time, you're constrained by some of those contextual criteria. So, it will work maybe well in Germany, but it's not going to work well in France" P38-Ph<br><br>"The contextual factors for me as well really resonated… and the contextual, I absolutely support because … it could make or break successful implementation and eventually sustainability and scale up. So, I absolutely like the idea of having a minimal set of items or criteria that address the contextual characteristics of where and how the tool will be implemented" P54-Rs
**Single score versus scorecard**		
	Favors scorecard (n= 43, 78%)	"it's challenging to come up with one size fits all. So, I think you're giving it an opportunity to tease that out to take those differences into consideration" P2-EE<br><br>"I think just having a single score would be very one dimensional… And with the scorecard, so you're taking different perspective into account, which I think is very important" P5-EE<br><br>"The value of an assessment tool is not just a score… It's understanding the breakdown" P6-EE-In<br><br>"A tool that helps you design for quality and focus on the right quality metrics, then by definition, you want to know where you stand on each individual dimension so that you can pick the right dimensions and assess for quality" P10-EE<br><br>"I like the scorecard idea, and I totally appreciate how it's super contextual, what things assessors will value … and where they're willing to give some leeway" P14-HCP-EE<br><br>"I think the scorecard is giving more bigger image about the evaluation and the assessment… it's nearer to the reality. It can give us better idea" P19-HCP-Rs

		"Because you've got so many moving parts... And one part kind of drags the other one in one direction, but the other one drags it in the other… A single score, may not do justice" P18-HCP-EE<br><br>"I think that this kind of a tool is valuable, especially in that it gives the understanding of the strengths and on the weak points of each solution… I'm strongly in favor of the scorecard view because this gives you the ability to zoom in on those items, then you know that this is something that I really need to develop in order to be more successful in this assessment" P27-In<br><br>"The scorecard is probably a bit more suitable and provides a fairer evaluation because it takes into consideration the various criteria" P28-In-TP<br><br>"I think it's a good idea to have the scorecard… because I think that there's so many different criteria, that the only way to do it is to have some kind of scoring system that you can see them very quickly, where you need to improve on" P36-PA-TP<br><br>"A global number would not mean anything, so that doesn't make sense. And depending who's using the tool, obviously, there are different priorities… the scorecard shows that this assessment is comprehensive and it takes into consideration the voices of different parties, the customer, the doctors, and so on and so forth" P34-PA-TP<br><br>"So single-score doesn't work because there is no single objective for two different products. So … you as a project owner, you will be able to identify what's your priority. So, because it's your priority, you will be able to choose what is important to you" P41-Ph-TP<br><br>"The scorecard just gives you a broader mapping, right, than a single score is going. It's not perfect, but it definitely offers a better understanding" P42-RE<br><br>"To me, the scorecard is the solution. And yes, there are tradeoffs, but you at least have higher chances to really elaborate on a more holistic view than just the single score" P46-TP<br><br>"So, kind of some of these areas might be more relevant depending on a bit on the history and the context, so that's why at least highlighting those in the kind of scorecard format instead of single score might make actually more sense" P52-TP
	Favors a mix of scorecard and composite score<br>(n= 12, 22%)	"I'm a big fan of the scorecard … the risk there is if you have a lot of solutions that are being assessed (comparability becomes challenging)" P49-TP-EE<br><br>"That's like a bit of both. So, I'm used to using scores when they'll have a sub scales, and so it'll probably give me, for example, overall symptom burden, but when I break it down… I might have a score for the physical symptoms and then the more psychological symptoms. So, I think, ideally, there will be a lot of tools work as you get an overall score. But you get sub scales are valid in their own right. And I like a bit of both" P53-HCP-Rs<br><br>"Adding an individual score is probably-- but it might give a quick reference for somebody who's comparing multiple solutions to sort of say: Okay, here's the overall score now. Let me go into depth and see where that score is coming from… if they're comparing several apps at the same time" P54-Rs
**Proactive versus mass appraisal**		
	Favors proactive appraisal<br>(n= 36, 65%)	"I may be biased here with an investor hat on, but it's due diligence in my world. And we would never-- even in the first instance, we're sourcing information from the provider. And it's the only way to do it. You have to kind of lift the stones" P6-EE-In

		"I think you'll never get down to the point of really understanding what the app can offer if you don't talk to the developer. I think we've seen this firsthand in different cases" P7-EE<br><br>"I think the point that the mass appraisal is really dependent on the tool promotion, more than the tool efficacy or the tool usefulness, and I think the approach to choose the proactive appraisal, I think it's the better way to assess or to appraise the tool" P14-HCP-Rs<br><br>"I think also the other pieces when I think about it, because I'm also a clinician, I have to put hands on it to verify" P15-HCP-EE<br><br>"I would agree to the focused appraisal because the more specific the testing is, the more information you get from it and the higher the quality of the information also is" P16-HCP-EE<br><br>"I think the proactive is very important because that enables you to go beyond just the way it's marketed" P17-HCP-EE<br><br>"I don't think you can come up with ratings without actually having tried it because, again, there's bias in how things are going to be presented, and you need to see for yourself" P20-HCP-Rs<br><br>"I think that proactive, more hands-on approach is so important. Because there's also a lot that you can find online, and when you see the reality of the tool can be very different" P47-TP<br><br>"They need to look at this proactively, gather the information, look at the evidence supporting it, underpinning what the app does, the intervention, but also the evidence of the app in terms of the claims that it's making" P53-HCP-Rs<br><br>"I definitely see the value of the proactive. I can't think of any other way to not go the proactive route" P54-Rs
	Disfavors proactive appraisal<br>(n= 4, 7%)	"50 criteria are already a lot to do to an assessment in the real world. If you have an academic company, or if you have a regulatory mean, I think then 50 is easy because the more accurate, but in, as you said, with budget and stuff like that, I'm not sure if 50 or now it looks like 33. And 33 is if you do them in depth, that's a lot" P24-IE<br><br>"I mean, if for example, the solution doesn't give any data and then you have to do this by yourself, I'm not sure that some of the assessors will do that hands-on. That's a bit challenging" P50-TP-Ph
	Favors a mix of proactive and mass appraisal<br>(n= 10, 18%)	"For me, the first suggestion (mass appraisal), it could be a little bit superficial. It sounds to me like a bit desk research, but not more. But then, you would still need do this the other approach (proactive) which, of course, requires more time, but then you have a really matching feedback, which is really about platform or about the tool that was developed. I think the combination of the two would be ideal, I would say" P5-EE<br><br>"Mix does sound good. I mean, it's a nice way to also leverage these new technologies, and it lets you kind of do the foundational, but kind of you can go a step above if you want" P14-HCP-EE<br><br>"So having the screening process mass appraisal in a high-quality way is a good start. But then I think the proactive approach on top of it to validate is very valuable" P23-HCP-TP

		"I think that focused versus mass-- maybe a combination of the two, but then defining that criteria for what is focused and what's mass might take time, right? But I think it'll pay off eventually" P28-In-TP<br><br>"For me the mass appraisal might be the first step if you really have to give me the huge volume. But in any case… then you need the proactive part afterwards" P31-PA-EE<br><br>"This is a very critical part because, three months ago, things are completely different. So, I would be the proactive-- mass appraisal doesn't work because with whatever is screening all the internet and try to get the information is not good… But now, with what's happening with the new AI revolution that is taking place and the ability to start controlling what type of information you are able to collect, it's very debatable that when we are launching these guidelines, things will completely change. So, a mass appraisal could work" P41-Ph-TP<br><br>"The focused trials, while they're necessary for, as you said, ease of use or some interoperability tests maybe, they're not required, so you can very well launch that product in the market without-- or with having done very little focused appraisal. So, I agree that, in an ideal world, it is better, it is necessary, and that in the field, if we're looking at the technologies that are being launched and especially in the B2C context, I think a lot of manufacturers get away with measuring outcomes without human subjects' intervention or with very little human subjects' intervention" P42-RE
**Subjective criteria**		
	Assessor diversity<br>(n= 34, 62%)	"I think that you would need to test it through diverse populations… Even if it's just a small group, I think that will be okay, but just to be aware of this diversity" P5-EE<br><br>"I think definitely diversity in the team assessing the tool… particularly for a patient-facing tool, a good demographic mix of patients. I think that's probably the most important" P12-HCP<br><br>"Maybe depending on who the tools might be designed for it might be interesting to have different user groups or user personas" P14-HCP-EE<br><br>"And also, thinking about diversity and inclusion. As you say, making sure that you think about this from a socioeconomic perspective, from an age perspective, from an education perspective, often from an ethnicity perspective, all of these. And you have to understand or have a reasonable level of understanding what level of variability you're going to get" P38-Ph<br><br>"So, I do think having multiple people kind of from a diversity of perspectives rate that criteria would be helpful" P45-Rs<br><br>"For instance, just taking a sample of end user and basically, they test the solution and provide the feedback" P55-Ph
	Research evidence and validation<br>(n= 20, 36%)	"All sort of user experience-related, it should be the end user has evaluated that or scored it through any number of mechanisms, through a survey through, through a usability test, through a user research study" P10-EE<br><br>"I guess, most of the vendors, they just published data about usability, for example. If they use the usability score, which is the universal measurement-- and you cannot use the usability score if you don't<br>follow the steps, and that should be a public information. And then you can say, yeah, this solution, they measure the usability, they publish this, and that's the score. For me, that's reliable data" P50-TP-Ph
	Specify and explain to reach a common understanding<br>(n= 16, 29%)	"I think making it clear is key: This is what you're supposed to be doing. This is what we're measuring, and these are your tasks" P23-HCP-TP

		“There are some broad, I guess, design considerations about software which you can't ignore. If you're going to be required to navigate up and down for menus or enter data repeatedly to get to the next level, these are basic software design considerations where you can have rules-- sort of you have a set of rules which are well-established rules which you follow” P38-Ph<br><br>“I think you can actually create some parameters… you can actually sort of break out into sort of: (Here's three boxes), so you could sort of narrow that down” P48-TP<br><br>“If you assess, for example, usability, and you don't follow certain procedure, you cannot say usability is low or high because you didn't follow the needed steps to assess this…. when you are an assessor of usability, you should follow certain steps” P50-TP-Ph<br><br>“I would say that, I guess in general, if there are clear examples, like what is meant with some of those… that might help to steer it towards same kind of understanding and how different people approach that kind of question” P52-TP
	Tool’s ratings as proxy criteria (if critical mass is achieved) (n= 9, 16%)	“Customer reviews can be so important because that's just the real-world utilizers as opposed to these very motivated people who agreed to participate in a study and even know how to participate in a research study and have access to advertisements for things like that” P20-HCP-Rs<br><br>“From my experience, unless you've got something with thousands of reviews, it's pretty easy for these star ratings to be skewed, especially if somebody's annoyed with the product or the provider” P30-PA<br><br>“If there are overall ratings of usability by a critical mass and it's high, I would say that's a good indicator that it likely has good usability” P54-Rs
	Tool’s use metrics as proxy (n= 4, 7%)	“We had a framework on verification validation, and very recently, we were looking into-- there could be a great tech tool technical components of it that they can also deliver good health outcomes, but if people don't use it, you're not able to collect the data... So, for us, when we were discussing about usability and utility criteria—we were thinking about: Do they enjoy using it based on the score? Will they use it again if they had to? Did they finish the complete sessions?... Or how many times they were hitting that button on tech support? I think those were objective things that we were analyzing” P8-EE<br><br>“If you have a solution prescribed, for example, what is the rate of declining to take that into use, or what is the rate of non-registering? What is the rate of going from registration to actually use? What is the range from going to taking that actually into use and being engaged within a relevant time frame? Let's say in a 12week engagement or something like that” P27-In
**Optionality of some criteria**		
	Favors optionality (n= 39, 71%)	“I think we can't get away from having the optionality. I feel, however, there needs to be some rationale if something is selected as not relevant or not applicable. That's, I think, the risk mitigation around having optionality” P6-EE-In<br><br>“I think you can give the option to exclude criteria and say it's not applicable but with a mandatory request for justification” P9-EE-Rs<br><br>“My impression is that we will not escape the not applicable. Because eHealth tools are so various and the use case are limitless. So, we will not escape it” P11-HCP<br><br>“I think it's good to have flexibility… because not everything is going to be black and white. There's always going to be gray, and you don't want to exclude something because it's not black or white” P12-HCP

		"I would argue there is no way around actually being able to exclude stuff. And I know this makes the scientific evaluation very hard. But yeah, I have very clear opinion there" P22-HCP-TP<br><br>"I think there's so much variety in these devices and this medical solution. So yeah, I think it would also make this a little bit of optional and this is not that it makes it weaker or something like this" P34-PA-Rs<br><br>"I completely agree on the optionality thing. I would say in those where criteria might be because we talked a little bit about the different use cases where it's applicable or not. So, you cannot just throw one framework at every use case, as we discussed. But I like the idea of the optionality because, as you said, it gives the point for reflection. That means basically if you skip it, you skip it why" P37-Ph<br><br>"I'm supporting optionality for the fact that the context or let's say the niche in a different disease area are fulling different" P52-TP
	Disfavors optionality<br>(n= 4, 7%)	"You don't do this, you don't give it, because if you do optionality, you say that, yeah, you can misunderstand it. So, you're allowed to not understand it… So, you give way, you give leeway to the assessor" P24-IE<br><br>"I would put everything mandatory, but then I would leave a kind of open space in case the one who are doing the assessment might have some observations to include" P32-PA-EE<br><br>"This can bring a lot of variability, which I'm not sure it's good for the framework" P50-TP-Ph
**Current versus progressive criteria**		
	Favors current criteria<br>(n= 50, 91%)	"I think that the criteria should reflect the reality of today" P11-HCP<br><br>"For the clinical decision making, this is a legal challenge that can't be met by any digital applications" P16-HCP-EE<br><br>"… in terms of where we are today and in terms of making this framework as useful and relevant today. I think it makes sense... that's the reality of today. Maybe that will be different in the next 5 to 10 years. But probably this is an evolving tool. So maybe that's something worth revisiting then" P27-In<br><br>"I think it is a living list. I think it's going to have to be evaluated on a regular basis. Something that is patient safety today might not be patient safety tomorrow. Things change, technological advances, medical advances, especially with the world we're going into genomics and AI and precision medicine and all of these domains" P28-In-TP<br><br>"I think we don't want to be dehumanizing the way that healthcare is delivered. I mean, I think that technology should always anyway augment, complement the interaction between-- broadly, the interaction between humans. But obviously, in this case, between healthcare professionals and patients" P38-Ph
	Favors progressive criteria<br>(n=3, 5%)	"It's very shortsighted… I think we are already at a point where AI is more precise. People make mistakes all the time. And the more under pressure they are, more mistakes they make… so, when you take that into consideration, this notion that somehow human knows better seems outdated" P2-EE<br><br>"I side with the visionary who's looking down the trail" P30-PA

Supplementary Table 3: Round 2 survey results (2 pages)

Assessment Criteria - short version	Assessment Criteria - complete version	1 I suggest this criterion is excluded		2		3		4		5 This criterion is extremely relevant		I can't assess		Consensus	MEAN	STDEV	MEDIAN	IQR	Q1	Q3	all risk tiers (A, B, and C)		only B and C		only C		I am not sure	
**Re-rated criteria**																												
1.1.4. Possibility to give instant feedback	1.1.4. The tool allows the possibility to give instant feedback to the developers (e.g., provider messaging to report technical issues or errors, inaccuracies or inconsistent workflows)	3,64%	2	5,45%	3	21,82%	12	41,82%	23	27,27%	15	0,00%	0	69,09%	3,8	1,0	4	2,0	3,0	5								
1.2.2.b. Considers related health issues	1.2.2.b. The tool considers multiple health issues and related ones, and sufficiently addresses them to help meet the intended purpose without overwhelming the user (i.e. consider comorbidities, and features that may improve overall quality of life, e.g. adding breathing exercises in a remote patient monitoring tool for lung cancer patients)	3,64%	2	3,64%	2	21,82%	12	30,91%	17	38,18%	21	1,82%	1	69,09%	4,0	1,1	4	2,0	3,0	5								
1.3.1.c. Sufficient information	1.3.1.c. There is sufficient information throughout the tool without any omissions, over-explanations, or irrelevant data	1,82%	1	5,45%	3	10,91%	6	40,00%	22	38,18%	21	3,64%	2	78,18%	4,1	1,0	4	1,0	4,0	5								
1.4.2.b. Allows different platforms	1.4.2.b. The tool allows the users to move across different platforms to allow portability of the tool while retaining their data (e.g. mobile app vs web app, iOS vs Android, older smartphones)	0,00%	0	7,27%	4	20,00%	11	20,00%	11	50,91%	28	1,82%	1	70,91%	4,2	1,0	5	2,0	3,0	5								
1.4.3. Can be used in real time	1.4.3. The tool can be used in real time (i.e. real-time data tracking), e.g. if the user is experiencing a health issue, reducing waits and sometimes harmful delays for both those who receive and those who give care	7,27%	4	3,64%	2	21,82%	12	27,27%	15	36,36%	20	3,64%	2	63,63%	3,8	1,2	4	2,0	3,0	5								
2.1.1.a. High quality interactive features	2.1.1.a. The tool includes high quality interactive features (enables user input and reaction) and is presented in an engaging way (e.g., contains the right mix of video/audio/text/graphics)	1,82%	1	1,82%	1	21,82%	12	41,82%	23	32,73%	18	0,00%	0	74,55%	4,0	0,9	4	1,5	3,5	5								
2.1.1.b. Customisability	2.1.1.b. The tool is customisable and allows the user to control all the necessary settings and features (e.g., notifications, alerts, sounds, colours, and fonts) except for the features that form an essential part of an intervention (e.g. the tool allows the patients to customise the time of a certain reminder according to their daily routine but doesn't allow them to remove it)	1,82%	1	5,45%	3	21,82%	12	38,18%	21	30,91%	17	1,82%	1	69,09%	3,9	1,0	4	2,0	3,0	5								
2.1.2.a. Persuasiveness and behavioural change	2.1.2.a. The tool is persuasive and aims at understanding what influences people's behaviour and decision making, and then uses this information to design compelling user interactions by offering relevant and customisable therapeutic activities and encouraging users to complete them (e.g. through incentivisation, gamification etc.) in a way that balances engagement vs tool addiction	0,00%	0	7,27%	4	34,55%	19	30,91%	17	25,45%	14	1,82%	1	56,36%	3,8	0,9	4	1,8	3,0	4,75								
2.3.1. Visible users' reviews	2.3.1. The tool's visible and verified users' reviews and ratings are favourable (e.g. a star rating above 4/5 stars in the app store, or the Net Promoter Score - NPS). Using users' perceived value through users' reviews and ratings as a proxy for quality, usefulness, or acceptability and popularity	5,45%	3	16,36%	9	34,55%	19	34,55%	19	9,09%	5	0,00%	0	43,64%	3,3	1,0	3	1,0	3,0	4								
2.4.1.a. Verified and endorsed by a health authority	2.4.1.a. The tool has been verified, given a good review, or endorsed by a legitimate/reliable source such as a health organisation or health authority (e.g. APA; FDA in the US; NIH; NHS in the UK; NICE in the UK) or recommended by trusted Healthcare Professionals	0,00%	0	3,64%	2	10,91%	6	27,27%	15	56,36%	31	1,82%	1	83,63%	4,4	0,8	5	1,0	4,0	5								
2.4.1.b. Possibilities for peer support	2.4.1.b. The tool provides possibilities for peer support and/or social networking (e.g. anecdotal evidence - a space to share experiences like patient forums, groups etc.) and/or supported by patient organisations or advisory groups	3,64%	2	16,36%	9	27,27%	15	29,09%	16	23,64%	13	0,00%	0	52,73%	3,5	1,1	4	1,0	3,0	4								
3.1.3. Implementation and user base	3.1.3. The tool is implemented and utilised within the target health system under usual care OR a large group of clinicians officially refers patients to utilise it (this can be checked by looking at the unique monthly users and their percentage in relation to the target population in the target health system/market). The size of the target population needs to be evidence based	1,82%	1	10,91%	6	21,82%	12	34,55%	19	23,64%	13	7,27%	4	58,19%	3,7	1,0	4	1,5	3,0	4,5								
3.2.1. Feasibility of implementation planning	3.2.1. Assesses the extent to which the tool can be implemented as intended (i.e., feasibility of implementing the tool at a pre-determined date and time). This can be checked by looking at how long it takes, on average, from the contractual agreement until the tool is fully up and running in a healthcare organisation	5,45%	3	10,91%	6	36,36%	20	30,91%	17	10,91%	6	5,45%	3	41,82%	3,3	1,0	3	1,0	3,0	4								
3.3.1. Favourable pre-conditions	3.3.1. How favourable are the pre-conditions (strategic, political, and environmental contexts) that influence the scaling up of the eHealth tool. For example, the tool's suitability to the socioeconomic context in question, considerations of foreign languages that the tool needs to support, literacy level, and the local regulatory environment such as reimbursement	0,00%	0	10,91%	6	14,55%	8	45,45%	25	27,27%	15	1,82%	1	72,72%	3,9	0,9	4	1,8	3,3	5								
3.4.1.a. Provider details availability	3.4.1.a. Contact details of the tool's provider are available, easy to find, and include office address, email, and team members details	3,64%	2	10,91%	6	25,45%	14	32,73%	18	27,27%	15	0,00%	0	60,00%	3,7	1,1	4	2,0	3,0	5								
3.4.1.b. Credentials of those involved in development and funding	3.4.1.b. Availability of information and credentials of the individuals and organisations involved in the development and funding of the tool (transparency on the involvement of any parties that may lead to conflict of interest, e.g. commercial sponsors and partners, financial disclosure)	0,00%	0	12,73%	7	25,45%	14	32,73%	18	29,09%	16	0,00%	0	61,82%	3,8	1,0	4	2,0	3,0	5								
3.4.4. Provider eHealth or healthcare experience	3.4.4. The tool's provider and/or its core team members have specific experience in the eHealth field OR academic institution (e.g., university) OR health care system (or large health providers' organisation). E.g. an experienced medical director that oversees the algorithms / contents / features of the tool	1,82%	1	7,27%	4	18,18%	10	32,73%	18	40,00%	22	0,00%	0	72,73%	4,0	1,0	4	2,0	3,0	5								
**Newly added criteria**																												
1.3.1.d. Content reviewed by patients	1.3.1.d. The content has been reviewed by patients to ensure readability and acceptability	0,00%	0	1,82%	1	5,45%	3	32,73%	18	60,00%	33	0,00%	0	92,73%	4,5	0,7	5	1,0	4,0	5	58,18%	32	32,73%	18	5,45%	3	3,64%	2
1.4.1.d. Enables easy data deletion	1.4.1.d. The tool explicitly and easily enables users to delete their data	1,82%	1	1,82%	1	14,55%	8	25,45%	14	50,91%	28	5,45%	3	76,36%	4,3	0,9	5	1,0	4,0	5	56,36%	31	30,91%	17	3,64%	2	9,09%	5

1.4.3.b. Reliable and available at all times	1.4.3.b. The tool is reliable and available at all times and can handle high levels of traffic and usage, with backup and recovery measures in case of downtime or system failures (e.g. enabling offline functionality, functioning during energy interruption or under difficult environmental conditions)	1,82%	1	3,64%	2	10,91%	6	29,09%	16	54,55%	30	0,00%	0	83,64%	4,3	0,9	5	1,0	4,0	5	47,27%	26	21,82%	12	29,09%	16	1,82%	1
1.4.4. Compliantly allows data sharing	1.4.4. The tool compliantly allows for data sharing and segregation for research use (including data analytics and reporting, quality improvement initiatives, and clinical trials)	0,00%	0	3,64%	2	16,36%	9	34,55%	19	45,45%	25	0,00%	0	80,00%	4,2	0,9	4	1,0	4,0	5	52,73%	29	32,73%	18	10,91%	6	3,64%	2
1.4.5. Metadata definition, findability, and retrievability	1.4.5. Data findability and retrievability: Metadata definition (including e.g. units used, reference to controlled vocabularies, ontologies) and the ability for users to retrieve data with the same granularity specified by the metadata (up to and including raw data sets)	3,64%	2	9,09%	5	25,45%	14	27,27%	15	23,64%	13	10,91%	6	50,91%	3,7	1,1	4	2,0	3,0	5	52,73%	29	27,27%	15	5,45%	3	14,55%	8
1.5.1.c. Usability in the intended clinical setting	1.5.1.c. The tool is usable and accessible in the intended clinical setting (e.g. if it is used by HCPs in a clinical setting, can they use it while wearing gloves, e.g. are buttons big enough and separated)	0,00%	0	7,27%	4	18,18%	10	29,09%	16	41,82%	23	3,64%	2	70,91%	4,1	1,0	4	2,0	3,0	5	49,09%	27	21,82%	12	18,18%	10	10,91%	6
2.1.3.c. Affordability and business model transparency	2.1.3.c. Affordability of the tool taking into account the local socioeconomic context, and clarity on who pays and how do they pay	3,64%	2	1,82%	1	18,18%	10	27,27%	15	49,09%	27	0,00%	0	76,36%	4,2	1,0	4	1,0	4,0	5	70,91%	39	18,18%	10	5,45%	3	5,45%	3
2.2.3.d. Differentiates between clinical and technical feedback	2.2.3.d. The tool differentiates between clinical and technical feedback, and clearly channels clinical feedback that may pose a health risk through the proper channels (e.g. advising the patient to call their care team, go to the ER....) and reviews them for vigilance and post-market surveillance purposes and, where relevant, notify them to competent authorities	3,64%	2	0,00%	0	9,09%	5	34,55%	19	52,73%	29	0,00%	0	87,28%	4,3	0,9	5	1,0	4,0	5	50,91%	28	34,55%	19	12,73%	7	1,82%	1
3.4.5. Availability of phase-out scenarios	3.4.5. Availability of phase-out scenarios, if the tool's provider stops producing/maintaining the tool	1,82%	1	5,45%	3	23,64%	13	29,09%	16	32,73%	18	7,27%	4	61,82%	3,9	1,0	4	2,0	3,0	5	49,09%	27	27,27%	15	9,09%	5	14,55%	8

Supplementary Reference 1: Final list of criteria and proposed assessment instrument (8 pages)

Tool details and risk tier

(according to the selected risk tier some criteria may not apply)

The interactive assessment instrument will be available for download on the project website https://ehealth-criteria-toolbox.net/

**Tool name**	
**Link to the tool's website**	
**Tool description**	*Objectives, use cases, target users, disease area*
**Developer information**	*Details about the developers and their affiliation (e.g. commercial, NGO, university, unknown...)*
**Assessor profile**	*E.g. self-appraisal done by the tool devloper, the assessor is a hospital administrator, a clinician...etc.*
**Date of the assessment**	*Assessments should be periodically revised as eHealth tools are typically often updated and further developed as new technologies emerge*
**Risk tier**	*Risk tier A, B, or C. Please see guidance below to help define which risk tier the tool you are assessing belongs to (according to the selected risk tier some criteria may not apply)*
**Assessor notes**	*Any additional notes about the tool or the developer that the assesor(s) would like to add*

**Tier A tools are** those which have no direct outcome on the patient, but which are intended to save costs or staff time<br><br>(e.g. electronic prescribing systems that do not provide advice to patients, complex scheduling software)	**Tier B tools are** those that assist the public to manage their own health<br><br>(e.g. instant messaging apps for healthcare, symptom or mood diaries and programmes to aid weight-loss or better sleep)	**Tier C tools** are those used for treating and diagnosing medical conditions, with direct health outcomes, and which are likely to be regulated medical devices<br><br>(e.g. symptom monitors which share data with care teams, triaging systems that use patient health data to assist with care decisions and devices that perform diagnostic image analysis for making treatment decisions)<br><br>Tier C DHTs are further split into four sub-groups, namely those which: inform clinical management, drive clinical management, diagnose a condition and treat a condition

For more info please check the NICE ESF risk classification in this link
https://www.nice.org.uk/corporate/ecd7/chapter/section-b-classification-of-digital-health-technologies

https://ehealth-criteria-toolbox.net/

__Disclaimer:__ This assessment instrument and all its components are intended for educational purposes only and are not intended as legal advice. Payers have differing coverage, and reimbursement policies. Laws, regulations, and health insurance policies concerning coverage, coding, and reimbursement are complex and are evolving rapidly. For legal advice, please consult with legal counsel.

The scorecard will <u>automatically</u> reflect the assessment values entered in the sheets "Core criteria - entry" and "Contextual criteria - entry"

Please scroll to see radar charts

Foundational	sub-criteria	mean score	Contextual	sub-criteria	mean score
1. Technical aspects	1.a. Tool functioning accurately and rapidly,<br>1.b. Reliable and available at all times,<br>1.c. Adequate training resources,<br>1.d. Easy to access help		10. Data-protection compliance	10.a. Compliant with applicable privacy laws,<br>10.b. Complianty allows data sharing	
2. Clinical utility and safety	2.a. Clinical evidence,<br>2.b. Properly handles potentially dangerous information,<br>2.c. Differentiates between clinical and technical		11. Safety regulatory compliance	11.a. Gone through the proper certification processes,<br>11.b. Disclaimer that the tool does not replace HCPs	
3. Usability and human centricity	3.a. User research,<br>3.b. Easy to navigate,<br>3.c. Learnability,<br>3.d. Visual design is appealing,<br>3.e. Well structured ,<br>3.f. Evidence for user engagement,<br>3.g. Ongoing feedback and call to action,<br>3.h. Design appropriateness and accessiblity,<br>3.i. Fosters HCP-patient interaction		12. Interoperability and data integration	12. Allows data exchange	
4. Data management	4.a. Clear privacy policy,<br>4.b. Respects informed consent,<br>4.c. Data accessibility,<br>4.d. Enables easy data deletion		13. Cultural requirements	13. Culturally relevant factors	
5. Functionality	5.a. Clear info about features and use,<br>5.b. Functionality is clearly identifiable,<br>5.c. Specific, measurable and achievable goals,<br>5.d. Interactive features are customisable		14. Affordability	14. Affordability and business model transparency	
6. Content	6.a. Content is accurate, complete, consistent, and timely,<br>6.b. Content is appropriate for target audience,<br>6.c. Sufficient information,<br>6.e. Content reviewed by patients,<br>6.f. Quality information from credible sources,<br>6.g. Content reviewed by HCPs,<br>6.h. Content relevant for its specified purpose		15. Cost-benefit	15. Cost-benefit analysis	
7. Endorsement	7. Verified and endorsed by a health authority		16. Implementatability	16.a. Resources required to scale-up,<br>16.b. Infrastructure readiness	
8. Maintenance	8. Periodic updates and maintenance				
9. Developer	9.a. Ethical conduct<br>9.b. Developer interaction quality<br>9.c. Proactive approach to user needs				

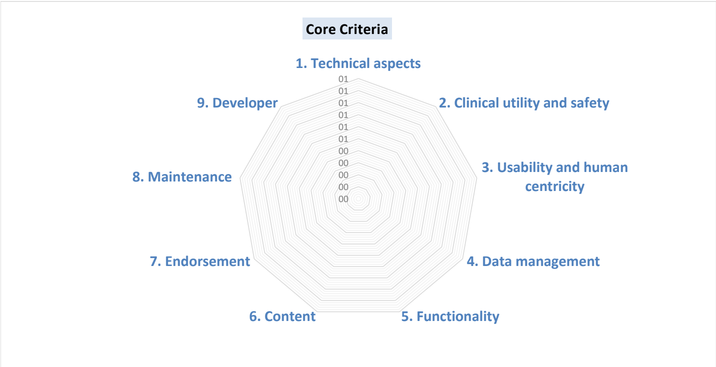

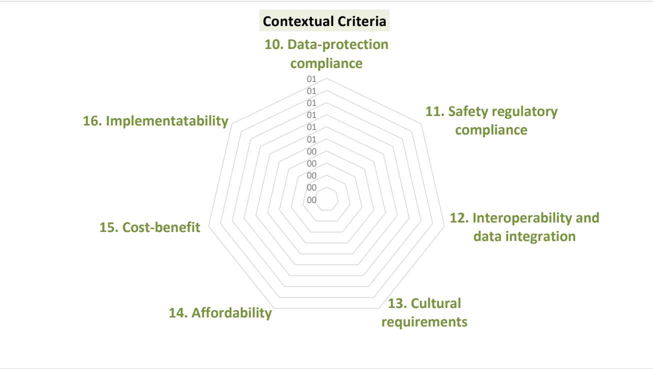

Criteria clusters and sub-criteria	Met the criterion (5/5)	Partially met the criterion (2.5/5)	Did not meet the criterion (0/5)	Score	**Additional guidance for assessors**	Optional	Subjective measure (ensure assessors' diversity)	Requires hands-on trial	May require getting in touch with the developer	Risk tier A	Risk Tier B	Risk Tier C
**1. Technical aspects**	*The assessor may add cluster-specific comments in this area...*											
1.a. Tool functioning accurately and rapidly	The tool is functioning accurately and rapidly, without any error messages, glitches, or crashes (e.g., unexpected stops of running, response time)	The tool is mostly functioning accurately and rapidly, with some error messages, glitches, or crashes (e.g., unexpected stops of running, response time)	The tool is not functioning accurately and rapidly, with many error messages, glitches, or crashes (e.g., unexpected stops of running, response time)		This may sometimes be impacted by local infrastructure like wifi speed - the need here is to assess the tool's technical reliability not that of the infrastructure (which is assessed elsewhere)			x		✓	✓	✓
1.b. Reliable and available at all times	The tool is reliable and available at all times and can handle high levels of traffic and usage, with backup and recovery measures in case of downtime or system failures (e.g. enabling offline functionality, functioning during energy interruption or under difficult environmental conditions)	The tool is reliable and available most of the time (with some exceptions) and can mostly handle high levels of traffic and usage, with backup and recovery measures in case of downtime or system failures (e.g. enabling offline functionality, functioning during energy interruption or under difficult environmental conditions)	The tool is not reliable and available at all times and cannot handle high levels of traffic and usage, with backup and recovery measures in case of downtime or system failures (e.g. enabling offline functionality, functioning during energy interruption or under difficult environmental conditions)					x	x	✓	✓	✓
1.c. Adequate training resources	The tool provides adequate and user friendly training resources for end users (patients and clinicians) to ensure their comfort with basic competencies and skills needed to use the tool effectively (e.g., in the form of training material, tutorials, videos, user guides or documentation)	The tool provides some training resources for end users (patients and clinicians) to ensure their comfort with basic competencies and skills needed to use the tool effectively (e.g., in the form of training material, tutorials, videos, user guides or documentation) but they are not adequate or user friendly	The tool does not provide training resources for end users (patients and clinicians) to ensure their comfort with basic competencies and skills needed to use the tool effectively (e.g., in the form of training material, tutorials, videos, user guides or documentation)							✓	✓	✓
1.d. Easy to access help	It's easy and obvious to access technical help when needed (e.g. hotline, support email, contact form, help section, live chatbot, the technical help needed by a user can be canalized by the HCP to different channels depending on opening hours)	It's not very easy to access technical help when needed (e.g. hotline, support email, contact form, help section, live chatbot, the technical help needed by a user can be canalized by the HCP to different channels depending on opening hours)	It's quite difficult or impossible to access technical help when needed (e.g. hotline, support email, contact form, help section, live chatbot, the technical help needed by a user can be canalized by the HCP to different channels depending on opening hours)		This criterion is about technical support. Clinical support (safety) is addressed elsewhere			x		✓	✓	✓
**2. Clinical utility and safety**	*The assessor may add cluster-specific comments in this area...*											
2.a. Clinical evidence	The tool's clinical effectiveness is supported by strong research (e.g. pre-registered RCTs - Randomised Controlled Trials, observational studies, or large clinical RWE - real world evidence studies) with adequate statistical power conducted by credible sources, in which the tool was found to be superior to an appropriate placebo or equivalent to acceptable evidence-based treatment groups. Interventions should have a positive impact either on how a patient feels, how a patient functions, or how a patient survives.	The tool's clinical effectiveness is supported by some weak research (e.g. pre-registered RCTs - Randomised Controlled Trials, observational studies, or large clinical RWE - real world evidence studies) without adequate statistical power nor conducted by credible sources, in which the tool was found to be superior to an appropriate placebo or equivalent to acceptable evidence-based treatment groups. Interventions should have a positive impact either on how a patient feels, how a patient functions, or how a patient survives.	The tool's clinical effectiveness is not supported by any research (e.g. pre-registered RCTs - Randomised Controlled Trials, observational studies, or large clinical RWE - real world evidence studies), in which the tool was found to be superior to an appropriate placebo or equivalent to acceptable evidence-based treatment groups. Interventions should have a positive impact either on how a patient feels, how a patient functions, or how a patient survives.		The level of evidence will be influenced by how long the tool has been on the market (i.e. tools that have been longer on the market are more capable of showing evidence than newer tools that didn't build the user base yet). In case the tool is still very newly launched, at least a mid-term evidence plan should be presented and re-evaluated after an agreed period of time (e.g. DiGAs in Germany or more recently in France).<br>For further guidance on assessing the quality of clinical evidence and what to look for please consult the Evidence DEFINED framework that provides a standardized approach to assessing evidence for digital health products https://www.nature.com/articles/s41746-023-00836-5				x			✓
2.b. Properly handles potentially dangerous information	The tool warns about potential risks when necessary and properly handles potentially "dangerous" information entered by a patient (e.g. when it is necessary to consult a professional), i.e. avoiding injuries to patients from the care that is intended to help them	The tool warns about some but not all potential risks and does not always properly handle potentially "dangerous" information entered by a patient (e.g. when it is necessary to consult a professional), i.e. avoiding injuries to patients from the care that is intended to help them	The tool does not warn about potential risks and does not properly handle potentially "dangerous" information entered by a patient (e.g. when it is necessary to consult a professional)					x			✓	✓
2.c. Differentiates between clinical and technical feedback	The tool differentiates between clinical and technical feedback, and clearly channels clinical feedback that may pose a health risk through the proper channels (e.g. advising the patient to call their care team, go to the ER....) and reviews them for vigilance and post-market surveillance purposes and, where relevant, notify them to competent authorities		The tool does not differentiate between clinical and technical feedback, and does not clearly channel clinical feedback that may pose a health risk through the proper channels (e.g. advising the patient to call their care team, go to the ER....) and does not review them for vigilance and post-market surveillance purposes					x		✓	✓	✓
**3. Usability and human centricity**	*The assessor may add cluster-specific comments in this area...*											
3.a. User research	The tool's usability and acceptability has been rigorously trialled and tested in a real world setting, and its effectiveness was verified by strong evidence in published scientific literature (e.g. peer reviewed usability studies and user research)	The tool's usability and acceptability has been partially tested in a real world setting, and its effectiveness was verified by weak evidence in published scientific literature (e.g. peer reviewed usability studies and user research)	The tool's usability and acceptability has not been tested in a real world setting, and its effectiveness was not verified by evidence in published scientific literature (e.g. peer reviewed usability studies and user research)		The level of evidence may be influenced by how long the tool has been on the market (i.e. tools that have been longer on the market are more capable of showing evidence than newer tools that didn't build the user base yet). Assessors are advised to have a closer look at such studies to inspect their quality (e.g. sample size, sample diversity, and rigor of the study methodology)				x	✓	✓	✓
3.b. Easy to navigate	It is easy to navigate through the tool (e.g. to move from one location to another and to move backwards, and the design is responsive to the screen size used)	It is not always easy to navigate through the tool (e.g. to move from one location to another and to move backwards, and the design is not always responsive to the screen size used)	It is difficult or confusing to navigate through the tool (e.g. to move from one location to another and to move backwards, and the design is not responsive to the screen size used)		For further details, there are also usability standards that the assessor may refer to. E.g. ISO/TC 210 is a standard for quality management and corresponding general aspects for products with a health purpose including medical devices https://www.iso.org/standard/63179.html		x	x		✓	✓	✓
3.c. Learnability	Learning to use the tool is easy and does not require a lot of time, appropriate explanations appear if needed	Learning to use the tool is not very easy and requires some time, appropriate explanations appear if needed	Learning to use the tool is not easy and requires a lot of time, appropriate explanations does not appear if needed				x	x		✓	✓	✓
3.d. Visual design is appealing	The visual design is appealing and has a harmonious look and feel (including colours, and fonts are appropriately sized for the target audience)	The visual design is somewhat appealing and has a harmonious look and feel (including colours, and fonts are appropriately sized for the target audience)	The visual design is not appealing and does not have a harmonious look and feel (including colours, and fonts are not appropriately sized for the target audience)				x	x		✓	✓	✓
3.e. Well structured	To tool's appearance is well structured, and important information is clear and stands out	To tool's appearance is somewhat well structured, and important information is sometimes clear but doesn't always stand out	To tool's appearance is not well structured, and important information is not clear and does not stand out				x	x		✓	✓	✓
3.f. Evidence for user engagement	There's evidence for co-creation and collaboration with users in the tool's development (e.g. a strong and balanced advisory board with clinical/patients/technical team members able to lead the product design and development)	There's evidence for co-creation and collaboration with some users in the tool's development (e.g. advisory board is not balanced with only some but not all relevant stakeholder groups such as clinical/patients/technical team members able to lead the product design and [illegible]	There's no evidence for co-creation and collaboration with users in the tool's development (e.g. there's no advisory board with clinical/patients/technical team members able to lead the product design and development)							✓	✓	✓

3.g. Ongoing feedback and call to action	The tool provides appropriate ongoing feedback and appropriate call to action based on the user's state and activities (e.g. provides guidance based on user-entered information)	The tool partially provides some feedback and call to action based on the user's state and activities (e.g. provides guidance based on user-entered information)	The tool does not provide any feedback and call to action based on the user's state and activities (e.g. does not provide guidance based on user-entered information)	In some limited cases, depending on the tool's objectives and use cases, this criterion may not be applicable	x		x		✓	✓
3.h. Design appropriateness and accessiblity	The tool's content and design are appropriate for the target audience and accessible to vulnerable populations (e.g. adjust text size, text to voice, colourblind colour scheme adjuster, any specificity for use by minors, offline features, left and right handed options). I.e. it takes the user context into account and does not vary in quality because of personal characteristics such as dexterity, disabilities, movement disorders, or vision problems etc.	The tool's content and design are partially appropriate for some but not all target audiences and not always accessible to vulnerable populations (e.g. adjust text size, text to voice, colourblind colour scheme adjuster, any specificity for use by minors, offline features, left and right handed options). I.e. it somewhat takes the user context into account and may partially vary in quality because of personal characteristics such as dexterity, disabilities, movement disorders, or vision problems etc.	The tool's content and design are not appropriate for the target audience and not accessible to vulnerable populations (e.g. adjust text size, text to voice, colourblind colour scheme adjuster, any specificity for use by minors, offline features, left and right handed options). I.e. it does not take the user context into account and varies in quality because of personal characteristics such as dexterity, disabilities, movement disorders, or vision problems etc.			x	x	✓	✓	✓
3.i. Fosters HCP-patient interaction	The tool has the ability to foster the interaction between the health care professionals and their patients (e.g. communication features, feedback options)		The tool does not have the ability to foster the interaction between the health care professionals and their patients (e.g. there are no communication features, feedback options)	In some very limited cases where the tool being assessed is completely autonomous and is not designed to be integrated in the traditional halthcare setting, this criterion may not be applicable	x				✓	✓
**4. Data management**	*The assessor may add cluster-specific comments in this area...*									
4.a. Clear privacy policy	The tool has a clear privacy policy and informs the users on how their data will be kept confidential and secured and how the data may be used (e.g., for commercial or research purposes)	The tool has a complex privacy policy and the users are not clearly informed on how their data will be kept confidential and secured and how the data may be used (e.g., for commercial or research purposes)	The tool does not have a clear privacy policy and does not inform the users on how their data will be kept confidential and secured and how the data may be used (e.g., for commercial or research purposes)					✓	✓	✓
4.b. Respects informed consent	The tool respects informed consent and allows the user to opt out of data collection (e.g. ability to configure the settings of their data storage, access, and management)	The tool partially respects informed consent and allows the user to opt out of some but not all data collection (e.g. ability to configure some settings of their data storage, access, and management)	The tool does not respect informed consent and does not allow the user to opt out of data collection (e.g. ability to configure the settings of their data storage, access, and management)					✓	✓	✓
4.c. Data accessibility	The tool and its data can be accessed at any time and on different platforms and operating systems (e.g., Android, iOS…)							✓	✓	✓
4.d. Enables easy data deletion	The tool explicitly and easily enables users to delete their data	The tool enables users to delete their data but not in an easy or explicit way	The tool does not enable users to delete their data					✓	✓	✓
**5. Functionality**	*The assessor may add cluster-specific comments in this area...*									
5.a. Clear info about features and use	There is clear information about the tool's features and appropriate ways to utilize it (e.g., adjunct, standalone) and the population it is designed to serve, presented in a concise way not overwhelming to the user	There is some information about the tool's features and appropriate ways to utilize it (e.g., adjunct, standalone) and the population it is designed to serve, but it is presented in an inconcise way and can be overwhelming to the user	There is no information about the tool's features and appropriate ways to utilize it (e.g., adjunct, standalone) and the population it is designed to serve					✓	✓	✓
5.b. Functionality is clearly identifiable	The functionality of each element is clearly identifiable (e.g. if the user must take a specific action, the tool clearly and visually indicates the action to be taken)	The functionality of some, but not all, elements is somewhat identifiable (e.g. if the user must take a specific action, the tool indicates the action to be taken, but not always in a clear way)	The functionality of some elements is not identifiable (e.g. if the user must take a specific action, the tool does not indicate the action to be taken)			x	x	✓	✓	✓
5.c. Specific, measurable and achievable goals	The tool has specific, measurable and achievable goals (desired outcomes) that are specified/obvious within the tool itself	The tool has somewhat specific, measurable and achievable goals (desired outcomes) but they are not specified/obvious within the tool itself	The tool does not have specific, measurable and achievable goals (desired outcomes)					✓	✓	✓
5.d. Interactive features are customisable	Interactive features such as reminders, push notifications, and prompts are customisable and not overwhelming (e.g. users can customise the frequency and timing of reminders to suit their daily routines)	Some, but not all, interactive features such as reminders, push notifications, and prompts are somewhat customisable (e.g. users can customise the frequency or timing of reminders to suit their daily routines)	Interactive features such as reminders, push notifications, and prompts are not customisable					✓	✓	✓
**6. Content**	*The assessor may add cluster-specific comments in this area...*									
6.a. Content is accurate, complete, consistent, and timely	Health-related content is accurate, complete, consistent, and timely (e.g. according to state of the art scientific evidence)	Health-related content is somewhat, but not always, accurate, complete, consistent, or timely (e.g. according to state of the art scientific evidence)	Health-related content is not accurate, complete, consistent, nor timely (e.g. according to state of the art scientific evidence)				x	✓	✓	✓
6.b. Content is appropriate for target audience	The tool's content is provided in a clear and appropriate way for the target audience (using an understandable, plain and simple language, with messages adapted to the user profile in terms of linguistic style and level, facilitating user understanding and avoiding using technicalities)	The tool's content is somewhat, but not always, provided in a clear and appropriate way for the target audience (using a somewhat understandable, plain and simple language, with messages somewhat adapted to the user profile in terms of linguistic style and level, and not always avoiding using technicalities)	The tool's content is not provided in a clear and appropriate way for the target audience (does not use an understandable, plain and simple language, and messages are not adapted to the user profile in terms of linguistic style and level, using technicalities, not facilitating user understanding)			x	x	✓	✓	✓
6.c. Sufficient information	There is sufficient information throughout the tool without any omissions, over-explanations, or irrelevant data	There is somehat sufficient information throughout the tool, but there are some omissions, or over-explanations, or irrelevant data	There is no sufficient information throughout the tool, there are clear omissions, or over-explanations, or irrelevant data			x	x	✓	✓	✓
6.e. Content reviewed by patients	The content has been reviewed by patients to ensure readability and acceptability		The content has not been reviewed by patients to ensure readability and acceptability					✓	✓	✓
6.f. Quality information from credible sources	The tool contains high quality information (e.g. text, feedback, charts, measures, references) from credible and legitimate sources (e.g., WHO)	The tool contains sometimes, but not always, high quality information (e.g. text, feedback, charts, measures, references) and sometimes, but not always, from credible and legitimate sources (e.g., WHO)	The tool does not contain high quality information (e.g. text, feedback, charts, measures, references) nor from credible and legitimate sources (e.g., WHO)						✓	✓
6.g. Content reviewed by HCPs	The content has been reviewed by (or originated from) healthcare professionals with the most updated evidence-based practice of medicine		The content has not been reviewed by (or originated from) healthcare professionals					✓	✓	✓
6.h. Content relevant for its specified purpose	The tool's contents are relevant to the underlying objective and likely to be effective in achieving the specified purpose in the specific intended population	The tool's contents are somehow relevant to some of the underlying objective and may be effective in partially achieving the specified purpose in the specific intended population	The tool's contents are not relevant to the underlying objective and not likely to be effective in achieving the specified purpose in the specific intended population				x	✓	✓	✓
**7. Endorsement**	*The assessor may add cluster-specific comments in this area...*									
7. Verified and endorsed by a health authority	The tool has been verified, given a good review, or endorsed by a legitimate/reliable source such as a health organisation, health authority, scientific/medical society (e.g., APA; FDA in the US; NIH; NHS in the UK; NICE in the UK) or recommended by trusted Healthcare Professionals		The tool has not been verified, nor given a good review, nor endorsed by a legitimate/reliable source such as a health organisation, health authority, scientific/medical society (e.g., APA; FDA in the US; NIH; NHS in the UK; NICE in the UK) nor recommended by trusted Healthcare Professionals	This is an adoption criterion. It is impacted by how long the tool has been on the market (the longer the tool has been on the market, the more likely it will be used and verified or endorsed by health authorities)				✓	✓	✓
**8. Maintenance**	*The assessor may add cluster-specific comments in this area...*									

8. Periodic updates and maintenance	The tool gets periodic updates and maintenance both from technical and content perspectives (e.g. last update not older than xx months depending on the use case, the content is periodically updated with the new findings in the medical field)	The tool partially gets updates and maintenance either from technical or content perspectives (e.g. last update is relatively old, the content is only partially updated with the new findings in the medical field)	The tool does not get periodic updates and maintenance both from technical and content perspectives (e.g. the tools has not been updated in a relatively long time, the content is not updated with the new findings in the medical field)				✓	✓	✓
**9. Developer**	*The assessor may add cluster-specific comments in this area...*								
9.a. Ethical conduct	The tool's provider respects ethical conduct, clinical responsibility, and the rules and regulations protecting patient's rights and societal interests (e.g., the tool was approved or certified by a regulatory body in the case of software as a medical device, GDPR, HIPAA...)	The tool's provider only sometimes, but not always, respects ethical conduct, clinical responsibility, and the rules and regulations protecting patient's rights and societal interests (e.g., the tool was in some cases approved or certified by a regulatory body in the case of software as a medical device, GDPR, HIPAA...)	The tool's provider does not respect ethical conduct, clinical responsibility, and the rules and regulations protecting patient's rights and societal interests (e.g., the tool was not approved or certified by a regulatory body in the case of software as a medical device, GDPR, HIPAA...)				✓	✓	✓
9.b. Developer interaction quality	Interaction quality between the tool's provider and the users, including responsiveness, after sales services, and customer orientation is extremely high (e.g. provider responds to direct requests/messages swiftly and professionally)	Interaction quality between the tool's provider and the users, including responsiveness, after sales services, and customer orientation is acceptable (e.g. provider takes a relatively long time to respond to direct requests/messages, the response is not always very professional)	Interaction quality between the tool's provider and the users, including responsiveness, after sales services, and customer orientation is very low or non-existent (e.g. provider does not responds to direct requests/messages)			x	✓	✓	✓
9.c. Proactive approach to user needs	Demonstration of excellence in a proactive approach to the assessment of user needs, and continuous learning (e.g. provider continuously takes user feedback into account in the periodic updates and iterations of the tool and communicates this to the users)	Demonstration of some engagement in the assessment of user needs, and continuous learning (e.g. provider somewhat takes user feedback into account in the periodic updates and iterations of the tool and sometimes communicates this to the users)	Lack of proactive approach to the assessment of user needs, and continuous learning (e.g. provider does not take user feedback into account in the periodic updates and iterations of the tool)		If this information is not publicly communicated by the tool's provider, the assessor may need to directly ask about how the provider takes user feedback into account and how they communicate the resulting changes to the users	x	✓	✓	✓

Short form	Met the criterion (5/5)	Partially met the criterion (2.5/5)	Did not meet the criterion (0/5)	Score	Additional guidance for assessors	Optional	Subjective measure (ensure assessors' diversity)	Requires hands-on trial	May require getting in touch with the developer	Risk tier A	Risk Tier B	Risk Tier C
**10. Data-protection compliance**	*The assessor may add cluster-specific comments in this area...*											
10.a. Compliant with applicable privacy laws	The tool explicitly reports being compliant with the relevant data privacy and protection laws (e.g. GDPR, HIPAA...), and the treatment of any personal data is compatible with the Patient Data Act, Personal Data Act, and other applicable privacy laws (flexibility to respect multiple data regulations depending on local context)		The tool does not explicitly report being compliant with the relevant data privacy and protection laws (e.g. GDPR, HIPAA...), and it is not clear if the treatment of any personal data is compatible with the Patient Data Act, Personal Data Act, and other applicable privacy laws (flexibility to respect multiple data regulations depending on local context)							✓	✓	✓
10.b. Compliantly allows data sharing	The tool compliantly allows for data sharing and segregation for research use (including data analytics and reporting, quality improvement initiatives, and clinical trials)	The tool partially allows for some data sharing and segregation for research use (including data analytics and reporting, quality improvement initiatives, and clinical trials)	The tool does not allow for data sharing and segregation for research use (including data analytics and reporting, quality improvement initiatives, and clinical trials)							✓	✓	✓
**11. Safety regulatory compliance**	*The assessor may add cluster-specific comments in this area...*											
11.a. Gone through the proper certification processes	The tool's provider clearly identifies the risks that its management may pose for user safety and has gone through the proper certification processes to ensure its safety (e.g. software as a medical device, third-party certification by a medical or governmental organisation)		The tool's provider does not identify the risks that its management may pose for user safety and has not gone through the proper certification processes to ensure its safety (e.g. software as a medical device, third-party certification by a medical or governmental organisation)								✓	✓
11.b. Disclaimer that the tool does not replace HCPs	The tool contains a disclaimer (or a statement of similar implication) that the information provided/content does not replace a health care professional's judgment		The tool does not contain a disclaimer (or a statement of similar implication) that the information provided/content does not replace a health care professional's judgment		In very limited cases, depending on the local laws and the risk level of the tool being assessed, this criterion may not be applicable	x				✓	✓	✓
**12. Interoperability and data integration**	*The assessor may add cluster-specific comments in this area...*											
12. Allows data exchange	The tool allows for interoperability, data integration and exchange of data with other apps, e-tools, wearable devices, electronic health records (ability to exchange data with other systems on a technical and policy level, and with other users such as clinicians or care givers)	The tool only partially allows for interoperability, data integration and exchange of data with some but not all other apps, e-tools, wearable devices, electronic health records (ability to exchange data with other systems on a technical and policy level, and with other users such as clinicians or care givers)	The tool does not allow for interoperability, data integration and exchange of data with other apps, e-tools, wearable devices, electronic health records (ability to exchange data with other systems on a technical and policy level, and with other users such as clinicians or care givers)							✓	✓	✓
**13. Cultural requirements**	*The assessor may add cluster-specific comments in this area...*											
13. Culturally relevant factors	The tool takes into account culturally relevant factors (e.g. different languages and alphabets, specific religious or cultural requirements or restrictions, gender considerations)	The tool takes into account some but not all culturally relevant factors (e.g. different languages and alphabets, specific religious or cultural requirements or restrictions, gender considerations)	The tool does not take into account culturally relevant factors (e.g. different languages and alphabets, specific religious or cultural requirements or restrictions, gender considerations)							✓	✓	✓
**14. Affordability**												
14. Affordability and business model transparency	The tool is affordable taking into account the local socioeconomic context, and it is clear who pays for it and how they pay	The tool is only affordable for some potential users taking into account the local socioeconomic context (e.g. depending on their insurance model), and it is clear who pays for it and how they pay	The tool is not affordable taking into account the local socioeconomic context, and it is not clear who pays for it and how they pay							✓	✓	✓
**15. Cost-benefit**	*The assessor may add cluster-specific comments in this area...*											
15. Cost-benefit analysis	A cost-benefit analysis was performed and led to positive results. I.e. the balance between the costs and benefits arising from the tool's utilisation. This refers to the tool's direct costs (purchase price, subscription, licensing…), but may also include costs associated with the tool's selection, staff training, setting up support mechanisms, and appropriate governance		A cost-benefit analysis was not performed and or has led to negative results. I.e. the balance between the costs and benefits arising from the tool's utilisation. This refers to the tool's direct costs (purchase price, subscription, licensing…), but may also include costs associated with the tool's selection, staff training, setting up support mechanisms, and appropriate governance		This will differ depending on the implementation context and the respective payment model in that specific context (e.g. fee for service with focus on efficiency, vs value-based which would consider the outcomes/incremental health benefit)				x	✓	✓	✓
**16. Implementatability**	*The assessor may add cluster-specific comments in this area...*											
16.a. Resources required to scale-up	The tool fits well into existing workflows and does not require additional resouces (workforce, hardware, software) to scale-up and to enable it to function properly	The tool does not completely fit into existing workflows but only requires low to medium additional resouces (workforce, hardware, software) to scale-up and to enable it to function properly	The tool does not fit well into existing workflows and requires considerable additional resouces (workforce, hardware, software) to scale-up and to enable it to function properly						x	✓	✓	✓
16.b. Infrastructure readiness	The tool fits well into the existing infrastructure and does not require investment in additional infrastructure to enable it to function properly (This refers to physical infrastructure such as electricity, access to power, connectivity etc. in the local context)	The tool partially fits well into the existing infrastructure (hardware, software, and network capabilities) and requires low to medium investment in additional infrastructure to enable it to function properly (This refers to physical infrastructure such as electricity, access to power, connectivity etc. in the local context)	The tool does not fit into the existing infrastructure and requires considerable investment in additional infrastructure to enable it to function properly (This refers to physical infrastructure such as electricity, access to power, connectivity etc. in the local context)							✓	✓	✓

Short form	Full description	Additional guidance for assessors	Risk tier A	Risk Tier B	Risk Tier C
**17. Design**	*The assessor may add cluster-specific comments in this area...*				
17.a. Allows different platforms	The tool allows the users to move across different platforms to allow portability of the tool while retaining their data (e.g. mobile app vs web app, iOS vs Android, older smartphones)		✓	✓	✓
17.b. Can be used in real time	The tool can be used in real time (i.e. real-time data tracking), e.g. if the user is experiencing a health issue, reducing waits and sometimes harmful delays for both those who receive and those who give care	This criterion is optional and may not be applicable depending on the specific use case of the tool being evaluated (e.g. asynchronous eHealth tools are not necessarily designed for real-time data tracking)	✓	✓	✓
17.c. Possibility to give instant feedback	The tool allows the possibility to give instant feedback to the developers (e.g., provider messaging to report technical issues or errors, inaccuracies or inconsistent workflows)		✓	✓	✓
17.d. Usability in the intended clinical setting	The tool is usable and accessible in the intended clinical setting (e.g. if it is used by HCPs in a clinical setting, can they use it while wearing gloves, e.g. are buttons big enough and separated)		✓	✓	✓
**18. Comorbidities**	*The assessor may add cluster-specific comments in this area...*				
18. Considers related health issues	The tool considers multiple health issues and related ones, and sufficiently addresses them to help meet the intended purpose without overwhelming the user (i.e. consider evidence-based comorbidities, and features that may improve overall quality of life, e.g. adding breathing exercises in a remote patient monitoring tool for lung cancer patients)			✓	✓
**19. Data definition**	*The assessor may add cluster-specific comments in this area...*				
19. Metadata definition, findability, and retrievability	Data findability and retrievability: Metadata definition (including e.g. units used, reference to controlled vocabularies, ontologies) and the ability for users to retrieve data with the same granularity specified by the metadata (up to and including raw data sets)		✓	✓	✓
**20. Behavioral and social**	*The assessor may add cluster-specific comments in this area...*				
20.a. High quality interactive features	The tool includes high quality interactive features (enables user input and reaction) and is presented in an engaging way (e.g., contains the right mix of video/audio/text/graphics)		✓	✓	✓
20.b. Customisability	The tool is customisable and allows the user to control all the necessary settings and features (e.g., notifications, alerts, sounds, colours, and fonts) except for the features that form an essential part of an intervention (e.g. the tool allows the patients to customise the time of a certain reminder according to their daily routine but doesn't allow them to remove it)		✓	✓	✓
20.c. Persuasiveness and behavioural change	The tool is persuasive and aims at understanding what influences people’s behaviour and decision making, and then uses this information to design compelling user interactions by offering relevant and customisable therapeutic activities and encouraging users to complete them (e.g. through incentivization, gamification...) in a way that balances engagement vs tool additction	This criterion is optional and may not be applicable depending on the specific use case of the tool being evaluated		✓	✓
20.d. Possibilities for peer support	The tool provides possibilities for peer support and/or social networking (e.g. anecdotal evidence - a space to share experiences like patient forums, groups etc) and/or supported by patient organisations or advisory groups	This criterion is optional and may not be applicable depending on the specific use case of the tool being evaluated		✓	✓
**21. Adoption and implementation**	*The assessor may add cluster-specific comments in this area...*				

21.a. Implementation and user base	The tool is implemented and utilised within the target health system under usual care OR a large group of clinicians officially refers patients to utilise it (this can be checked by looking at the unique monthly users and their percentage in relation to the target population in the target health system/market). The size of the target population needs to be evidence based (e.g. if it's pilot or beta how big is a therapeutic area? size of the technology provider? when did they start selling the tool?)	This is an adoption criterion. Meaning that it is impacted by how long the tool has been on the market (the longer the tool has been on the market, the more likely it will rank higher for this criterion)	✓	✓	✓
21.b. Feasibility of implementation planning	Assesses the extent to which the tool can be implemented as intended (i.e., feasibility of implementing the tool at a pre-determined date and time). This can be checked by looking at how long it takes, on average, from the contractual agreement until the tool is fully up and running in a healthcare organisation	This is an adoption criterion and depends on whether the tool requires a high degree of integration (i.e. may be "not applicable" for some tools). It also depends on the business model of the tool being evaluated (e.g. B2B2C can be quite complex)	✓	✓	✓
21.c. Favourable pre-conditions	How favourable are the pre-conditions (strategic, political, and environmental contexts) that influence the scaling up of the eHealth tool. For example, the tool’s suitability to the socioeconomic context in question, considerations of foreign languages that the tool needs to support, literacy level, and the local regulatory environment such as standard reimbursement processes for eHealth tools	This is a contextual criterion. Its assessment will be different depending on the context that the tool is being considered for	✓	✓	✓
21.d. Visible users’ reviews	The tool's visible and verified users’ reviews and ratings are favourable (e.g. a star rating above 4/5 stars in the app store, or the Net Promoter Score - NPS). Using users’ perceived value through users’ reviews and ratings as a proxy for quality, usefulness, or acceptability and popularity	This is an adoption criterion. Meaning that it is impacted by how long the tool has been on the market (the longer the tool has been on the market, the more likely it will rank higher for this criterion). This criterion is meaningful only when the tool reaches a critical mass (i.e. a large enough number of user reviews)	✓	✓	✓
21.e. Availability of phase-out scenarios	Availability of phase-out scenarios, if the tool's provider stops producing/maintaining the tool		✓	✓	✓
**22. Provider details and experience**	*The assessor may add cluster-specific comments in this area...*				
22.a. Provider details availability	Contact details of the tool's provider are available, easy to find, and include office address, email, and team members details		✓	✓	✓
22.b. Credentials of those involved in development and funding	Availability of information and credentials of the individuals and organisations involved in the development and funding of the tool (transparency on the involvement of any parties that may lead to conflict of interest, e.g. commercial sponsors and partners, financial disclosure)		✓	✓	✓
22.c. Provider eHealth or healthcare experience	The tool's developer and / or its core team members have specific experience in the eHealth field OR academic institution (e.g., university) OR health care system (or large health providers’ organisation). E.g. an experienced medical director that oversees the algorithms / contents / features of the tool		✓	✓	✓

Innovation project supported by Schweizerische Eidgenossenschaft Confédération suisse Confederazione Svizzera Confederaziun svizra Swiss Confederation Innosuisse – Swiss Innovation Agency

Roche kpt: n|w Fachhochschule Nordwestschweiz

eHealth assessment criteria - Delphi process, Round 1

Thank you for participating in this research

You have been invited to contribute because you have been identified as a key opinion leader and a leading eHealth expert with in-depth knowledge and understanding of what it takes to achieve successful adoption and implementation of novel technologies in healthcare. Your input will help shape the discussion around assessing the quality and impact of eHealth technologies, an area that is still being shaped and evolving every day. The survey will take about 25-30 minutes to complete.

Where does the initial list of assessment criteria come from?

To minimise bias and to avoid an initial set of criteria that is impacted by the subjective opinions of the research team, we conducted a systematic literature review to objectively identify the relevant criteria used to assess the quality and impact of eHealth tools.

We searched 5 databases for studies published between 2012 and 2022, yielding 675 results, of which 40 studies met the inclusion criteria. Similar assessment criteria from the different papers, frameworks, and initiatives were aggregated in 36 unique criteria grouped in 13 clusters. The resulting criteria were classified into technical, social, and organisational criteria. The technical assessment criteria were grouped in 5 clusters: technical aspects, functionality, content, data management, and design. The social assessment criteria were grouped in 4 clusters: human centricity, health outcomes, visible popularity metrics, and social aspects. And the organisational assessment criteria were grouped in 4 clusters: sustainability and scalability, health care organisation, health care context, and developer as shown in **Figure 1 below**. https://pubmed.ncbi.nlm.nih.gov/36843321/

We ask you to help us pressure-test and validate this list of criteria by giving us your expert opinion on:

1. Which criteria are relevant and important to be **included** in the validated assessment framework (only criteria rated as 4 or 5 by 75% of the expert panel will be kept) - *an overview of all criteria that you will be rating is shown in Figure 1 below*
2. Which criteria should be **removed**
3. Which criteria should be **added** if it was missed in the initial list of criteria (you will always have the option to add new criteria in every sub-category)
4. The **risk categories** that are valid for each criterion (e.g. the assessment criterion "behavioural change and persuasiveness" for tier C (digital interventions), but may not be relevant for tier A (system technologies) such as e-prescriptions and e-appointment systems) - *an overview of eHealth tools classified by intended purpose and stratified into risk tiers according to NICE ESF is shown in Figure 2 below*

Scope: This project focuses solely on assessing **patient-facing eHealth tools**, including self-management tools and remote eHealth solutions, rather than tools used within and between care providers (e.g., Electronic Health Records), digital biomarkers, or health data analytics systems used at population level.

Figure 1: The list of assessment criteria that was aggregated from the literature (published here: https://pubmed.ncbi.nlm.nih.gov/36843321/)

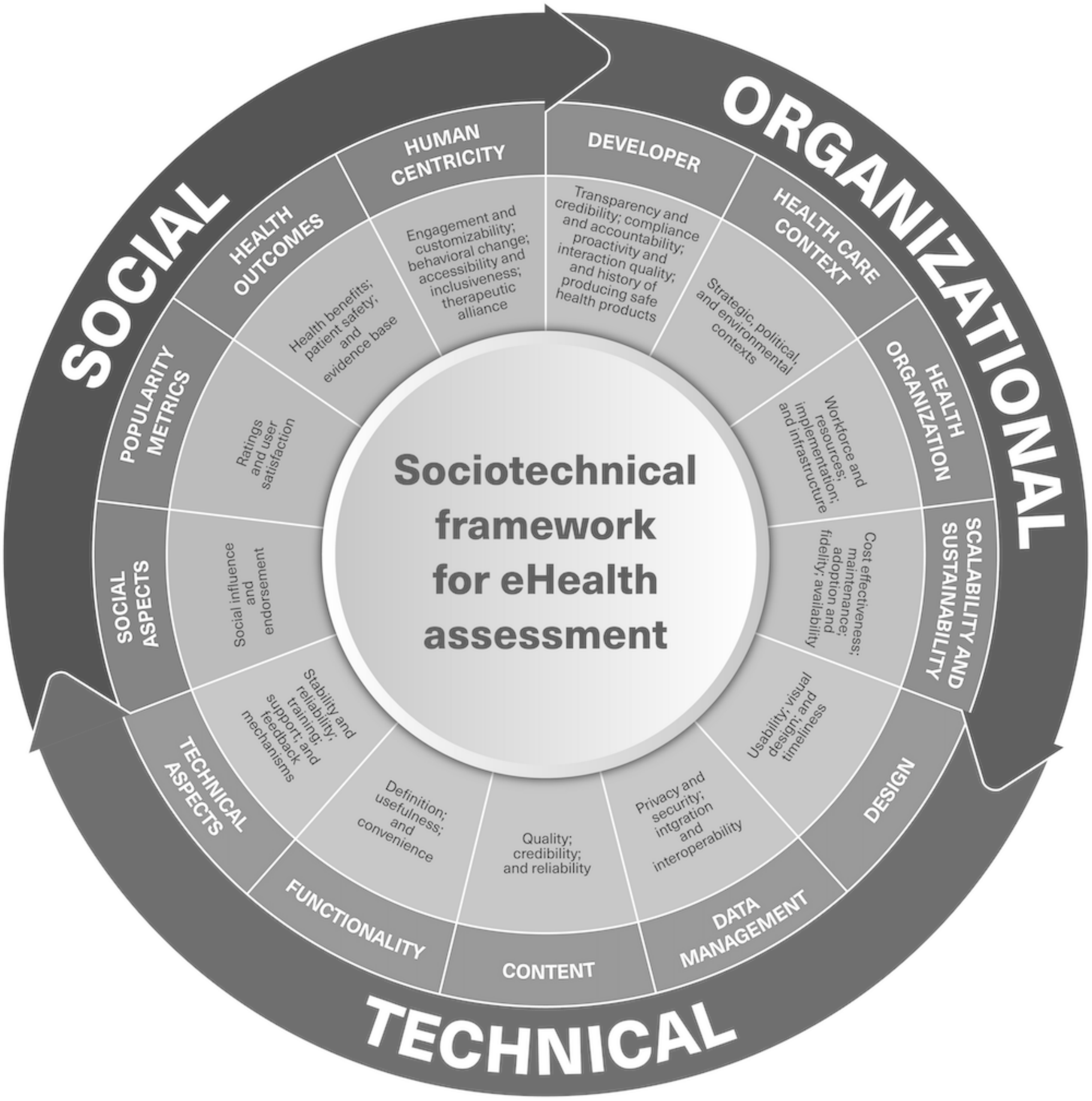

Figure 2: eHealth tools classified by intended purpose and stratified into risk tiers according to NICE ESF (DHTs = Digital Health tools)

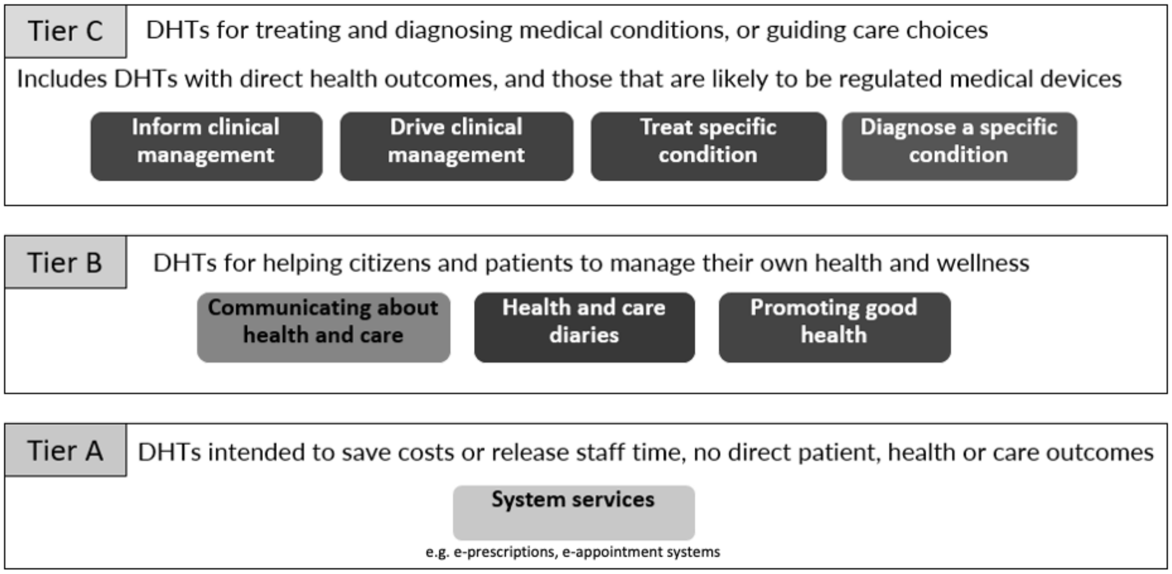

Disclaimer: This research project is intended for educational purposes only and is not intended as legal advice. Payers have differing coverage and reimbursement policies. Laws, regulations, and health insurance policies concerning coverage, coding, and reimbursement are complex and are evolving rapidly. For legal advice, please consult with legal counsel.

This study is part of an overarching research project that is jointly sponsored by F Hoffmann-La Roche Ltd, KPT insurance, and Innosuisse (the Swiss Innovation Agency, grant 104.445 IP-ICT).

eHealth assessment criteria - Delphi process, Round 1

1. Technical Criteria

1.1. Technical aspects

1.1.1. Technical reliability and stability

* 1.1.1. The tool is functioning accurately and rapidly, without any error messages, glitches, or crashes (e.g., unexpected stops of running, response time)

1 *I suggest this criterion is excluded*	2	3	4	5 *This criterion is extremely relevant*	I can't assess
★	★	★	★	★	○

* 1.1.1. The risk categories that are valid for this criterion (select from the dropdown menu)

1.1.2. Training and documentation

* 1.1.2. The tool provides adequate training resources for end users to ensure their comfort with basic competencies and skills needed to use the tool effectively (e.g., in the form of training material, tutorials, videos, user guides or documentation)

1 *I suggest this criterion is excluded*	2	3	4	5 *This criterion is extremely relevant*	I can't assess
★	★	★	★	★	○

* 1.1.2. The risk categories that are valid for this criterion (select from the dropdown menu)

1.1.3. Support and help resources

* 1.1.3. It's easy and obvious to access help when needed (e.g. hotline, support email, contact form, help section)

1 *I suggest this criterion is excluded*	2	3	4	5 *This criterion is extremely relevant*	I can't assess
★	★	★	★	★	○

* 1.1.3. The risk categories that are valid for this criterion (select from the dropdown menu)

1.1.4. Feedback mechanisms

* 1.1.4. The tool allows the possibility to give instant feedback to the developers (e.g., provider messaging to report technical issues or errors)

1 *I suggest this criterion is excluded*	2	3	4	5 *This criterion is extremely relevant*	I can't assess
★	★	★	★	★	○

* 1.1.4. The risk categories that are valid for this criterion (select from the dropdown menu)

1.1.o. Have we missed any important assessment criteria in this sub-category? Or do you have any comments to add to this sub-category?

1.2. Functionality

1.2.1. Feature definition, attributes, functionality, purpose, and user requirements

* 1.2.1.a. There is clear information about the tool's features and appropriate ways to utilize it (e.g., adjunct, standalone)

1 *I suggest this criterion is excluded*	2	3	4	5 *This criterion is extremely relevant*	I can't assess
★	★	★	★	★	○

* 1.2.1.a. The risk categories that are valid for this criterion (select from the dropdown menu)

* 1.2.1.b. The functionality of each element is clearly identifiable (e.g. if the user must take a specific action, the tool clearly and visually indicates the action to be taken)

1 *I suggest this criterion is excluded*	2	3	4	5 *This criterion is extremely relevant*	I can't assess
★	★	★	★	★	○

* 1.2.1.b. The risk categories that are valid for this criterion (select from the dropdown menu)

1.2.2. Feature usefulness, utility, and relevance

* 1.2.2.a. The tool has specific, measurable and achievable goals (desired outcomes) that are specified/obvious within the tool itself

1 *I suggest this criterion is excluded*	2	3	4	5 *This criterion is extremely relevant*	I can't assess
★	★	★	★	★	○

* 1.2.2.a. The risk categories that are valid for this criterion (select from the dropdown menu)

* 1.2.2.b. The tool considers multiple health issues and related ones, and sufficiently addresses them to help meet the intended purpose

1 *I suggest this criterion is excluded*	2	3	4	5 *This criterion is extremely relevant*	I can't assess
★	★	★	★	★	○

* 1.2.2.b. The risk categories that are valid for this criterion (select from the dropdown menu)

1.2.3. Feature convenience

* 1.2.3. Interactive features such as reminders, push notifications, and prompts are customisable and not overwhelming (e.g. users can customise the frequency and timing of reminders to suit their daily routines)

1 *I suggest this criterion is excluded*	2	3	4	5 *This criterion is extremely relevant*	I can't assess
★	★	★	★	★	○

* 1.2.3. The risk categories that are valid for this criterion (select from the dropdown menu)

1.2.o. Have we missed any important assessment criteria in this sub-category? Or do you have any comments to add to this sub-category?

1.3. Content

1.3.1 Content quality

* 1.3.1.a. Health-related content is accurate, complete, consistent, and timely (e.g. according to state of the art scientific evidence)

1 *I suggest this criterion is excluded*	2	3	4	5 *This criterion is extremely relevant*	I can't assess
★	★	★	★	★	○

* 1.3.1.a. The risk categories that are valid for this criterion (select from the dropdown menu)

* 1.3.1.b. The tool's content is provided in a clear and appropriate way for the target audience (using an understandable, plain and simple language, with messages adapted to the user profile in terms of linguistic style and level, facilitating user understanding and avoiding using technicalities)

1 *I suggest this criterion is excluded*	2	3	4	5 *This criterion is extremely relevant*	I can't assess
★	★	★	★	★	○

* 1.3.1.b. The risk categories that are valid for this criterion (select from the dropdown menu)

* 1.3.1.c. There is sufficient information throughout the tool without any omissions, over-explanations, or irrelevant data

1 *I suggest this criterion is excluded*	2	3	4	5 *This criterion is extremely relevant*	I can't assess
☆	☆	☆	☆	☆	○

* 1.3.1.c. The risk categories that are valid for this criterion (select from the dropdown menu)

1.3.2. Credibility

* 1.3.2.a. The tool contains high quality information (e.g. text, feedback, charts, measures, references) from credible and legitimate sources (e.g., WHO)

1 *I suggest this criterion is excluded*	2	3	4	5 *This criterion is extremely relevant*	I can't assess
☆	☆	☆	☆	☆	○

* 1.3.2.a. The risk categories that are valid for this criterion (select from the dropdown menu)

* 1.3.2.b. The content has been reviewed by (or originated from) healthcare professionals

1 *I suggest this criterion is excluded*	2	3	4	5 *This criterion is extremely relevant*	I can't assess
☆	☆	☆	☆	☆	○

* 1.3.2.b. The risk categories that are valid for this criterion (select from the dropdown menu)

1.3.3. Content validity and reliability

* 1.3.3. The tool’s contents are relevant to the underlying objective and likely to be effective in achieving the specified purpose in the specific intended population

1 *I suggest this criterion is excluded*	2	3	4	5 *This criterion is extremely relevant*	I can't assess
★	★	★	★	★	○

* 1.3.3. The risk categories that are valid for this criterion (select from the dropdown menu)

1.3.o. Have we missed any important assessment criteria in this sub-category? Or do you have any comments to add to this sub-category?

1.4. Data management

1.4.1. Privacy and security

* 1.4.1.a. The tool has a clear privacy policy and informs the users on how their data will be kept confidential and secured and how the data may be used (e.g., for commercial or research purposes)

1 *I suggest this criterion is excluded*	2	3	4	5 *This criterion is extremely relevant*	I can't assess
★	★	★	★	★	○

* 1.4.1.a. The risk categories that are valid for this criterion (select from the dropdown menu)

* 1.4.1.b. The tool explicitly reports being compliant with the relevant data privacy and protection laws (e.g. GDPR, HIPAA...), and the treatment of any personal data is compatible with the Patient Data Act, Personal Data Act, and other applicable privacy laws

1 *I suggest this criterion is excluded*	2	3	4	5 *This criterion is extremely relevant*	I can't assess
★	★	★	★	★	○

* 1.4.1.b. The risk categories that are valid for this criterion (select from the dropdown menu)

* 1.4.1.c. The tool respects informed consent and allows the user to opt out of data collection (e.g. ability to configure the settings of their data storage, access, and management)

1 *I suggest this criterion is excluded*	2	3	4	5 *This criterion is extremely relevant*	I can't assess
★	★	★	★	★	○

* 1.4.1.c. The risk categories that are valid for this criterion (select from the dropdown menu)

1.4.2. Data integration and interoperability

* 1.4.2.a. The tool allows exchange of data with other apps, e-tools, wearable devices, electronic health records (ability to exchange data with other systems on a technical and policy level)

1 *I suggest this criterion is excluded*	2	3	4	5 *This criterion is extremely relevant*	I can't assess
★	★	★	★	★	○

* 1.4.2.a. The risk categories that are valid for this criterion (select from the dropdown menu)

* 1.4.2.b. The tool allows the user to move across different platforms (e.g. mobile app vs web app, iOS vs Android)

1 *I suggest this criterion is excluded*	2	3	4	5 *This criterion is extremely relevant*	I can't assess
★	★	★	★	★	○

* 1.4.2.b. The risk categories that are valid for this criterion (select from the dropdown menu)

1.4.3. Timeliness

* 1.4.3. The tool can be used in real time (i.e. real-time data tracking), e.g. if the user is experiencing a health issue, reducing waits and sometimes harmful delays for both those who receive and those who give care

1 *I suggest this criterion is excluded*	2	3	4	5 *This criterion is extremely relevant*	I can't assess
★	★	★	★	★	○

* 1.4.3. The risk categories that are valid for this criterion (select from the dropdown menu)

1.4.o. Have we missed any important assessment criteria in this sub-category? Or do you have any comments to add to this sub-category?

1.5. Design

1.5.1. Usability

* 1.5.1.a. It is easy to navigate through the tool (e.g. to move from one location to another and to move backwards, and the design is responsive to the screen size used)

1 *I suggest this criterion is excluded*	2	3	4	5 *This criterion is extremely relevant*	I can't assess
★	★	★	★	★	○

* 1.5.1.a. The risk categories that are valid for this criterion (select from the dropdown menu)

* 1.5.1.b. Learning to use the tool is easy and does not require a lot of time, appropriate explanations appear if needed

1 *I suggest this criterion is excluded*	2	3	4	5 *This criterion is extremely relevant*	I can't assess
★	★	★	★	★	○

* 1.5.1.b. The risk categories that are valid for this criterion (select from the dropdown menu)

1.5.2. Visual design

* 1.5.2.a. The visual design is appealing and has a harmonious look and feel (including colours, and fonts are appropriately sized for the target audience)

1 *I suggest this criterion is excluded*	2	3	4	5 *This criterion is extremely relevant*	I can't assess
☆	☆	☆	☆	☆	○

* 1.5.2.a. The risk categories that are valid for this criterion (select from the dropdown menu)

* 1.5.2.b. To tool's appearance is well structured, and important information is clear and stands out

1 *I suggest this criterion is excluded*	2	3	4	5 *This criterion is extremely relevant*	I can't assess
☆	☆	☆	☆	☆	○

* 1.5.2.b. The risk categories that are valid for this criterion (select from the dropdown menu)

1.5.o. Have we missed any important assessment criteria in this sub-category? Or do you have any comments to add to this sub-category?

eHealth assessment criteria - Delphi process, Round 1

2. Social Criteria

2.1. Human centricity

2.1.1. User engagement, customisability, tailoring, user control

* 2.1.1.a. The tool includes high quality interactive features (enables user input and reaction) and is presented in an engaging way (e.g., contains the right mix of video/audio/text/graphics)

1 *I suggest this criterion is excluded*	2	3	4	5 *This criterion is extremely relevant*	I can't assess
☆	☆	☆	☆	☆	○

* 2.1.1.a. The risk categories that are valid for this criterion (select from the dropdown menu)

* 2.1.1.b. The tool is customisable and allows the user to control all the necessary settings and features (e.g., notifications, alerts, sounds, colours, and fonts)

1 *I suggest this criterion is excluded*	2	3	4	5 *This criterion is extremely relevant*	I can't assess
☆	☆	☆	☆	☆	○

* 2.1.1.b. The risk categories that are valid for this criterion (select from the dropdown menu)

* 2.1.1.c. There's evidence for collaboration with users in the tool's development (e.g. a strong and balanced advisory board with clinical/patients/technical team members able to lead the product design and development)

1 *I suggest this criterion is excluded*	2	3	4	5 *This criterion is extremely relevant*	I can't assess
★	★	★	★	★	○

* 2.1.1.c. The risk categories that are valid for this criterion (select from the dropdown menu)

2.1.2. Behavioural change, persuasiveness

* 2.1.2.a. The tool is persuasive and aims at understanding what influences people’s behaviour and decision making, and then uses this information to design compelling user interactions by offering relevant and customisable therapeutic activities and encouraging users to complete them (e.g. through incentivization, gamification...)

1 *I suggest this criterion is excluded*	2	3	4	5 *This criterion is extremely relevant*	I can't assess
★	★	★	★	★	○

* 2.1.2.a. The risk categories that are valid for this criterion (select from the dropdown menu)

* 2.1.2.b. The tool provides appropriate ongoing feedback and appropriate call to action based on the user’s state and activities (e.g. provides guidance based on user-entered information)

1 *I suggest this criterion is excluded*	2	3	4	5 *This criterion is extremely relevant*	I can't assess
★	★	★	★	★	○

* 2.1.2.b. The risk categories that are valid for this criterion (select from the dropdown menu)

2.1.3. Equity, accessibility, inclusiveness

* 2.1.3.a. The tool's content and design are appropriate for the target audience and accessible to vulnerable populations (e.g. adjust text size, text to voice, colourblind colour scheme adjuster, any specificity for use by minors, offline features). i.e. It takes the user context into account and does not vary in quality because of personal characteristics such as disabilities or vision problems etc.

1 *I suggest this criterion is excluded*	2	3	4	5 *This criterion is extremely relevant*	I can't assess
☆	☆	☆	☆	☆	○

* 2.1.3.a. The risk categories that are valid for this criterion (select from the dropdown menu)

* 2.1.3.b. The tool takes into account culturally relevant factors (e.g. different languages and alphabets, specific religious or cultural requirements or restrictions)

1 *I suggest this criterion is excluded*	2	3	4	5 *This criterion is extremely relevant*	I can't assess
☆	☆	☆	☆	☆	○

* 2.1.3.b. The risk categories that are valid for this criterion (select from the dropdown menu)

2.1.4. Therapeutic alliance

* 2.1.4. The tool has the ability to foster the interaction between the health care professionals and their patients (e.g. communication features, feedback options)

1 *I suggest this criterion is excluded*	2	3	4	5 *This criterion is extremely relevant*	I can't assess
☆	☆	☆	☆	☆	○

* 2.1.4. The risk categories that are valid for this criterion (select from the dropdown menu)

2.1.o. Have we missed any important assessment criteria in this sub-category? Or do you have any comments to add to this sub-category?

2.2. Health outcomes

2.2.1. Health benefits and effectiveness

* 2.2.1. The tool has been trialled and tested in a real world setting, and its effectiveness was verified by evidence in published scientific literature (e.g. usability studies and user research)

1 *I suggest this criterion is excluded*	2	3	4	5 *This criterion is extremely relevant*	I can't assess
★	★	★	★	★	○

* 2.2.1. The risk categories that are valid for this criterion (select from the dropdown menu)

2.2.2. Evidence base

* 2.2.2. The tool's clinical effectiveness is supported by strong research (e.g. pre-registered RCTs - Randomised Controlled Trials) with adequate statistical power conducted by credible sources, in which the tool was found to be superior to an appropriate placebo or equivalent to acceptable evidence-based treatment groups

1 *I suggest this criterion is excluded*	2	3	4	5 *This criterion is extremely relevant*	I can't assess
★	★	★	★	★	○

* 2.2.2. The risk categories that are valid for this criterion (select from the dropdown menu)

2.2.3. Patient safety

* 2.2.3.a. The tool clearly identifies the risks that its management may pose for user safety and has gone through the proper certification processes to ensure its safety (e.g. software as a medical device, third-party certification by a medical or governmental organisation)

1 *I suggest this criterion is excluded*	2	3	4	5 *This criterion is extremely relevant*	I can't assess
★	★	★	★	★	○

* 2.2.3.a. The risk categories that are valid for this criterion (select from the dropdown menu)

* 2.2.3.b. The tool contains a disclaimer (or a statement of similar implication) that the information provided/content does not replace a health care professional’s judgment

1 *I suggest this criterion is excluded*	2	3	4	5 *This criterion is extremely relevant*	I can't assess
★	★	★	★	★	○

* 2.2.3.b. The risk categories that are valid for this criterion (select from the dropdown menu)

* 2.2.3.c. The tool warns about potential risks when necessary and properly handles potentially “dangerous” information entered by a patient (e.g. when it is necessary to consult a professional), i.e. avoiding injuries to patients from the care that is intended to help them

1 *I suggest this criterion is excluded*	2	3	4	5 *This criterion is extremely relevant*	I can't assess
★	★	★	★	★	○

* 2.2.3.c. The risk categories that are valid for this criterion (select from the dropdown menu)

2.2.o. Have we missed any important assessment criteria in this sub-category? Or do you have any comments to add this sub-category?

2.3. Visible popularity metrics

2.3.1. Ratings and user satisfaction

* 2.3.1. The tool's visible users’ reviews and ratings are favourable (e.g. a star rating above 4/5 stars). Using users’ perceived value through users’ reviews and ratings as a proxy for quality, usefulness, or acceptability and popularity.

1 *I suggest this criterion is excluded*	2	3	4	5 *This criterion is extremely relevant*	I can't assess
★	★	★	★	★	○

* 2.3.1. The risk categories that are valid for this criterion (select from the dropdown menu)

2.3.o. Have we missed any important assessment criteria in this sub-category? Or do you have any comments to add to this sub-category?

2.4. Social aspects

2.4.1. Social influence and endorsement

* 2.4.1.a. The tool has been verified, given a good review, or endorsed by a legitimate/reliable source such as a health organisation or health authority (e.g., APA; FDA in the US; NIH; NHS in the UK; NICE in the UK).

1 *I suggest this criterion is excluded*	2	3	4	5 *This criterion is extremely relevant*	I can't assess
★	★	★	★	★	○

* 2.4.1.a. The risk categories that are valid for this criterion (select from the dropdown menu)

* 2.4.1.b. The tool provides possibilities for peer support and/or social networking

1 *I suggest this criterion is excluded*	2	3	4	5 *This criterion is extremely relevant*	I can't assess
★	★	★	★	★	○

* 2.4.1.b. The risk categories that are valid for this criterion (select from the dropdown menu)

2.4.o. Have we missed any important assessment criteria in this sub-category? Or do you have any comments to add to this sub-category?

3. Organisational Criteria

3.1. Scalability and sustainability

3.1.1. Cost effectiveness

* 3.1.1. A cost-benefit analysis was performed and led to positive results. I.e. the balance between the costs and benefits arising from the tool's utilisation. This refers to the tool's direct costs (purchase price, subscription, licensing…), but may also include costs associated with the tool's selection, staff training, setting up support mechanisms, and appropriate governance

1 *I suggest this criterion is excluded*	2	3	4	5 *This criterion is extremely relevant*	I can't assess
☆	☆	☆	☆	☆	○

* 3.1.1. The risk categories that are valid for this criterion (select from the dropdown menu)

3.1.2. Maintenance

* 3.1.2. The tool gets periodic updates and maintenance both from technical and content perspectives (e.g. last update not older than 12 months)

1 *I suggest this criterion is excluded*	2	3	4	5 *This criterion is extremely relevant*	I can't assess
☆	☆	☆	☆	☆	○

* 3.1.2. The risk categories that are valid for this criterion (select from the dropdown menu)

3.1.3. Adoption and fidelity

* 3.1.3. The tool is implemented and utilised within the target health system under usual care OR a large group of clinicians officially refers patients to utilise it (this can be checked by looking at the unique monthly users and their percentage in relation to the target population in the target health system/market).

1 *I suggest this criterion is excluded*	2	3	4	5 *This criterion is extremely relevant*	I can't assess
☆	☆	☆	☆	☆	○

* 3.1.3. The risk categories that are valid for this criterion (select from the dropdown menu)

3.1.4. Availability

* 3.1.4. The tool and its data can be accessed at any time and on different platforms and operating systems (e.g., Android, iOS...)

1 *I suggest this criterion is excluded*	2	3	4	5 *This criterion is extremely relevant*	I can't assess
★	★	★	★	★	○

* 3.1.4. The risk categories that are valid for this criterion (select from the dropdown menu)

3.1.o. Have we missed any important assessment criteria in this sub-category? Or do you have any comments to add to this sub-category?

3.2. Healthcare organisation

3.2.1. Implementation

* 3.2.1. Assesses the extent to which the tool can be implemented as intended (i.e., feasibility of implementing the tool at a pre-determined date and time). This can be checked by looking at how long it takes, on average, from the contractual agreement until the tool is fully up and running in a healthcare organisation

1 *I suggest this criterion is excluded*	2	3	4	5 *This criterion is extremely relevant*	I can't assess
★	★	★	★	★	○

* 3.2.1. The risk categories that are valid for this criterion (select from the dropdown menu)

3.2.2. Workforce and resources

* 3.2.2. Assesses the workforce and resources required to scale-up the tool, and the implications for care process and care management (e.g. does the tool fit well into existing workflows, roles and responsibilities, or requires new workflows, new roles etc.)

1 *I suggest this criterion is excluded*	2	3	4	5 *This criterion is extremely relevant*	I can't assess
★	★	★	★	★	○

* 3.2.2. The risk categories that are valid for this criterion (select from the dropdown menu)

3.2.3. Infrastructure

* 3.2.3. Assesses the readiness of the necessary infrastructure for the tool's implementation. i.e. Whether the tool can fit into existing infrastructure or would require investment in additional infrastructure (if yes, is it low, medium, or high investment)

1 *I suggest this criterion is excluded*	2	3	4	5 *This criterion is extremely relevant*	I can't assess
★	★	★	★	★	○

* 3.2.3. The risk categories that are valid for this criterion (select from the dropdown menu)

3.2.o. Have we missed any important assessment criteria in this sub-category? Or do you have any comments to add to this sub-category?

3.3. Healthcare context

3.3.1. Strategic, political, and environmental contexts

* 3.3.1. How favourable are the pre-conditions (strategic, political, and environmental contexts) that influence the scaling up of the eHealth tool. For example, the tool's suitability to the socioeconomic context in question, considerations of foreign languages that the tool needs to support, literacy level, and the local regulatory environment

1 *I suggest this criterion is excluded*	2	3	4	5 *This criterion is extremely relevant*	I can't assess
★	★	★	★	★	○

* 3.3.1. The risk categories that are valid for this criterion (select from the dropdown menu)

3.3.o. Have we missed any important assessment criteria in this sub-category? Or do you have any comments to add to this sub-category?

3.4. Developer

3.4.1. Transparency and credibility

* 3.4.1.a. Contact details of the tool's provider are available, easy to find, and include office address, email, and team members details

1 *I suggest this criterion is excluded*	2	3	4	5 *This criterion is extremely relevant*	I can't assess
★	★	★	★	★	○

* 3.4.1.a. The risk categories that are valid for this criterion (select from the dropdown menu)

* 3.4.1.b. Availability of information and credentials of the individuals and organisations involved in the development and funding of the tool (transparency on the involvement of any parties that may lead to conflict of interest, e.g. commercial sponsors and partners, financial disclosure)

1 *I suggest this criterion is excluded*	2	3	4	5 *This criterion is extremely relevant*	I can't assess
★	★	★	★	★	○

* 3.4.1.b. The risk categories that are valid for this criterion (select from the dropdown menu)

3.4.2. Compliance and accountability

* 3.4.2. The provider respects ethical conduct, clinical responsibility, and the rules and regulations protecting patient’s rights and societal interests (e.g., the tool was approved or certified by a regulatory body in the case of software as a medical device, GDPR, HIPAA...)

1 *I suggest this criterion is excluded*	2	3	4	5 *This criterion is extremely relevant*	I can't assess
★	★	★	★	★	○

* 3.4.2. The risk categories that are valid for this criterion (select from the dropdown menu)

3.4.3. Proactivity and interaction quality

* 3.4.3.a. Interaction quality between the provider and the users, including responsiveness, after sales services, and customer orientation (e.g. provider responds to direct requests/messages swiftly and professionally)

1 *I suggest this criterion is excluded*	2	3	4	5 *This criterion is extremely relevant*	I can't assess
★	★	★	★	★	○

* 3.4.3.a. The risk categories that are valid for this criterion (select from the dropdown menu)

* 3.4.3.b. Demonstration of excellence in a proactive approach to the assessment of user needs, and continuous learning (e.g. provider continuously takes user feedback into account in the periodic updates and iterations of the tool and communicates this to the users)

1 *I suggest this criterion is excluded*	2	3	4	5 *This criterion is extremely relevant*	I can't assess
★	★	★	★	★	○

* 3.4.3.b. The risk categories that are valid for this criterion (select from the dropdown menu)

3.4.4. History of producing safe health products

* 3.4.4. The tool's provider has specific experience in the eHealth field OR academic institution (e.g., university) OR health care system (or large health providers' organisation).

1 *I suggest this criterion is excluded*	2	3	4	5 *This criterion is extremely relevant*	I can't assess
★	★	★	★	★	○

* 3.4.4. The risk categories that are valid for this criterion (select from the dropdown menu)

3.4.o. Have we missed any important assessment criteria in this sub-category? Or do you have any comments to add to this sub-category?

Thank you for your valuable time and contribution. You will hear from the research team as soon as Round 1 results have been summarised so we can schedule an interview with you to discuss the initial results and how to take this effort forward and make it accessible and easy to use to the relevant stakeholders.

Supplementary Reference 3: Copy of Round 2 Survey (8 pages)

Innovation project supported by
Schweizerische Eidgenossenschaft
Confédération suisse
Confederazione Svizzera
Confederaziun svizra
Swiss Confederation
Innosuisse – Swiss Innovation Agency

Fachhochschule Nordwestschweiz

eHealth assessment criteria - Delphi process, Round 2

Finalising the assessment criteria

Thanks again for participating in the study and taking the time to fill Round 1 survey. This is the final step in finalising the list of assessment criteria based on your input. This final survey takes about 15 minutes to complete.

Why a second round?

The Delphi methodology is a consensus based process, it allows for reflection among participants, who are able to nuance and reconsider their opinion based on the anonymised opinions of others. Therefore, this final round aims to:

- confirm the definite exclusion of the criteria that did not meet the pre-defined consensus in Round 1 by corroborating experts' assessment of these criteria (most of these have been slightly reworded or expanded based on expert feedback in the first round)
- validate the relevance of the additional expert-suggested criteria that were deemed to be missing in the first round

A criteria is considered a must-have criteria only if 75% of the experts assess its relevance at 4 or 5 on the Likert scale.

Hence, the objectives of Round 2 survey are:

01	Criteria that did not meet consensus	• Final assessment of the <u>**17 criteria**</u> **that did not meet the predefined 75% consensus** in Round 1<br>• These have sometimes been **slightly reworded or expanded** based on expert feedback in Round 1<br>• Experts will **not** have to reassess risk levels again
02	Additional criteria	• Assessment of the **additional criteria suggested by the experts** in Round 1<br>• <u>**9 new criteria**</u> to assess and to define the respective risk level

Reminder: This project focuses solely on assessing **patient-facing eHealth tools**, including self-management tools and remote eHealth solutions, rather than tools used within and between care providers (e.g., Electronic Health Records), digital biomarkers, or health data analytics systems used at population level.

Disclaimer: This research project is intended for educational purposes only and is not intended as legal advice. Payers have differing coverage and reimbursement policies. Laws, regulations, and health insurance policies concerning coverage, coding, and reimbursement are complex and are evolving rapidly. For legal advice, please consult with legal counsel.

This study is part of an overarching research project that is jointly sponsored by F. Hoffmann-La Roche Ltd, KPT insurance, and Innosuisse (the Swiss Innovation Agency, grant 104.445 IP-ICT).

Final assessment of the criteria that did not meet expert consensus in Round 1

*Most of the criteria have been slightly reworded or expanded based on the experts' feedback in Round 1. Any **changes have been marked in bold** to make it easy for you to spot them.*

* 1.1.4. The tool allows the possibility to give instant feedback to the developers (e.g., provider messaging to report technical issues or errors, **inaccuracies or inconsistent workflows**)

1 *I suggest this criterion is excluded*	2	3	4	5 *This criterion is extremely relevant*	I can't assess
★	★	★	★	★	○

* 1.2.2.b. The tool considers multiple health issues and related ones, and sufficiently addresses them to help meet the intended purpose **without overwhelming the user (i.e. consider comorbidities, and features that may improve overall quality of life, e.g. adding breathing exercises in a remote patient monitoring tool for lung cancer patients)**

1 *I suggest this criterion is excluded*	2	3	4	5 *This criterion is extremely relevant*	I can't assess
★	★	★	★	★	○

* 1.3.1.c. There is sufficient information throughout the tool without any omissions, over-explanations, or irrelevant data

1 *I suggest this criterion is excluded*	2	3	4	5 *This criterion is extremely relevant*	I can't assess
★	★	★	★	★	○

* 1.4.2.b. The tool allows the users to move across different platforms **to allow portability of the tool while retaining their data** (e.g. mobile app vs web app, iOS vs Android, **older smartphones**)

1 *I suggest this criterion is excluded*	2	3	4	5 *This criterion is extremely relevant*	I can't assess
★	★	★	★	★	○

* 1.4.3. The tool can be used in real time (i.e. real-time data tracking), e.g. if the user is experiencing a health issue, reducing waits and sometimes harmful delays for both those who receive and those who give care

1 *I suggest this criterion is excluded*	2	3	4	5 *This criterion is extremely relevant*	I can't assess
★	★	★	★	★	○

* 2.1.1.a. The tool includes high quality interactive features (enables user input and reaction) and is presented in an engaging way (e.g., contains the right mix of video/audio/text/graphics)

1 *I suggest this criterion is excluded*	2	3	4	5 *This criterion is extremely relevant*	I can't assess
★	★	★	★	★	○

* 2.1.1.b. The tool is customisable and allows the user to control all the necessary settings and features (e.g., notifications, alerts, sounds, colours, and fonts) **except for the features that form an essential part of an intervention (e.g. the tool allows the patients to customise the time of a certain reminder according to their daily routine but doesn't allow them to remove it)**

1 *I suggest this criterion is excluded*	2	3	4	5 *This criterion is extremely relevant*	I can't assess
★	★	★	★	★	○

* 2.1.2.a. The tool is persuasive and aims at understanding what influences people’s behaviour and decision making, and then uses this information to design compelling user interactions by offering relevant and customisable therapeutic activities and encouraging users to complete them (e.g. through incentivisation, gamification etc.) **in a way that balances engagement vs tool addiction**

1 *I suggest this criterion is excluded*	2	3	4	5 *This criterion is extremely relevant*	I can't assess
☆	☆	☆	☆	☆	○

* 2.3.1. The tool's visible **and verified** users’ reviews and ratings are favourable (e.g. a star rating above 4/5 stars **in the app store, or the Net Promoter Score - NPS**). Using users’ perceived value through users’ reviews and ratings as a proxy for quality, usefulness, or acceptability and popularity

1 *I suggest this criterion is excluded*	2	3	4	5 *This criterion is extremely relevant*	I can't assess
☆	☆	☆	☆	☆	○

* 2.4.1.a. The tool has been verified, given a good review, or endorsed by a legitimate/reliable source such as a health organisation or health authority (e.g. APA; FDA in the US; NIH; NHS in the UK; NICE in the UK) **or recommended by trusted Healthcare Professionals**

1 *I suggest this criterion is excluded*	2	3	4	5 *This criterion is extremely relevant*	I can't assess
☆	☆	☆	☆	☆	○

* 2.4.1.b. The tool provides possibilities for peer support and/or social networking **(e.g. anecdotal evidence - a space to share experiences like patient forums, groups etc.) and/or supported by patient organisations or advisory groups**

1 *I suggest this criterion is excluded*	2	3	4	5 *This criterion is extremely relevant*	I can't assess
☆	☆	☆	☆	☆	○

* 3.1.3. The tool is implemented and utilised within the target health system under usual care OR a large group of clinicians officially refers patients to utilise it (this can be checked by looking at the unique monthly users and their percentage in relation to the target population in the target health system/market). **The size of the target population needs to be evidence based**

1 *I suggest this criterion is excluded*	2	3	4	5 *This criterion is extremely relevant*	I can't assess
☆	☆	☆	☆	☆	○

* 3.2.1. Assesses the extent to which the tool can be implemented as intended (i.e., feasibility of implementing the tool at a pre-determined date and time). This can be checked by looking at how long it takes, on average, from the contractual agreement until the tool is fully up and running in a healthcare organisation

1 *I suggest this criterion is excluded*	2	3	4	5 *This criterion is extremely relevant*	I can't assess
☆	☆	☆	☆	☆	◯

* 3.3.1. How favourable are the pre-conditions (strategic, political, and environmental contexts) that influence the scaling up of the eHealth tool. For example, the tool’s suitability to the socioeconomic context in question, considerations of foreign languages that the tool needs to support, literacy level, and the local regulatory environment **such as reimbursement**

1 *I suggest this criterion is excluded*	2	3	4	5 *This criterion is extremely relevant*	I can't assess
☆	☆	☆	☆	☆	◯

* 3.4.1.a. Contact details of the tool's provider are available, easy to find, and include office address, email, and team members details

1 *I suggest this criterion is excluded*	2	3	4	5 *This criterion is extremely relevant*	I can't assess
☆	☆	☆	☆	☆	◯

* 3.4.1.b. Availability of information and credentials of the individuals and organisations involved in the development and funding of the tool (transparency on the involvement of any parties that may lead to conflict of interest, e.g. commercial sponsors and partners, financial disclosure)

1 *I suggest this criterion is excluded*	2	3	4	5 *This criterion is extremely relevant*	I can't assess
☆	☆	☆	☆	☆	◯

* 3.4.4. The tool's provider **and/or its core team members** have specific experience in the eHealth field OR academic institution (e.g., university) OR health care system (or large health providers’ organisation). **E.g. an experienced medical director that oversees the algorithms / contents / features of the tool**

1 *I suggest this criterion is excluded*	2	3	4	5 *This criterion is extremely relevant*	I can't assess
☆	☆	☆	☆	☆	◯

Please let us know if you have any comments (kindly note that no new criteria will be added at this stage as there won't be a chance to validate them with the expert panel)

Assessment of the additional criteria suggested by the experts in Round 1

* 1.3.1.d. The content has been reviewed by patients to ensure readability and acceptability

1 *I suggest this criterion is excluded*	2	3	4	5 *This criterion is extremely relevant*	I can't assess
☆	☆	☆	☆	☆	○

* 1.3.1.d. The risk categories that are valid for this criterion (select from the dropdown menu)

* 1.4.1.d. The tool explicitly and easily enables users to delete their data

1 *I suggest this criterion is excluded*	2	3	4	5 *This criterion is extremely relevant*	I can't assess
☆	☆	☆	☆	☆	○

* 1.4.1.d. The risk categories that are valid for this criterion (select from the dropdown menu)

* 1.4.3.b. The tool is reliable and available at all times and can handle high levels of traffic and usage, with backup and recovery measures in case of downtime or system failures (e.g. enabling offline functionality, functioning during energy interruption or under difficult environmental conditions)

1 *I suggest this criterion is excluded*	2	3	4	5 *This criterion is extremely relevant*	I can't assess
☆	☆	☆	☆	☆	○

* 1.4.3.b. The risk categories that are valid for this criterion (select from the dropdown menu)

* 1.4.4. The tool compliantly allows for data sharing and segregation for research use (including data analytics and reporting, quality improvement initiatives, and clinical trials)

1 *I suggest this criterion is excluded*	2	3	4	5 *This criterion is extremely relevant*	I can't assess
★	★	★	★	★	○

* 1.4.4. The risk categories that are valid for this criterion (select from the dropdown menu)

* 1.4.5. Data findability and retrievability: Metadata definition (including e.g. units used, reference to controlled vocabularies, ontologies) and the ability for users to retrieve data with the same granularity specified by the metadata (up to and including raw data sets)

1 *I suggest this criterion is excluded*	2	3	4	5 *This criterion is extremely relevant*	I can't assess
★	★	★	★	★	○

* 1.4.5. The risk categories that are valid for this criterion (select from the dropdown menu)

* 1.5.1.c. The tool is usable and accessible in the intended clinical setting (e.g. if it is used by HCPs in a clinical setting, can they use it while wearing gloves, e.g. are buttons big enough and separated)

1 *I suggest this criterion is excluded*	2	3	4	5 *This criterion is extremely relevant*	I can't assess
★	★	★	★	★	○

* 1.5.1.c. The risk categories that are valid for this criterion (select from the dropdown menu)

* 2.1.3.c. Affordability of the tool taking into account the local socioeconomic context, and clarity on who pays and how do they pay

1 *I suggest this criterion is excluded*	2	3	4	5 *This criterion is extremely relevant*	I can't assess
★	★	★	★	★	○

* 2.1.3.c. The risk categories that are valid for this criterion (select from the dropdown menu)

* 2.2.3.d. The tool differentiates between clinical and technical feedback, and clearly channels clinical feedback that may pose a health risk through the proper channels (e.g. advising the patient to call their care team, go to the ER....) and reviews them for vigilance and post-market surveillance purposes and, where relevant, notify them to competent authorities

1 *I suggest this criterion is excluded*	2	3	4	5 *This criterion is extremely relevant*	I can't assess
★	★	★	★	★	○

* 2.2.3.d. The risk categories that are valid for this criterion (select from the dropdown menu)

* 3.4.5. Availability of phase-out scenarios, if the tool's provider stops producing/maintaining the tool

1 *I suggest this criterion is excluded*	2	3	4	5 *This criterion is extremely relevant*	I can't assess
★	★	★	★	★	○

* 3.4.5. The risk categories that are valid for this criterion (select from the dropdown menu)

Please let us know if you have any comments (kindly note that no new criteria will be added at this stage as there won't be a chance to validate them with the expert panel)

Thank you for your valuable time and contribution. You will hear from the research team as soon as the final results have been peer reviewed to ask for your consent (or not) to be publicly recognised as a contributing expert in the planned white paper.
